# Shadow Enhancers Are Pervasive Features of Developmental Regulatory Networks

**DOI:** 10.1016/j.cub.2015.11.034

**Published:** 2016-01-11

**Authors:** Enrico Cannavò, Pierre Khoueiry, David A. Garfield, Paul Geeleher, Thomas Zichner, E. Hilary Gustafson, Lucia Ciglar, Jan O. Korbel, Eileen E.M. Furlong

**Affiliations:** 1 European Molecular Biology Laboratory (EMBL), Genome Biology Unit, 69117 Heidelberg, Germany

**Keywords:** enhancer, redundancy, shadow enhancer, robustness, transcriptional networks, development

## Abstract

Embryogenesis is remarkably robust to segregating mutations and environmental variation; under a range of conditions, embryos of a given species develop into stereotypically patterned organisms. Such robustness is thought to be conferred, in part, through elements within regulatory networks that perform similar, redundant tasks. Redundant enhancers (or “shadow” enhancers), for example, can confer precision and robustness to gene expression, at least at individual, well-studied loci. However, the extent to which enhancer redundancy exists and can thereby have a major impact on developmental robustness remains unknown. Here, we systematically assessed this, identifying over 1,000 predicted shadow enhancers during *Drosophila* mesoderm development. The activity of 23 elements, associated with five genes, was examined in transgenic embryos, while natural structural variation among individuals was used to assess their ability to buffer against genetic variation. Our results reveal three clear properties of enhancer redundancy within developmental systems. First, it is much more pervasive than previously anticipated, with 64% of loci examined having shadow enhancers. Their spatial redundancy is often partial in nature, while the non-overlapping function may explain why these enhancers are maintained within a population. Second, over 70% of loci do not follow the simple situation of having only two shadow enhancers—often there are three (*rols*), four (*CadN* and *ade5*), or five (*Traf1*), at least one of which can be deleted with no obvious phenotypic effects. Third, although shadow enhancers can buffer variation, patterns of segregating variation suggest that they play a more complex role in development than generally considered.

## Highlights

• Regulation by shadow enhancers is pervasive and complex during embryonic development
• The vast majority of genes have more than two enhancers with similar activity
• Shadow enhancers buffer genetic variation within a population, but yet appear conserved
• Evolutionary analyses suggest that they play complex and fundamental roles in development

## Teaser

Cannavò et al. examine redundant (shadow) enhancers genome wide, finding that the majority of loci have more than two elements with similar activity. Evolutionary analyses show evidence of pervasive stabilizing selection and an ability to buffer mutations, suggesting that shadow enhancers have complex and fundamental roles in developmental networks

## Introduction

Developmental robustness is achieved through buffering gene expression patterns against stochastic, genetic, and environmental perturbations [1, 2, 3, 4, 5, 6, 7, 8, 9]. Although the underlying molecular mechanisms are still being dissected, transcriptional robustness can be modulated at several levels [10, 11], including DNA accessibility [12], RNA polymerase II pausing [13, 14], and promoter organization [15, 16, 17]. It can also arise from higher levels of network organization [18, 19, 20, 21, 22], including functional redundancy, defined as two parts of a system that can perform the same or similar tasks and are therefore not individually essential [23].

A potential contributor to functional redundancy is regulatory elements with overlapping functions. A number of studies in vertebrates [4, 8], invertebrates [2, 24], and plants [25] have identified enhancers that appear to act redundantly—defined as two enhancers that drive similar patterns of expression and in which deletion of one did not cause any obvious aberrant phenotypes [4, 8]. There are a number of well-characterized examples of such shadow enhancers acting during embryonic development [2]. In the *pax3* locus, for example, two enhancers direct expression in neural crest cells [1, 2, 3, 4, 5, 6, 7, 8, 9]. Although the proximal 5′ element, when placed upstream of *pax3* cDNA, is sufficient to rescue neural crest cell development in mice lacking endogenous *pax3*, this enhancer is not required for development or viability. Similarly in the *TCRgamma* locus, deletion of either the HsA or 3′E (Cgamma1) enhancers has little effect on *TCRgamma* transcription, whereas deletion of both elements causes a severe reduction in transcription and defects in gammadelta thymocyte development [9]. Interestingly, although both enhancers act redundantly in gammadelta thymocytes, in a different cell context, the HsA enhancer acts non-redundantly with the 3′E element to regulate gene expression [9].

Although examples of redundant enhancers have been known for over 20 years, recent studies in *Drosophila* have reignited the debate over the prevalence and functional role of these elements in the regulation of gene expression. When examining the binding patterns of three transcription factors (TFs), Hong et al. observed that in addition to a gene’s well-characterized enhancer, many early patterning genes in *Drosophila* have a second element with very similar TF occupancy [2]. These shadow enhancers frequently regulate highly similar, overlapping patterns of expression in transgenic reporter assays, suggesting that they act redundantly [2, 5, 6]. For example, each of the five gap gene loci in the *Drosophila* segmentation pathway contain an additional shadow enhancer [7]. Shadow enhancers can provide robustness to genetic variation within a population, allowing development to proceed unperturbed, as shown at a number of well-characterized loci [4, 8]. However, whether this is their primary function remains unclear as they appear to have multiple functions in the regulation of gene expression. For example, in some cases, enhancers that appear to act redundantly due to their overlapping activity are actually both essential to define the precise spatial, in the case of *snail* [26], or temporal, in the case of *brinker* [27], pattern of that gene’s expression. Alternatively, they may act redundantly, controlling the levels of a gene’s expression at one stage of lifespan (e.g., in adults), but act more synergistically during another (e.g., embryogenesis), as recently observed at the mouse *Pomc* locus [28]. Similarly, enhancers that appear to act redundantly under normal environmental conditions can be essential under more stressful conditions, as demonstrated in the *shavenbaby* (*svb*) [3] and *snail* [5] loci. Genes with redundant enhancers also tend to initiate their expression more synchronously during very rapid cell divisions, illustrating another context in which these elements help ensure robust expression during development [7]. These examples question the extent to which enhancers with redundant activity in one context are completely redundant across the entire spectrum of the enhancer’s activity (which we refer to as absolute redundancy).

The examples above demonstrate that individual enhancers can act to canalize their target gene’s expression, buffering them against environmental and genetic perturbations. However, for shadow enhancers to act as major contributors to developmental robustness, they should be much more prevalent than the handful of examples known to date. Just how extensive redundant enhancers are, and to what extent overlapping enhancers are truly redundant, remains unclear. To directly assess this, we performed the first genome-wide assessment of the prevalence and global properties of shadow enhancers using the developing *Drosophila* mesoderm as a model system. Using two stringent approaches, we identified 1,055 shadow enhancers associated with 319 unique genes. For 23 enhancers at five loci, we examined their in vivo activity throughout all stages of embryonic development. This revealed a regulatory landscape that is considerably more complex than the simple “one shadow to one main enhancer” relationship. Rather, the majority of loci contain three, four, or even as many as five shadow enhancers. When one shadow enhancer is deleted in each of these five loci, there was little obvious effect on embryonic development, suggesting that they can buffer the effects of genetic variation and are thus redundant. However, contrary to expectations for enhancers with absolute redundancy, shadow enhancers are more conserved than non-redundant enhancers, show a higher proportion of functional sites, and show neither evidence of relaxed selection in natural populations nor enrichment for lineage-specific adaptive events, observations that are most consistent with pervasive stabilizing selection. These conservation patterns may be a result of selection for robustness per se [29]. Alternatively, they may equally be a side product of the modular nature of developmental programs—when multiple enhancers are required to regulate complex patterns of expression, a degree of robustness may be an inevitable, very useful, byproduct.

## Results

### Enhancers with Complete Spatial Redundancy Are Rare, Whereas Partial Redundancy Is Common

The term redundancy, where two parts have the same function, is generally perceived as absolute redundancy. However, the examples presented above show clear cases in which enhancers act 100% redundantly in one context (tissue A, time point 1, or normal environmental conditions) and yet are essential in another [tissue B (e.g., HsA [9] and *snail* [26]), time point 2 (e.g., *brinker* [27]), or adverse environmental conditions (e.g., *svb* [3] and *snail* [5])], a property we refer to as partial redundancy. Enhancers with absolute redundancy are often generated through duplication events [30] and then either functionally diverge or degrade, being rapidly lost within a population. Partially redundant elements, i.e., enhancers with overlapping spatial activity, in contrast, should be maintained by selection and therefore preserved over longer evolutionary timescales (e.g., [31]) and thus should be more common.

**Figure 1:**
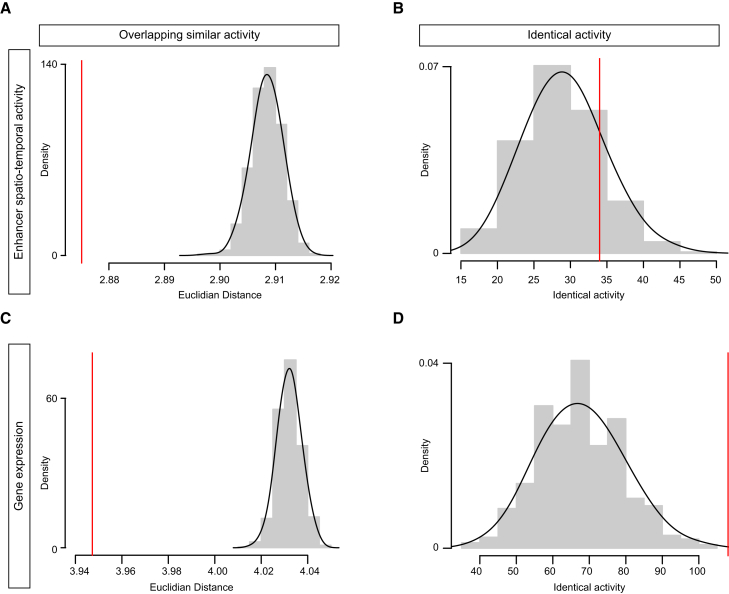
Frequency of Enhancers Pairs with Similar versus Identical Activity The level of similarity (partial overlap) in tissue expression of enhancers (A and B) and genes (C and D) within 50 kb windows of each other, compared to what would be expected by chance. Vertical red line represents the observed data, and the histogram and associated density plots show the values achieved from randomly shuffling enhancers/genes in the genome. (A) The Euclidean distance was used to summarize the (multidimensional) distance between pairs of enhancers in tissue expression space. Plotted here is the median Euclidean distance for the observed (red line) and expected (gray histogram) distribution of enhancers with similar tissue expression. (B) Number of co-located enhancers with identical spatiotemporal activity. (C) Median Euclidean distance (measure of similarity in multidimensional tissue expression) between co-located genes (within 50 kb of each other). (D) Number of co-located genes with identical patters of expression.

It is now possible to assess this reasoning, given the recent availability of a very large collection of 7,705 enhancers covering ∼15% of the non-coding *D. melanogaster* genome, whose detailed in vivo activity was annotated with 227 tissue terms throughout all stages of *Drosophila* embryogenesis in stable transgenic embryos [32]. We therefore first determined whether enhancers with overlapping spatial activity (partial redundancy) are more prevalent within a genome compared to enhancers with identical activity (absolute redundancy). Only enhancers with a single DNaseI-hypersensitive (DHS) site were included in the analysis, to exclude ambiguity caused by cases where multiple enhancers may be contained within the same 2 kb region tested in transgenic embryos (Supplemental Experimental Procedures). Overall, enhancers located within 50 kb of each other are much more likely to exhibit similar, overlapping spatial activity (p = 2.3 × 10^−34^; Figure 1A; Supplemental Experimental Procedures). However, they are not more likely to exhibit identical activity than expected by chance (p = 0.79; Figure 1B). As expected, these results indicate that even when considering a very broad and diverse set of spatiotemporal patterns, absolute redundancy of enhancer activity for spatial expression is rare, though some level of redundancy (overlapping spatial activity) is present and likely to be functionally important. Interestingly, this is not the case at the gene level. Genes within a 50 kb window of each other are both more likely to have overlapping spatial expression (p = 2.6 × 10^−61^) and identical expression (p = 0.001) than is expected by chance (Figures 1C and 1D; Supplemental Experimental Procedures).

### Genome-wide Identification of Enhancers with Highly Correlated Activity

**Figure 2:**
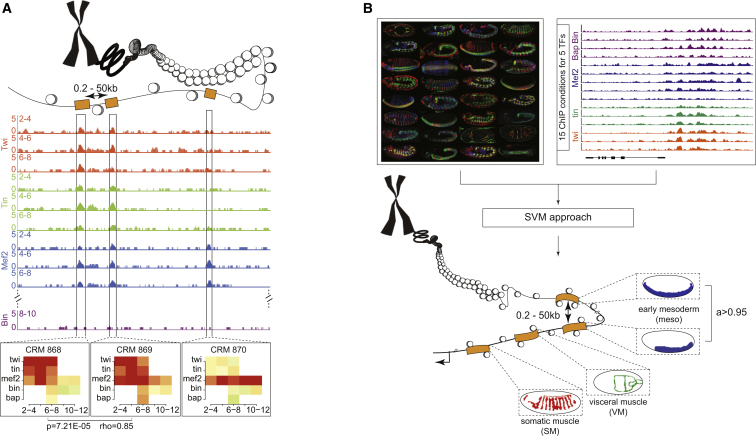
Genome-wide Prediction of Shadow Enhancers Two complementary criteria used to identify shadow enhancers throughout the genome: (A) Enhancers with highly correlated TF occupancy, using ChIP data from 15 conditions for mesoderm/muscle TFs [33], within 50 kb of each other and an associated gene with mesoderm and/or muscle expression. An example of a locus with two enhancers (CRM868 and CRM869) that have highly correlated binding (rho = 0.85; predicted shadow enhancers) compared to one that is not (CRM870) is shown. The bottom panels show the similarity (or dissimilarity) of TF binding as heatmaps using the ChIP peak height (red, high, to white, unbound), with TFs indicated on the y axis and developmental time (hr) on the x axis. (B) Enhancers with similar activity, predicted for all 8,008 mesodermal enhancers using a support vector machine (SVM) [33]. Enhancers within a 50 kb window of each other, with the same predicted expression (SVM score ≥0.95) and associated with a gene expressed in the same tissue were defined as shadow enhancers. The predicted tissue expression of enhancers is represented by cartoons. In (A) and (B), ChIP-chip data for the TFs Twist (red), Tin (green), Mef2 (blue), and Bin (purple) at different developmental time windows (2–4 hr, 4–6 hr, 6–8 hr, and 8–10 hr) are shown.

The analyses above suggest that enhancers with partially redundant activity are much more frequent than enhancers with absolute redundant activity. However, the frequency of these elements throughout the entire genome remains unclear; the authors identified 16 genes (out of 116 examined) with shadow enhancers [32]. To examine the prevalence of shadow enhancers more globally, we used two stringent approaches, focusing on the mesoderm and its derivatives. The first approach is based on Perry et al. [7], who defined prospective shadow enhancers for eight gap genes as pairs of genomic regions cobound by the same TFs within 100 kb of the genes’ promoter. Here, we extended this approach and more formally identified highly correlated TF occupancy across 15 conditions using chromatin immunoprecipitation (ChIP) data for five mesodermal TFs across multiple developmental stages [33]. Importantly, 97% of these ChIP-defined *cis*-regulatory modules (CRMs) function as developmental enhancers when tested in vivo using transgenic reporter assays [33]. Spearman rank-correlations between TF ChIP intensities was scanned across all 8,008 ChIP-defined enhancers within a 50 kb distance of each other (illustrated in Figure 2A) and in the vicinity of a gene with mesoderm and/or muscle expression, using in situ hybridization data (Supplemental Experimental Procedures). This identified a stringent set of shadow enhancers with highly correlated TF occupancy to at least one other enhancer associated to the same target gene (Table S1). An example of one such pair is shown in Figure 2A.

Although enhancers bound by the same combination of TFs often give rise to similar patterns of expression, a number of studies indicate more complex relationships. Enhancers with diverse patterns of TF occupancy [33, 34, 35] and regulatory logic [36] can, for example, also give rise to highly similar spatial activity. As the functional output of an enhancer is the important property for development, this is the parameter most likely under selection. This observation led us to our second approach, were we defined shadow enhancers based on their overlapping spatial activity. As there are no genome-wide data for enhancer spatiotemporal activity, we made use of our previously validated method, which predicted the activity of 8,008 mesodermal enhancers from TF occupancy data using a machine-learning approach trained on enhancers with characterized activity [33]. Each of the 8,008 ChIP-defined enhancers thereby has a probability score of being active in one of four exclusive tissue classes (Supplemental Experimental Procedures); importantly 83% of these tissue predictions hold true, i.e., the enhancers drove expression in the predicted tissue when tested in vivo in transgenic embryos [33] (Figure 2B). Shadow enhancers were defined as pairs of elements having a high-confidence prediction within the same tissue (SVM specificity score ≥0.95), being within 50 kb of each other, and associated with a common gene with overlapping expression from in situ hybridization (Figure 2B; Supplemental Experimental Procedures). This resulted in a stringent set of 866 shadow enhancers associated to 298 genes with mesoderm and/or muscle expression (Table S1).

The combination of these two approaches identified 1,055 shadow enhancers predicted to have similar activity to at least one other enhancer during mesoderm and/or muscle development (Table S1). Approximately 40% of genes are regulated by a single pair of shadow enhancers, in keeping with the vast majority of current examples of redundant enhancers in both *Drosophila* [2, 6, 7] and mice [1, 2, 3, 4, 5, 6, 7, 8, 9], with the notable exception of *vnd* [37]. However, the majority of genes appear to have much more complex regulation, with ∼60% of loci with shadow enhancers containing three (77 genes) or four (40 genes), and even a few examples of five (14 genes), six (ten genes), seven (seven genes), or eight (two genes), shadow enhancers with (predicted) similar activity (Figure S1A), suggesting that the current view of potential redundancy is over simplistic.

### Shadow Enhancers Can Buffer the Effects of Natural Sequence Variation

By definition, redundant or partially redundant enhancers can compensate for mutations that render one of the enhancers dysfunctional, as shown in the *svb* [3] and *dac* [5] loci in *D. melanogaster* or the *Hoxd* loci in mouse [4, 8]. If the shadow enhancers are acting redundantly, the transcriptional program driving embryogenesis should be able to proceed if one of the two enhancers is deleted. To examine this, we used natural sequence variation within a wild population of *Drosophila* to determine whether enhancers within a predicted redundant pair are affected by deleterious mutations. As it is often difficult to predict the effect of an individual SNP on TF occupancy [38, 39], we focused here on deletions (structural variations, SVs) greater than 50 bp that intersected the center of the enhancer and deleted at least 25% of its size. For this, we took advantage of a set of 205 fully sequenced inbred homozygous lines from Drosophila Genetic Reference Panel (DGRP) [40] and extended SV calls that we generated on 40 lines [41, 42] to all 205 lines and combined these with the Berkeley Drosophila Genome Project (BDGP) consortium’s freeze2 calls [41, 42] (Supplemental Experimental Procedures; Table S4).

We first examined how often SVs affect different functional parts of the genome, such as exons, introns, and enhancers (Figure S1B). To assess the significance of these results, we performed simulations in which SVs were randomly moved 1,000 times by up to 50 kb up- or downstream, and then reassessed the overlap with the functional elements for each iteration (Figure S1B). Overall, exons are strongly depleted in deletions when comparing the overlap in the number of observed and simulated events, while introns show a similar (but much weaker) trend. The frequency of developmental enhancers’ deletion by SVs within natural populations is in between that of exons and introns (Figure S1B), emphasizing their importance in the genome.

Next, we examined whether there was a difference in the prevalence of deletions affecting shadow enhancers compared to non-redundant enhancers associated with mesoderm and/or muscle genes. 151 shadow enhancers are affected by an SV, compared to only 27 non-redundant enhancers, a difference that is borderline significant (odds ratio = 1.47, p = 0.04 from a one-sided Fisher’s exact test), numbers that would certainly increase as more genotypes are sequenced. This result is consistent with the ability of shadow enhancers to buffer against the consequences of genetic perturbations during embryonic development (Figure S1).

The flies harboring these homozygous SVs are alive and viable, at least under laboratory conditions, so even when one of these developmental enhancers is deleted, embryogenesis proceeds largely normally, indicating that the loss of function of this enhancer is compensated by the presence of a second (or third, or fourth) shadow enhancer.

### Predicted Shadow Enhancers Function as Shadow Enhancers In Vivo

To confirm that our predicted shadow enhancers do indeed regulate similar overlapping patterns of expression, we examined the spatiotemporal activity of 23 elements within five loci in vivo using transgenic reporter assays (Supplemental Experimental Procedures). We purposely selected complex regions, where more than a simple pair of shadow enhancers was predicted by at least one method and where naturally occurring SVs removed one or more of the predicted shadow enhancers.

At all five loci, we validated the SV calls by PCR on individual DGRP lines (Figure S2). We then examined the activity of 15 predicted shadow enhancers, as well as eight other enhancers within these loci that were just below the stringent thresholds applied for activity prediction (SVM > 0.95) or correlated TF binding (rho > 0.8). Each of the 23 enhancer elements, which were on average 512 bp in length, were cloned into a common minimal *lacZ* reporter vector and stably integrated into the same location in the *Drosophila* genome using the phiC31 system [43] to allow for a direct comparison of enhancer activities in the same genomic context. The ability of each enhancer to drive spatiotemporal *lacZ* expression was assessed during all stages of embryogenesis by double fluorescence in situ hybridization (FISH) against the *lacZ* reporter and a gene with mesodermal and/or muscle expression.

**Figure 3:**
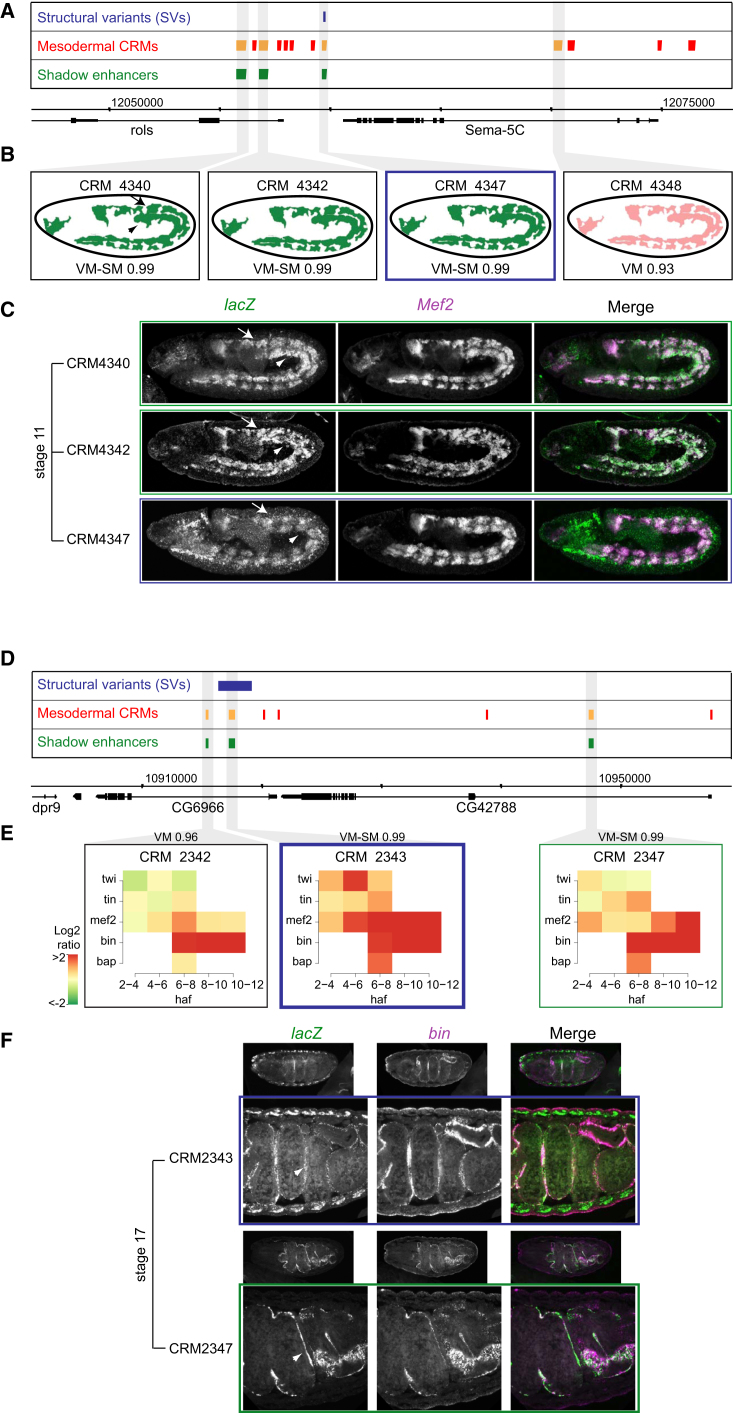
Shadow Enhancers in the *rols* and *CG42788* Loci Predicted shadow enhancers based on similarity in activity (*rols*; A–C) or TF occupancy (*CG42788*; D–F). (A) *rols* locus showing structural variants (blue), mesodermal *cis*-regulatory modules defined by TF-ChIP (CRMs; red), and shadow enhancers (green). (B) Predicted spatial expression of enhancers. Tissue class and SVM score are shown at bottom. VM-SM, visceral muscle-somatic muscle; VM, visceral muscle. (C) Double FISH of transgenic embryos showing *lacZ* reporter (green) under the transcriptional control of three shadow enhancers (CRM4340, CRM4342, and CRM4347) with the pan-mesoderm/muscle marker *Mef2* (magenta). CRM4347 is deleted by an SV (blue, A) and has overlapping expression with CRM4340 and CRM4342 (B and C). SM is indicated by arrows and VM by arrowheads in (D) and (C). A fourth enhancer, CRM4348, which was not predicted to be a shadow enhancer, drives expression in ectodermal strips (data not shown). (D) *CG42788* locus showing structural variants (blue), mesodermal *cis*-regulatory modules (CRMs; red), and shadow enhancers (green). (E) Three shadow enhancers predicted based on both similar activity and highly correlated TF occupancy. The heatmap shows the ChIP peak height signal for each factor/time point. SVM prediction and score are shown above. (F) Double FISH of transgenic embryos showing *lacZ* reporter (green) under the transcriptional control of two shadow enhancers (CRM2343 and CRM2347) with the visceral muscle (VM) marker *biniou* (*bin*) (magenta). CRM2343 is completely deleted by an SV (A) and has overlapping expression with CRM2347. VM is indicated by the white arrowhead. CRM2342 did not share regions of spatial overlap with the other enhancers. Enhancers tested in transgenic embryos are indicated in orange. All embryos oriented with anterior to the left and dorsal at the top. See also Figures S2–S4.

At all five loci, we observed overlapping spatial activity from multiple enhancers, validating the predicted shadow enhancers’ activity in all cases. The *rolling pebbles* (*rols*) and *CG42788* loci both contain multiple enhancers with predicted redundant activity. The *rols* gene codes for an essential protein that forms part of a multiprotein complex essential for myoblast fusion [44, 45]. Loss-of-function mutant embryos fail to hatch due to a severe defect in myoblast fusion and therefore don’t survive beyond embryogenesis [44, 45]. Of the three predicted shadow enhancers examined (Figures 3A and 3B), CRM4347 is deleted by an SV in 11 of the 205 isogenic *Drosophila* lines (Table S4). All three enhancers drive reporter gene expression in overlapping spatial domains at stage 11 and 12 (Figures 3C and S3), despite a clear difference in TF occupancy (data not shown). As predicted by the SVM approach, the three enhancers are active in the visceral and somatic muscle, each partially recapitulating the expression of the endogenous *rols* gene. Similarly, the *CG42788* locus contains three predicted shadow enhancers based on their highly correlated TF occupancy or predicted visceral mesoderm activity (Figures 3D and 3E). Examination of *lacZ* reporter gene expression in transgenic embryos revealed that two of the three enhancers drive overlapping expression in the trunk visceral muscle at stages 15–17 of embryogenesis (Figures 3F and S4).

**Figure 4:**
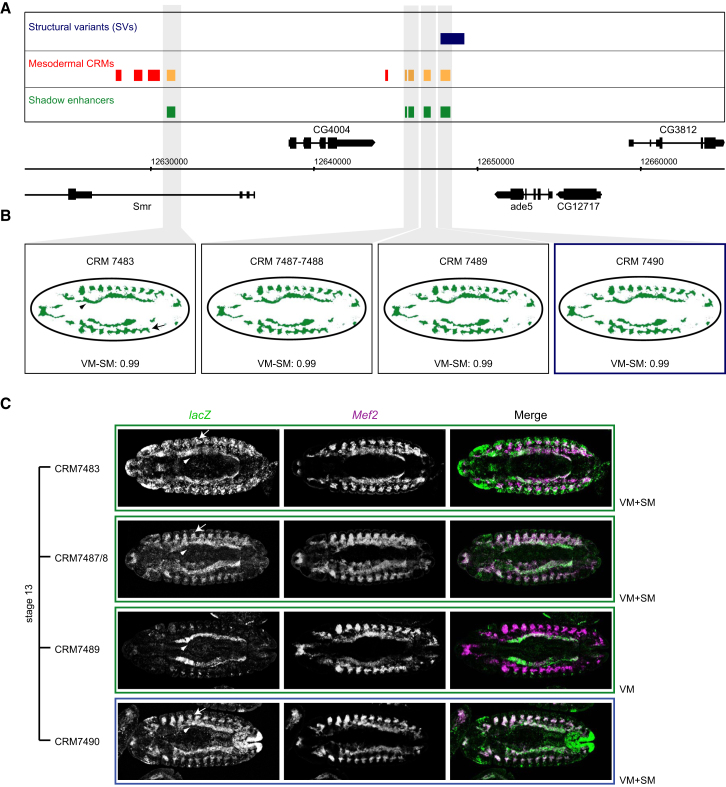
Shadow Enhancers in *ade5* Locus (A) *ade5* locus showing structural variants (blue), mesodermal *cis*-regulatory modules (CRMs; red), shadow enhancers (green). Enhancers tested in transgenic embryos are indicated in orange. (B) Predicted spatial activity of enhancers and SVM scores. VM-SM, visceral muscle-somatic muscle. (C) Double FISH of transgenic embryos showing *lacZ* reporter (green) under the transcriptional control of four shadow enhancers (CRM7483, CRM7487/88, CRM7489, and CRM7490) with the pan-mesoderm/muscle marker *Mef2* (magenta). SM is indicated by arrows and VM by arrowheads in (B) and (C). CRM7490 is almost completely deleted by an SV (blue, A) and has overlapping expression with CRM7483, CRM7487-88, and CRM7489 (green) in VM. More stages are shown in Figure S5. All embryos oriented with anterior to the left and dorsal at the top. See also Figures S2 and S5.

The *ade5* locus contains four shadow enhancers based on their predicted activity. *ade5* regulates de novo purine synthesis and is essential for viability [46]. CRM7490 regulates expression in the somatic and visceral mesoderm from stage 11 to stage 14 of embryogenesis and is completely deleted by an SV in an isogenic line that is viable and fertile (Figure 4). Three other shadow enhancers (CRM7483, CRM7487/8, and CRM7489) regulate overlapping spatiotemporal activity to the deleted enhancer, driving *lacZ* expression either only in the visceral mesoderm or in both the somatic and visceral mesoderm at some or all developmental stages (Figures 4C and S5). This complex locus demonstrates partial redundancy at both a spatial and temporal level, where many enhancers with overlapping expression are likely to be involved in the generation of robust and specific gene expression patterns.

**Figure 5:**
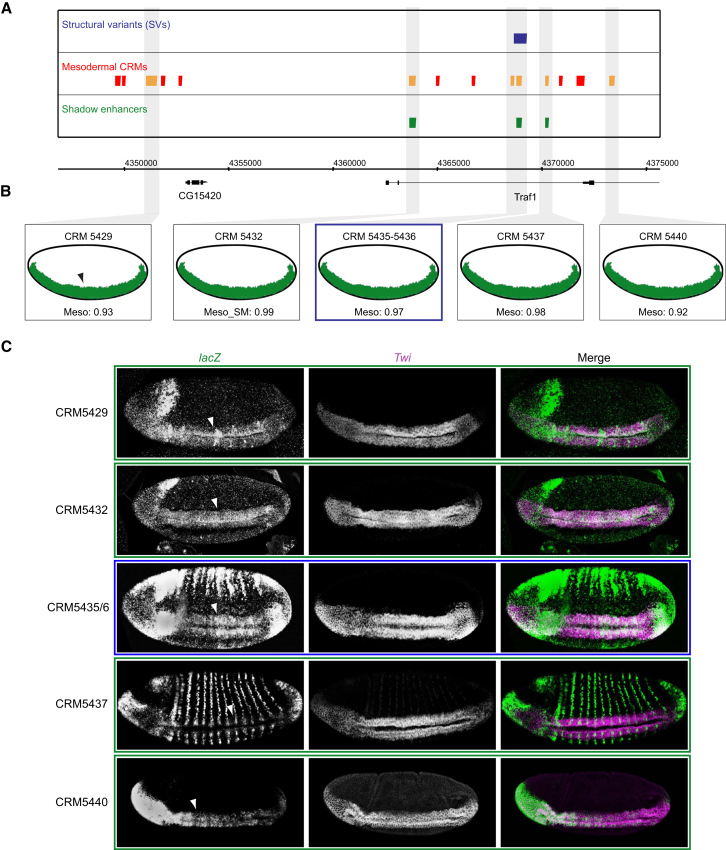
Complexity of *Traf1* Regulation (A) *Traf1* locus showing structural variants (blue), mesodermal *cis*-regulatory modules (CRMs; red), and shadow enhancers (green). Enhancers tested in transgenic embryos are indicated in orange. (B) Predicted spatial activity of enhancers and SVM scores are shown. Meso, mesoderm; Meso-SM, mesoderm and somatic muscle. Three shadow enhancers were predicted (CRM5432, CRM5435/6, and CRM5437) and two additional tested below the applied SVM specificity score (CRM5429 and CRM5440). (C) Double FISH of transgenic embryos showing *lacZ* reporter (green) under the transcriptional control of five shadow enhancers (CRM5429, CRM5432, CRM5435/6, CRM5437, and CRM5440) with the early mesoderm marker *Twist* (*Twi*; magenta). Mesoderm is indicated by arrowheads in (B) and (C). CRM5435/6 is almost completely deleted by an SV (blue, A) and has overlapping expression with CRM5429, CRM5432, and CRM5440 in the mesoderm. All embryos oriented with anterior to the left and dorsal at the top. See also Figures S2 and S5.

Three shadow enhancers within the first intron of the large isoform of *Traf1* are all predicted to be active in the early mesoderm (Figure 5). One of these enhancers is almost completely removed by an SV in two out of 205 individuals. The *Traf1* gene encodes a member of the tumor necrosis factor receptor superfamily. Loss-of-function *Traf1* mutants fail to develop beyond larval stages due to defects in imaginal disc and brain development [47]. We generated transgenic embryos for the three predicted shadow enhancers, as well as two other enhancers within the locus that had predicted mesoderm activity just below our SVM cutoff (<0.95; Figure 5B). Examination of *lacZ* expression revealed that all five enhancers have overlapping activity in the presumptive mesoderm at stage 6 of development, ranging from almost the entire mesoderm (CRM5429, CRM5432, and CRM5435/6) to subsets of mesodermal cells (CRM5437 and CRM5440) (Figure 5C). Therefore, although the total spatial expression pattern of each enhancer varies, they are all active in a population of mesodermal cells at the same stage of development. These results highlight the complexity of *Traf1*’s transcriptional regulation and the extent to which enhancer activity may be buffered by additional elements regulating expression in the same cells at a given stage of development.

**Figure 6:**
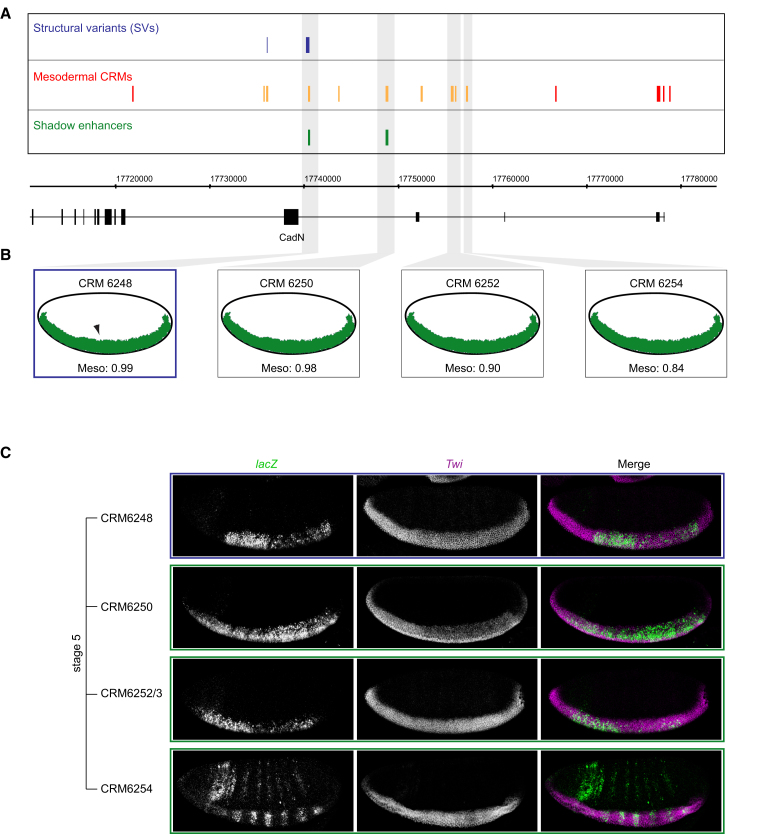
*CadN* Locus Has Many Enhancers with Partially Overlapping Activity (A) *CadN* locus showing structural variants (blue), mesodermal *cis*-regulatory modules (CRMs; red), and shadow enhancers (green). Enhancers tested in transgenic embryos are indicated in orange. (B) Shadow enhancers predicted based on both similarity in activity and correlated TF occupancy. The heatmap shows ChIP peak height signal for each factor/time point. SVM prediction and score are shown above. (C) Double FISH of transgenic embryos showing *lacZ* reporter (green) under the transcriptional control of four enhancers (CRM6248, CRM6250, CRM6252/3, and CRM6254) with the early mesoderm marker *Twist* (*Twi*; magenta). CRM6248 is partially deleted by an SV (blue, A) and has overlapping expression with CRM6250 and two additional CRMs just below the applied cutoff (CRM6252 and CRM6254) in the presumptive mesoderm. Double FISH for *lacZ* (green) and the marker expressed early in the mesoderm Twist (magenta). All embryos oriented with anterior to the left and dorsal at the top. See also Figure S2.

A similarly complex example is the *Cadherin-N* (*CadN*) locus. *CadN* is essential for cell-cell interactions during many process of development, including mesoderm gastrulation and the embryonic nervous system [48, 49]. In this locus, we predicted two shadow enhancers based on their predicted activity (Figure 6), CRM6248 and CRM6250, one of which (CRM6248) is almost completely deleted by an SV in two isogenic lines (Figure 6A). We examined the activity of both enhancers and five additional elements, two of which had similar TF binding signatures but were just below the stringent threshold applied (CRM6252/3 and CRM6254; Figure 6B). Four of the seven enhancers showed highly specific activity in the presumptive mesoderm at stage 5 and have highly overlapping activity in mesodermal cells (Figure 6C). All four enhancers are located within introns of the *CadN* gene and drive expression that is partially overlapping that of the endogenous gene.

Taken together, these data demonstrate that our prediction of shadow enhancers is very accurate, and indicate three clear properties of shadow enhancers. First, they are pervasive throughout the genome. Although this study provides a first systematic attempt to estimate how frequently this occurs, given our conservative thresholds, these predictions are clearly underestimating the number of enhancers with similar, overlapping activity within a given gene’s locus. Second, the level of potential redundancy is much more complex than typically envisaged. In over half of the cases, it is not simply two enhancers that may act redundantly; often there are three (*rols*), four (*CadN* and *ade5*) or even five (*Traf1*) enhancers with overlapping activity. Third, this extensive level of potential *cis*-regulatory redundancy is not only present in the loci of TFs, which are a class of proteins known to have complex transcriptional regulation [50], but is also prevalent in loci for a wide range of essential genes. Here, we purposely chose gene loci of proteins with diverse function; CadN is an adhesion protein, Traf1 a signaling receptor, Rols is an intracellular adaptor protein, Ade5 is a metabolic protein, and CG6966 protein is a predicted component of the E3 ubiquitin-protein ligase complex.

### Conservation of Shadow Enhancers

This high-confidence set represents the largest collection of shadow enhancers in any species to date and thereby enables an initial comprehensive analysis of their general properties. We therefore examined several predictions concerning shadow enhancers: (1) If they are truly redundant, they should show relaxed selective constraints relative to non-redundant enhancers. (2) If shadow enhancers act as substrates for the evolution of regulatory novelty [2] and are pervasive features of gene regulatory networks, they should be frequently associated with lineage-specific signatures of adaptive evolution, compared to non-redundant enhancers.

**Figure 7:**
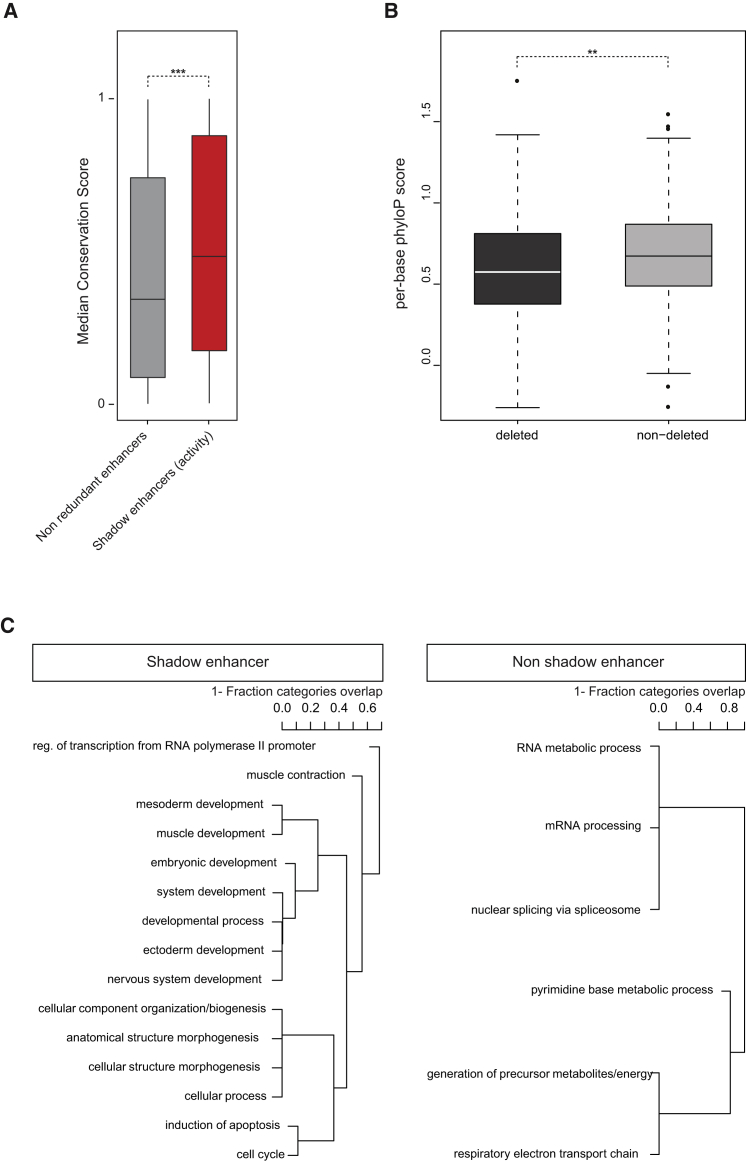
Conservation and Selection of Shadow Enhancers (A) Difference in conservation level (median PhastCons) of shadow versus non-redundant enhancers (Wilcoxon rank-sum test, ^∗∗∗^p < 0.001). (B) Difference in conservation level between shadow enhancers deleted or not by segregating SVs within a natural population (Wilcoxon rank-sum test, ^∗∗^p < 0.01). (C) Biological process enrichment for genes with shadow enhancers; significant terms are shown (based on Fisher’s exact test, p < 0.05). See also Figure S1.

We first investigated whether there is a difference in the overall level of conservation between shadow enhancers and non-redundant enhancers, the latter being defined as enhancers associated with the same mesodermal and/or muscle genes but active in different tissues (i.e., non-shadow enhancers; Supplemental Experimental Procedures). We focused on the shadow enhancers with predicted similar activity (SVM), given the much larger size of this collection (866 elements). In contrast to what is expected for redundant elements, PhastCons (median 15-way genome conservation scores [51]) and phyloP scores indicate that shadow enhancers are more conserved than non-redundant enhancers (p = 0.00079 and p = 0.00048 for PhastCons and phyloP, respectively; Wilcoxon rank-sum test; Figure 7A). The difference in evolutionary rates is modest (2.85 substitutions per base over the 12 species phylogeny versus 2.557), but it translates to an ∼10% difference in the number of substitutions, suggesting that shadow enhancers, although acting redundantly in one context, are most likely acting non-redundantly in another context or function. Interestingly, despite this overall higher level of conservation, the subset of shadow enhancers that are deleted by SVs tend to less conserved in comparison to “shadow pairs” not affected by segregating deletions (p = 0.001719, Wilcoxon rank-sum test; Figure 7B), suggesting that there may be different types of shadow enhancers evolving at different rates.

To more directly assess recent selective pressures influencing shadow enhancer evolution, we examined patterns of segregating genetic variation within 205 isogenic, wild-derived lines as part of the DGRP [40]. Consistent with the actions of directional selection, Tajima’s D statistic, a measure of departure from the site-frequency spectrum expected under neutral evolution [52], was significantly more negative for all mesoderm and/or muscle enhancers compared to flanking, proxy-neutral regions (on average −0.52 versus −0.38, p < 0.00001, Wilcoxon rank-sum test). Tajima’s D values, however, do not differ significantly when these elements were separated into shadow enhancers and non-redundant enhancers or among our sets of proxy neutral regions. If selective pressures were relaxed on a subset of shadow enhancers, we would expect Tajima’s D scores to be more heterogeneous. However, we saw no evidence to support this trend using Tajima’s D or multiple alternative summary statistics (Supplemental Experimental Procedures). In short, we see little evidence from summary statistics indicating that shadow and non-redundant enhancers are evolving under different selective regimes.

We next estimated the fitness consequences of mutations within shadow enhancers and non-redundant enhancers using INSIGHT [53], a probabilistic model that partitions mutations in putative regulatory sites into coarse-grained fitness categories (neutral, weak negative, strong negative, or positive selection). In addition, INSIGHT also infers the fraction of sites with selective effects within categories of sites, a term that can be interpreted as the probability that a mutation in a given region will impact fitness (fitCons score [54]). As this measure is based on current patterns of segregating variation, it is expected to be robust to turnovers in functional sites that may occur between species. In addition to our mesodermal enhancers, we also applied INSIGHT to a large set of genomic regions that function as developmental enhancers in transgenic assays and overlap DNase-hypersensitive sites [32], as well as a set of negative regions that showed no evidence of functioning as enhancers in vivo [32]. Finally, we contrasted this with a set of 100,000 randomly chosen proxy-neutral sites that lie outside of phastCons blocks, known exons, peaks of H3K4me1, or DNase-hypersensitive sites.

For all sites, we saw a significant elevation in fitCons score relative to proxy-neutral sites, with an estimated 46%–65% of sites having a potential impact on fitness, in line with previous estimates of constraint in *Drosophila* non-coding DNA (e.g., 49.3% estimated by P. Andolfatto [55]). As expected, this fraction was lowest for genomic regions that do not function as embryonic enhancers, although still significantly higher than inferred for most classes of human regulatory DNA [54], reflecting the more compact *Drosophila* genome. Among all enhancers examined, the fitCons score was lowest for non-redundant enhancers (0.54 versus 0.59 for shadow enhancers), suggesting a higher average fitness consequence for mutations occurring in shadow enhancers. Interestingly, this difference does not reflect differences in TF occupancy for these regions, with approximately equal average occupancy for shadow and non-redundant enhancers (mean TF binding 7.26 versus 8.10). Together, these results suggest that despite similar properties of TF occupancy, mutations in shadow enhancers are slightly more likely to impact fitness than are mutations in non-redundant enhancers.

Shadow enhancers have been suggested to serve as substrate for the evolution of regulatory novelty [2]. Although challenging to test in full, if shadow enhancers frequently serve as substrate for adaptive evolution, their DNA sequences should show an enrichment of (lineage-specific) signatures for positive selection. As our INSIGHT analyses did not identify a meaningful number of positively selected sites in any enhancer set, we sought additional information in between-species substitution rates. Specifically, we tested for lineage-specific accelerated evolution at these enhancers by contrasting patterns of nucleotide evolution within regions with neutral evolution (inferred from 4-fold degenerate codon positions [4d sites]) using a phylogenetically aware likelihood framework [56]. Consistent with pervasive negative selection, few regions (0.2%–1.5%) showed evidence for phylogeny-wide acceleration relative to 4d sites, with no significant differences in proportion among shadow versus non-redundant enhancers. A higher fraction of non-redundant enhancers, however, showed evidence (p < 0.05, likelihood ratio test) for lineage-specific acceleration (relative to subtree evolutionary rates) along the *D. melanogaster* lineage (8.2% and 11.1% for shadow and non-redundant, respectively), though the contrast between shadow and non-redundant enhancers is not statistically significant. Although the significance threshold for accelerated evolution applied here (p = 0.05) does not provide strong evidence for pervasive adaptation, the results do suggest that signatures for adaptive change are marginally more common among non-redundant enhancers than shadow enhancers.

To explore the reason for this difference in conservation, we assessed Gene Ontology (GO) enrichment among genes associated with shadow enhancers compared to non-redundant enhancers (defined as genes with enhancers with non-overlapping expression in any tissue). The six most highly enriched gene sets are all related to development (Figure 7C; Table S2). Given the limited background (∼600 genes with one or more non-redundant enhancers), these results are not significant after multiple testing. However, we note that as the significant categories are highly overlapping (Figure 7C), multiple testing corrections may be overly harsh in this context. We thus present all nominally significant categories (Table S6), which are highly consistent with previous studies [57]. Conversely, genes associated with non-redundant enhancers (i.e., enhancers within a gene’s locus that regulate different patterns of expression) tend to be enriched for housekeeping function (Table S3); thus, despite the fact that both sets of genes are active in the developing mesoderm and derivatives, only genes associated with shadow enhancers are enriched for developmental terms. This suggests that the higher level of conservation observed for shadow enhancers (Figure 7A) may be partly explained by their association with key developmental genes, known to have more complex and conserved regulatory landscapes.

Together, these results suggest that while shadow enhancers may, in some cases, compensate for mutations affecting their partner, they are not redundant in the strict definition—contrary to expectation for elements with absolute redundancy, shadow enhancers are maintained by selection to the same, or an even greater degree, than non-redundant enhancers and show no evidence for lineage-specific adaptation, suggesting that they may have essential functions in their own right.

## Discussion

The presence and function of redundant elements in the regulation of the gene expression has been discussed with interest over the past two decades. In the context of embryonic development, there are a number of examples where two enhancers act in an apparently redundant manner to regulate the expression of well characterized gene loci [1, 4, 6, 7, 8, 9, 58]. Recent work suggests that these shadow enhancers play an important role in providing spatial precision, temporal synchrony, and generating generalized robustness to gene expression, thus uncovering more complex regulatory functions [3, 6, 7, 26, 27]. However, the frequency of redundant enhancers and the types of evolutionary forces that shape them remain poorly understood. Here we present a systematic genome-wide assessment of the extent and complexity of shadow enhancers within gene loci during embryonic development. Our results reveal that shadow enhancers (i.e., elements with similar overlapping spatial activity) are a fundamental component of developmental genes’ regulatory landscape and go far beyond a simple “two redundant enhancer” model. Interestingly, we find that the selective constraint on these shadow enhancers appears to be as great as or greater than that for non-redundant enhancers, highlighting the fact that although shadow enhancers may have an important role in buffering development, they are far from dispensable.

### Shadow Enhancers Are Pervasive throughout the Genome

This systematic assessment of the extent of redundant enhancer activity indicates that shadow enhancers are much more common, and in more complex relationships, than currently envisaged. Based on our stringent criteria, we identified 1,055 shadow enhancers. However, our extensive in vivo analysis indicates that this is almost certainly an underestimate of the extent of redundancy: when we tested the activity of regions just below the cutoffs used to define shadow enhancers, for example in the loci of *CadN*, *rols*, and *Traf1* genes, we observed that many additional elements also have similar spatial activity. This is supported by a previous study examining the occupancy of the TF Dorsal, which estimated that one-third of its target genes may contain a redundant enhancer [37]. That study and ours focused on enhancers bound by a small repertoire of TFs: extrapolating to all ∼700 or so predicted *Drosophila* TFs suggests that shadow enhancers are prevalent throughout the genome and therefore could have a substantial impact on the robustness of gene expression during embryonic development. As we discuss below, however, this largely hidden layer is not without primary function, but rather may play a fundamental role in ensuring the precision, timing, and robustness of specific developmental programs, as has recently been shown at individual gene loci [3, 7, 26]. Just as promoter variants that lead to transcriptional noise are suppressed within natural populations, as seen in yeast [29], shadow enhancers may play a crucial role in the suppression of transcriptional noise during embryonic development.

### How Are Redundant Enhancers Maintained during Evolution?

The partially overlapping activity of redundant enhancers appears to be an emerging theme, but one with an evolutionary paradox. In agreement with the strict definition of redundancy, deletion of a redundant enhancer does not cause major phenotypic alteration, at least in a given environmental condition, as one or more redundant elements could compensate for the loss. What then prevents the deletion of shadow enhancers with a population?

The answer may lie in the context specific nature of their redundancy, which we are referring to here as partial redundancy. As these elements drive overlapping patterns of expression, there are at least some tissues, stages, or environmental conditions in which the elements have distinct functional roles. The overlap in activity (similar expression pattern) can be restricted to a small time window or a small number of cells, while other shadow enhancer “pairs” may have extensive overlap in time or space (Figures 4 and 5). Thus, although an enhancer may be redundant with another element in one tissue or developmental stage, its activity may be non-redundant in another cell type and therefore be essential for embryonic development. Similarly, enhancers that appear redundant in “normal” environmental conditions could act non-redundantly when the environmental conditions become more extreme, as observed in the *svb* locus [3]. It is this partially redundant property that most likely holds the key to how these elements are maintained over long evolutionary periods.

A previous study hypothesized that there may be different evolutionary pressure on two redundant enhancers: the primary enhancer being more constrained than the redundant shadow enhancer, allowing the later to accumulate mutations without inducing a phenotype and thus evolve faster [2]. Our analyses of sequence conservation and the frequency of segregating mutations affecting these enhancers doesn’t support this, at least in the context of these mesoderm/muscle enhancers; the evolutionary pressures affecting shadow enhancers are similar and overall show a stronger tendency toward conservation than non-redundant enhancers driving similar expression with no evidence for an increased frequency of relaxed selection or adaptive evolution, although we appreciate that these approaches are most likely underpowered to detect recent adaptive changes. Taken together, our results suggest that shadow enhancers are being maintained for a purpose. One property of many shadow enhancers, in addition to their similar overlapping activity, is that the majority also have additional non-redundant activity, which may be under selective pressure, as discussed above. Alternatively, “redundant” enhancers driving similar spatiotemporal activity could act together to guarantee that a gene reaches a certain level of expression [28], or could have essential roles in ensuring correct patterning precision [5, 26], or to reduce stochastic effects on gene expression [7], and thereby play an essential role in reducing transcription noise during development. In these cases, shadow enhancers ensure robustness of the trait when environmental variations occur but do not confer genetic robustness to all possible mutations since, for example, deletions of a partially redundant enhancer can drastically influence the viability of an organism [26].

We therefore argue that shadow enhancers are pervasive throughout the genome and provide robustness to gene expression in the context of fluctuating genetic and environmental perturbations. The redundant function of these enhancers, e.g., similar overlapping expression, may provide opportunities for evolutionary innovation; however, the non-redundant part of the enhancer’s activity, e.g., in space, time, or environmental conditions, indicates that they also have independent functional roles, which may help to fix these elements within a population.

In summary, the data presented here indicate that almost any developmental gene can have multiple shadow enhancers, each with similar overlapping windows of activity. The combined action of partially redundant enhancers may thereby represent a significant strategy through which an organism reaches robustness during embryonic development. The extensive nature of the overlap of these elements activity will generate distributed robustness within large developmental gene regulatory networks, a role that has yet to be explored. Their prevalence may give insights into how gene regulatory networks are organized—with the modular nature of enhancers (i.e., the building blocks of gene regulatory architecture) required to produce robust and precise patterns perhaps providing redundancy (mutational robustness) as a useful byproduct.

## Author Contributions

E.C. and E.E.M.F. designed the study and analyzed the results. E.C., E.H.G., and L.C. generated all transgenic lines and performed in situ hybridization and imaging. P.K. correlated TF occupancy. D.G. and P.G. performed conservation, GO enrichment, and expression similarity analysis. T.Z. and J.O.K. did SV analysis. E.C., D.G., and E.E.M.F prepared and edited the manuscript.

## Supplemental Information

Document S1. Supplemental Experimental Procedures and Figures S1–S5

Table S1. List of all Shadow Enhancers with their Associated Genes

Table S2. List of GO Terms Enriched in Genes Regulated by Shadow Enhancers

Table S3. List of GO Terms Enriched in Genes Regulated by Non-Shadow Enhancers

Table S4. List of Structural Variants in DGRP Lines

Table S5. List of Used Primers for SV Validation

Table S6. List of GO Biological Processes Enriched for Genes Regulated by Shadow Enhancers

Document S2. Article plus Supplemental Information
