# Supplementary material for: Shadow Enhancers Are Pervasive Features of Developmental Regulatory Networks
Source: Curr Biol. 2016 Jan 11;26(1):38–51. doi: 10.1016/j.cub.2015.11.034 (PMC4712172; doi:10.1016/j.cub.2015.11.034)
Supplement: Document S1. Supplemental Experimental Procedures and Figures S1–S5 [file mmc1.pdf]

Current Biology

Supplemental Information

Shadow Enhancers Are Pervasive Features of Developmental Regulatory Networks

Enrico Cannavò, Pierre Khoueiry, David A. Garfield, Paul Geeleher, Thomas Zichner, E. Hilary Gustafson, Lucia Ciglar, Jan O. Korbel, and Eileen E.M. Furlong

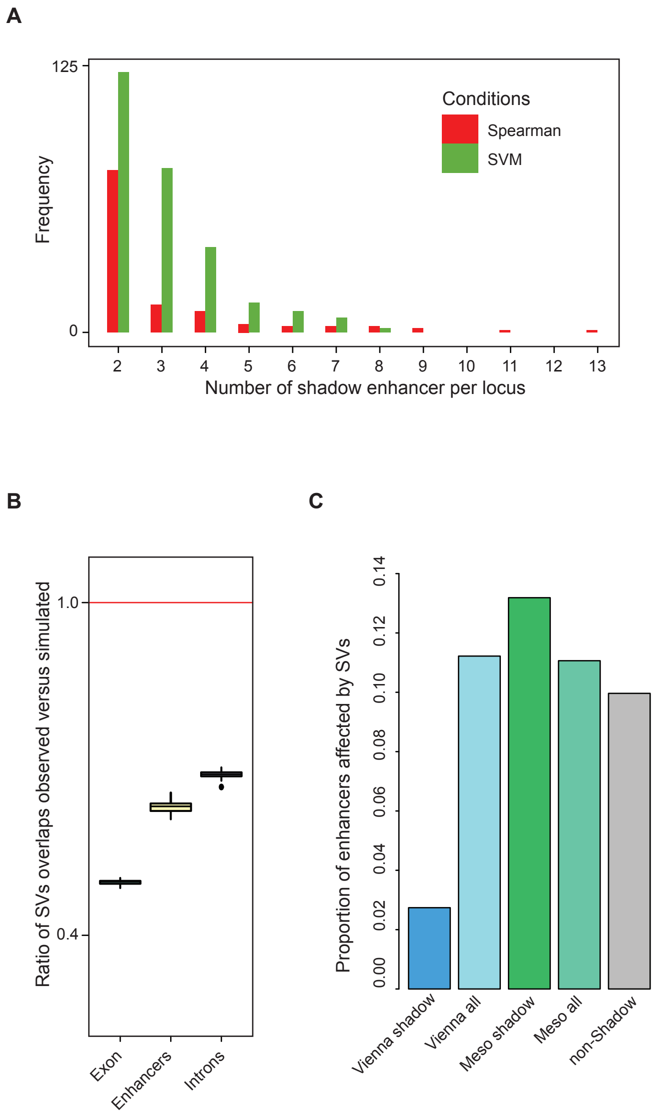

Figure S1. General properties of shadow enhancers (related to Figure 7)

A) Number of shadow enhancers (x-axis) regulating different number of genes (y-axis), identified by the SVM (blue; similarity in activity) or Spearman's correlation (red, similarity in TF occupancy). **B)** Ratio of naturally occurring SVs that overlap exons, introns and enhancers (set to one, red horizontal line), compared to the number observed when SVs are shifted randomly in +/- 50kb windows around their actual location. **C)** Proportion of deleted enhancers among various sets: shadow enhancers in the Vienna tiles versus all active Vienna enhancers (blue), mesodermal shadow enhancers (Meso, green) versus all mesodermal enhancers (green) and all non-redundant enhancers (grey).

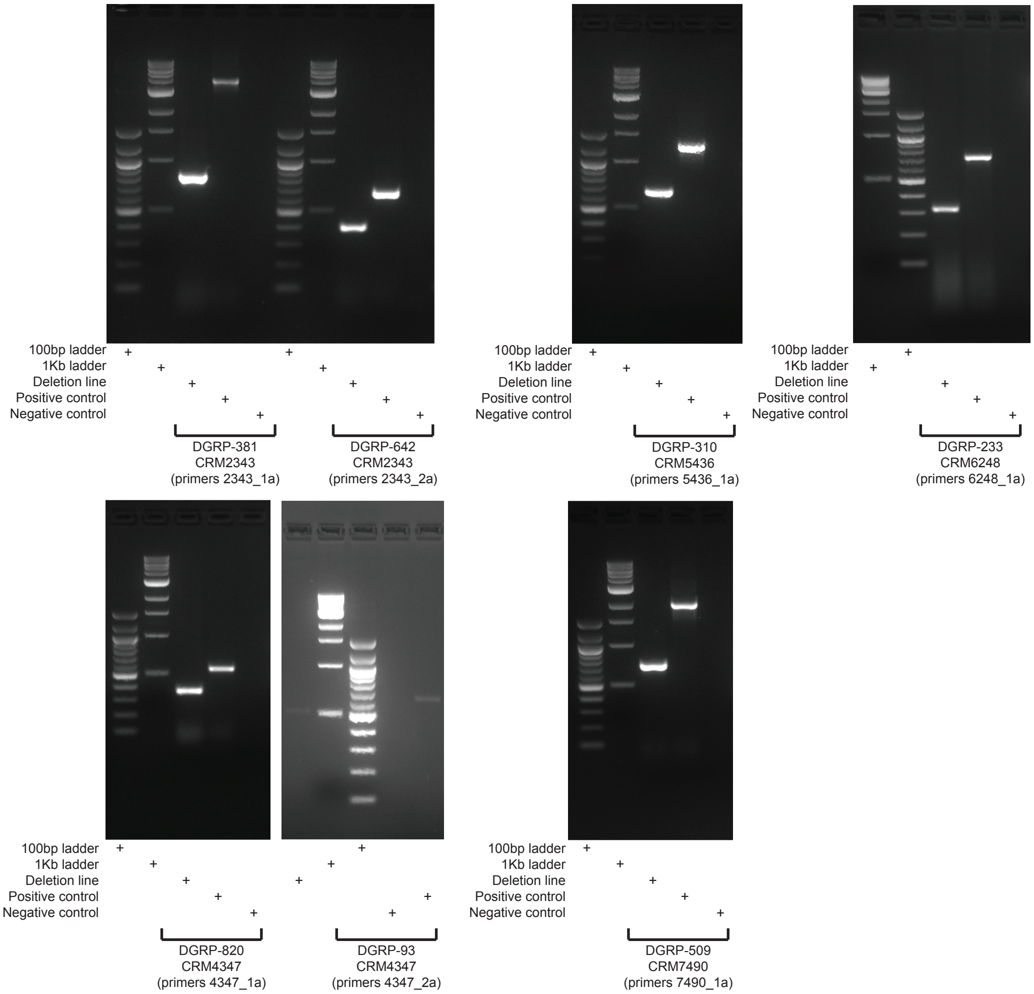

Figure S2. Validation of enhancer SV deletion by PCR (related to Figures 3-6)

We used the indicate primers (Table S5) to validate the predicted deletion of enhancers in one or two DGRP lines. DNA extracted from the reference line 2057 (Bloomington Stock Number) was used as positive control.

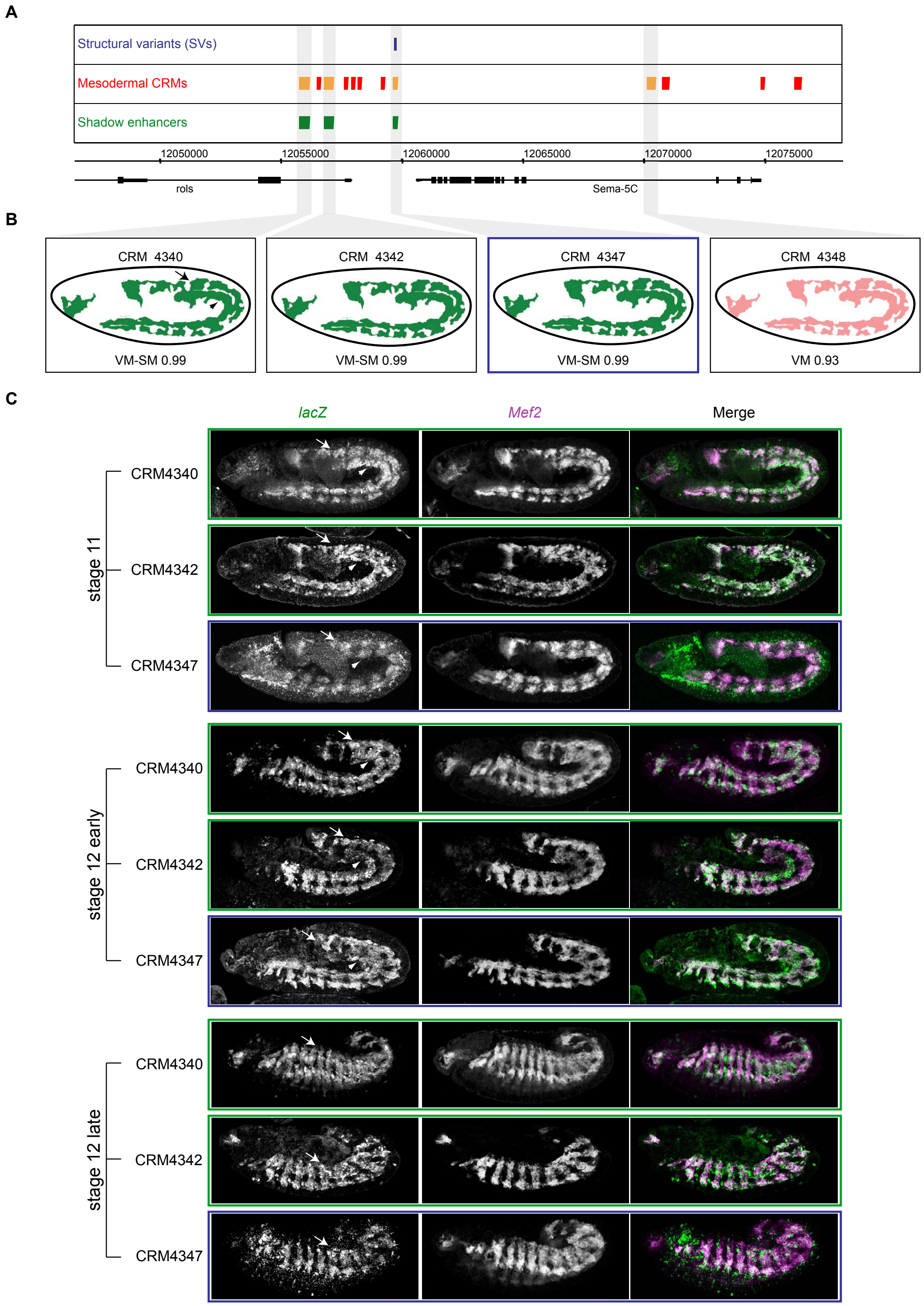
A
Structural variants (SVs)
Mesodermal CRMs
Shadow enhancers
12050000
12055000
12060000
12065000
12070000
12075000
rols
Sema-5C
B
CRM 4340
VM-SM 0.99
CRM 4342
VM-SM 0.99
CRM 4347
VM-SM 0.99
CRM 4348
VM 0.93
C
lacZ
Mef2
Merge
stage 11
CRM4340
CRM4342
CRM4347
stage 12 early
CRM4340
CRM4342
CRM4347
stage 12 late
CRM4340
CRM4342
CRM4347

Figure S3. **Shadow enhancers in the *rols* locus (related to Figure 3)**

A) *rols* locus showing structural variants (blue), mesodermal *cis*-regulatory modules defined by TF-ChIP (CRMs, red), shadow enhancers (green). Enhancers tested in transgenic embryos indicated in orange. **B)** Predicted spatial expression of enhancers. Tissue class and SVM score shown at bottom: Visceral muscle-somatic muscle (VM-SM) and Visceral muscle (VM). **C)** Double FISH of transgenic embryos showing *lacZ* reporter (green) under the transcriptional control of three shadow enhancers (CRM4340, 4342, 4347) with pan-mesoderm/muscle marker, *Mef2* (magenta). SM indicated by arrow and VM by arrowhead in B,C. CRM4347 is deleted by an SV (blue (A)) and has overlapping expression with CRM4340 and CRM4342 (B,C). Shown are embryonic stages 11 to late stage 12.

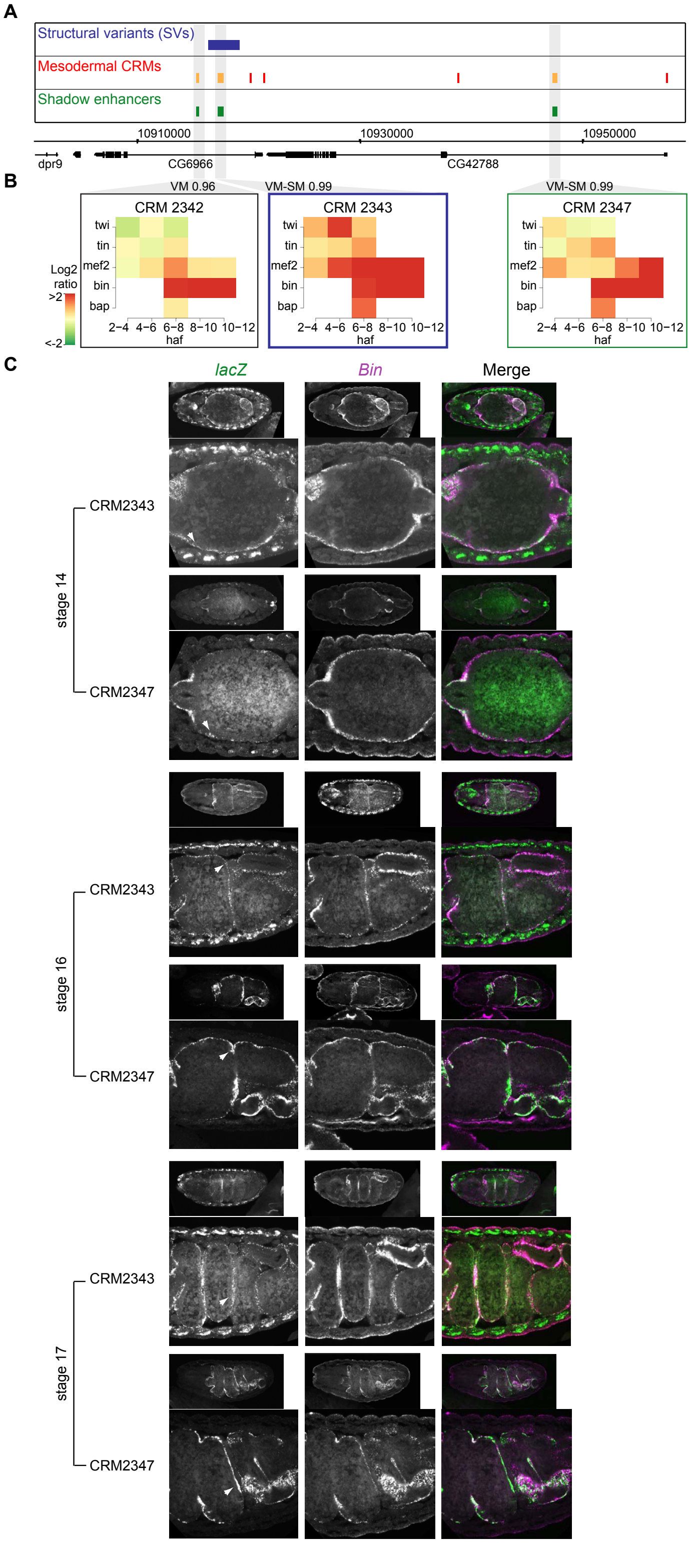
A
Structural variants (SVs)
Mesodermal CRMs
Shadow enhancers
10910000
10930000
10950000
dpr9
CG6966
CG42788
B
VM 0.96
VM-SM 0.99
VM-SM 0.99
CRM 2342
CRM 2343
CRM 2347
twi
tin
mef2
bin
bap
2–4 4–6 6–8 8–10 10–12
haf
Log2 ratio
>2
<-2
C
lacZ
Bin
Merge
CRM2343
CRM2347
stage 14
CRM2343
CRM2347
stage 16
CRM2343
CRM2347
stage 17

Figure S4. **Shadow enhancers in the *CG42788* locus (related to Figure 3)**

A) *CG42788* locus showing structural variants (blue), mesodermal *cis*-regulatory modules (CRMs, red), shadow enhancers (green). Enhancers tested in transgenic embryos indicated in orange. **B)** Three shadow enhancers predicted based on highly correlated TF occupancy, heat map shows ChIP peak height signal for each factor/timepoint. SVM prediction and score is shown above. **C)** Double FISH of transgenic embryos showing *lacZ* reporter (green) under the transcriptional control of two shadow enhancers (CRM2343, CRM2347) with the visceral muscle (VM) marker, *biniou (bin)* (magenta). VM indicated by white arrowhead. CRM2343 is completely deleted by an SV (A) and has overlapping expression with CRM2347. CRM2342 did not share regions of overlap with the other shadow enhancers. Enhancers tested in transgenic embryos indicated in orange. Embryo orientation: anterior-left, dorsal-up,

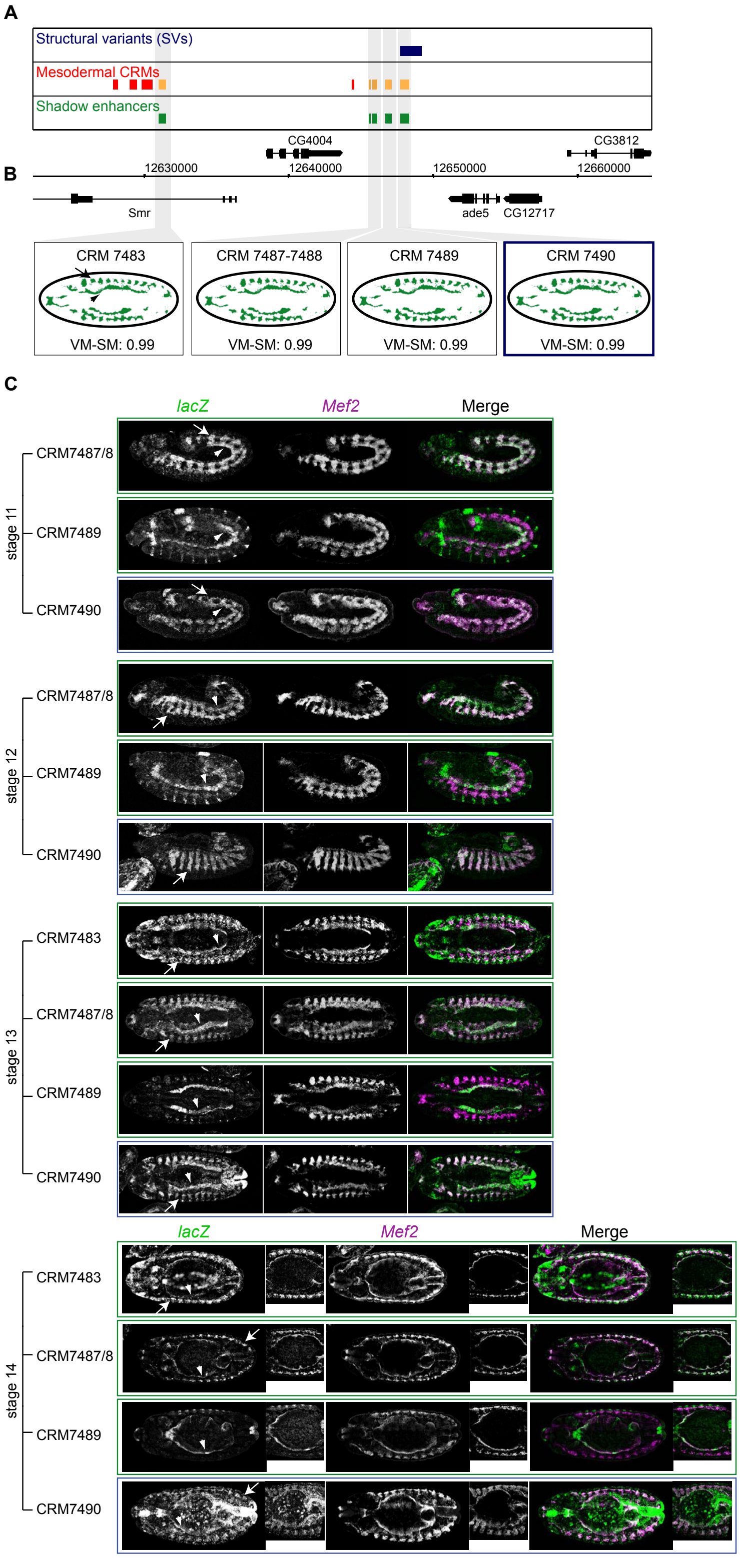
A
Structural variants (SVs)
Mesodermal CRMs
Shadow enhancers
CG4004
CG3812
B
12630000
12640000
12650000
12660000
Smr
ade5
CG12717
CRM 7483
CRM 7487-7488
CRM 7489
CRM 7490
VM-SM: 0.99
VM-SM: 0.99
VM-SM: 0.99
VM-SM: 0.99
C
lacZ
Mef2
Merge
stage 11
CRM7487/8
CRM7489
CRM7490
stage 12
CRM7487/8
CRM7489
CRM7490
stage 13
CRM7483
CRM7487/8
CRM7489
CRM7490
lacZ
Mef2
Merge
stage 14
CRM7483
CRM7487/8
CRM7489
CRM7490

Figure S5. Shadow enhancers in *ade5* locus (related to Figure 4)

A) *ade5* locus showing structural variants (blue), mesodermal *cis*-regulatory modules (CRMs, red), shadow enhancers (green). Enhancers tested in transgenic embryos indicated in orange. **B)** Predicted spatial activity of enhancers and SVM scores are shown: Visceral muscle-somatic muscle (VM-SM). **C)** Double FISH of transgenic embryos showing *lacZ* reporter (green) under the transcriptional control of four shadow enhancers (CRM7483, 7487/88, 7489, 7490) with pan-mesoderm/muscle marker, *Mef2* (magenta). CRM7490 is almost completely deleted by an SV (blue (A)) and has overlapping expression with CRM7483, CRM7487-88 and CRM7489 (green) in VM. Specifically, CRM7490, CRM7487/8 (from stage 11 to stage 14) and CRM 7483 (stage 13 and 14) show activity in somatic and visceral mesoderm while CRM 7489 is active only in the visceral mesoderm (from stage 11 to stage 14). SM indicated by arrow and VM by arrowhead in B,C. All embryos oriented anterior-left, dorsal-up.

Supplemental Tables (available online)

Table S1. Lists all shadow enhancers with their associated gene

Table S2. Lists of GO terms enriched in genes regulated by shadow enhancers

Table S3. Lists of GO terms enriched in genes regulated by non-shadow enhancers

Table S4. List of structural variants in DGRP lines

Table S5. List of used primers for SV validation

Table S6. List of GO biological processes enriched for genes regulated by shadow enhancers

Supplemental Experimental Procedures

Frequency of enhancers with redundant versus partially redundant activity

The spatial activity of 3,604 developmental enhancers examined *in vivo* was obtained from Kvon *et al*. [S1]. Each enhancer was assigned a score of '0' (inactive), or '1' (active; an intensity of ≥ 2) based on the authors annotation across each of the 227 terms at each of 6 developmental time-points (yielding an "activity vector" of 1,362 scores for each enhancer). Two enhancers were classified as exhibiting "identical" activity if their activity vectors were exactly equivalent, i.e. if both enhancers had exactly the same activity across all tissue terms and time-points. This identified 10 pairs of enhancers with both identical activity and proximity within 50kb of each other. To assess whether this was more than expected by chance, we constructed a null distribution using a permutation-based assay. For each of 1,000 permutations, the location of all enhancers was shuffled, while only swapping enhancers with similar complexity in their activity (i.e. having the same number of spatio-temporal terms), to maintain the data structure. A *P*-value was estimated based on the proportion of permutations for which the number of pairs of enhancers (within 50kb) with identical activity was less than the number observed.

Enhancers with similar activity (i.e. one or more tissue expression terms is the same, at the same stages of development, as annotated by Kvon et al [S1]) were quantified using the Euclidian distance between their corresponding "activity vectors", based on their *in vivo* activity as annotated by the authors. The global level of similarity between enhancers within 50kb of each other was quantified by the mean Euclidian distance. As above, we used a permutation-based assay to assess whether this observed number was greater or less than expected by chance. In this case, we also performed 1,000 permutations, but because the observed distance was far

smaller than those observed by random chance, the *P*-value reported was calculated from a theoretical normal distribution, the parameters of which were estimated using the 1,000 values obtained from the permutations. For gene expression, the same methods described above were applied, except the spatio-temporal activity vector for the enhancers was replaced by expression data for genes obtained from the Berkeley *Drosophila* Genome Project in-situ hybridization database.

Genome-wide identification of shadow enhancers

Shadow enhancers were predicted by two methods. The first was based on correlated TF occupancy, using 8,008 ChIP-defined *cis*-regulatory modules (CRMs) [S2]. The mean ChIP intensity profiles for each TF was calculated in a 200 bp window, with a 35 bp step [S2]. This gave a vector of 15 data points for each CRM, which was used to calculate the Spearman rank correlation coefficient for all pairs of CRMs in each chromosome. Pairs with Spearman's rho >= 0.8 within a window between 200 bp to 50kb were considered as highly similar. As these TFs are exclusively expressed in mesoderm and/or muscle tissues, we restricted our analysis to highly similar enhancer pairs associated with genes that are expressed in the same tissue (using *in-situ* hybridization data from BDGP and literature annotation from FlyBase, excluding all ubiquitous genes), as described previously [S1]. This identified 350 shadow enhancers (Table S1).

The second method, based on enhancers with similar activity, took advantage of previous tissue predictions using a machine learning approach (a Support Vector Machine (SVM) [S2]. To predict redundant enhancers, we selected pairs within a 200bp to 50 kb window with a high SVM specificity (sp>=0.95) in at least one overlapping tissue class. Enhancers were classified as having similar overlapping activity in one or combinations of 3 tissue types: (1) Mesoderm

was based on enhancers with SVM predictions in ‘Meso_only’ and ‘Meso_SM’, (2) visceral mesoderm (VM), based on SVM predictions for ‘VM_only’ and ‘VM_SM’, (3) somatic mesoderm (SM), based on SVM predictions for ‘Meso_SM’ and ‘VM_SM’, (4) mesoderm and somatic Mesoderm (Meso_SM), based on SVM predictions for ‘Meso_SM’, ‘Meso_only’ and ‘VM_SM’, (5) visceral mesoderm and somatic mesoderm (VM_SM), based on SVM predictions for ‘VM_SM’, ‘Meso_SM’ and ‘VM’. The ‘SM_only’ group was excluded as the SVM predictions were poor upon validation [S2]. The final set of shadow enhancers were those where each enhancer in a pair has predicted activity in the same tissue and is associated with a common gene that is also expressed in that tissue (using *in-situ* hybridization data from BDGP and literature annotation from FlyBase, excluding all ubiquitous genes), as described previously [S1].

This identified a stringent set of 866 shadow enhancers, associated with 298 genes. Shadow enhancers from the both approaches (Spearman and SVM based) were merged to obtain a unique set of 1125 (866 + 350 – 91 common pairs) putative shadow enhancers (Table S1). Although not used for further analysis, we also applied the same criteria to the 3604 Vienna tiles that gave activity, which identified 75 regions that classify as shadow enhances based on their overlapping expression and association to a common target gene. Of these, 2 have SVs within the DGRP collection that removes the enhancer.

Structural Variant detection

To facilitate this study we extended our previous structural variant (SV) analysis from 40 lines [S3] to 205 lines with a few changes. The variant discovery was performed as follows: We inferred deletions in all 205 lines using the four different computational tools Pindel [S4], DELLY [S5], Genome STRiP [S6], and CNVnator [S7]. Genome STRiP [S6] v1.0.4 was used

to perform simultaneous population-scale deletion discovery on the 205 DGRP samples. The minimum required mapping quality for Genome STRiP was set to 20. For Pindel (v0.2.4d) we set the maximum detectable SV size to 129,472 (parameter '-x 6') and the minimum number of matched bases to 20 ('-d 20'). For CNVnator (v0.2.2) we used a bin size of 200 bp. We then integrated the results by merging the individual variant predictions for our four methods and the published freeze2 DGRP set [S3, S8], as described previously [S3], generating a single variant list (Table S4). Based on our previous whole-genome tiling array data for six lines [S3], we estimated the false discovery rate of the final set to be ~ 15%; the fraction of true positive and potential false positive variants was determined by estimating a Gaussian mixture model. Variants where more than 90% of the bases overlap annotated repeats, based on RepeatMasker and TandemRepeatFinder predictions (both downloaded from UCSC on October 15, 2013), were removed.

Conservation analysis

Phastcons scores were obtained using the twelve *Drosophila* species, mosquito, honeybee and red flour beetle (PhastCons 15-way) [S9]. The sources were downloaded from UCSC Genome browser via ftp://hgdownload.cse.ucsc.edu/goldenPath/dm3/phastCons15way/). Non-redundant enhancers (356 elements) were defined as enhancers mapped to the same mesoderm/muscle genes, but driving expression in different tissues, so non-overlapping patterns of expression (and therefore not in a shadow pair). The average conservation scores were compared between shadow enhancers and non-redundant enhancers using a Wilcoxon rank-sum test.

PhyloP scores and expected numbers of substitutions were calculated for each base in the 15-species alignment using the LRT method (for scores) and SPH method (for substitutions) as

implemented in phyloP [S10]. Following Pollard *et al*., we used positive scores to indicate conservation, and negative scores to indicate acceleration relative to a neutral model. Neutral models were generated separately for each chromosome arm using four-fold degenerate codon positions to fit a strand-symmetric general reversible process model (REV) using the program phyloFit [S11]. As the base composition of 4d sites differ rather dramatically from the general base-composition of the non-coding genome, we adjusted our neutral models to reflect the G+C content of the non-coding genome on each chromosome arm, as recommended by Pollard *et. al* [S10]. BigWig file versions of the phyloP results are available upon request.

To estimate more current selective forces acting on these enhancers, we made use of Tajima's D statistics. For this analysis, we divided the 8008 ChIP-CRMs into three groups: the two sets of shadow enhancers (for a total of 1125) and a set of non-redundant enhancers (356) as described above (conservation analysis). For each element we also defined proxy neutral regions consisting of 500bp on either side of the element followed by the removal of any bases found within DNase hypersensitive sites [S3], transcribed genes/ncRNAs (FlyBase release 5.57), or peaks of H3K4me1. For each element, and its flanking neutral proxies, Tajima's D was calculated using custom Python scripts [S12], and the resulting distributions were compared. With these same scripts, we also calculated Fu and Li's D, Fu and Li's F, Fay and Wu's H, as well as HKA statistics (using flanking sequence as locus 2) using *D. simulans* and *D. erecta* as out-groups with qualitatively similar (i.e. non-significant) results.

Although useful for broad views, summary statistics are limited in their ability to adjust for differences in local mutation rates and in their incorporation of information from different parts of the site-frequency spectrum (*e.g.* the contribution of weak negative selection to rare segregating mutations). We thus turned to a probabilistic model, INSIGHT [S13], which

partitions putative regulatory sites into coarse-grained fitness categories (neutral, weak negative, strong negative, or positive selection) using patterns of polymorphism and divergence between these sites and flanking, neutral sequence. The model also infers the fraction of sites with selective effects, a term that can be interpreted as the probability (fitCons score, [S14]) that a mutation will impact fitness.

As sequencing errors in closely related out groups can strongly influence both INSIGHT and our lineage-specific tests for selection, we constructed a custom 12-way *Drosophila* alignment in which *D. simulans* and *D. sechellia* were represented by two recent, second generate assemblies of the original reference lines ([S15], [S16], respectively). Both genomes were aligned to the UCSC dm3 assembly using TBA and combined with the remaining nine species (plus *D. melanogaster*) using Multiz [S17]. The resulting alignments, liftover chain files, and alignment parameters are available upon request. To reduce artifacts due to alignment errors in our lineage-specific tests, we masked all repetitive sequences. For INSIGHT analyses, we additionally masked and excluded sites associated with indels and regions of low coverage (less than one read per sample on average) in the DGRP.

INSIGHT analyses was conducted following the methods described in the original paper [S13]. Briefly, we calculated our baseline evolutionary models by fitting a General Time Reversible model to 4d-sites across our alignment using the program phyloFit in the PHAST package [S11]. This model was fit to each chromosome arm independently, though the results varied little in practice for the euchromatic regions of the genome. To obtain the conditional probability of the ancestral nucleotide sequences, we first masked the *D. melanogaster* reference genome and obtained the "posterior" ancestral state probabilities using phyloFit/prequel in the PHAST package. To account for local variation in mutation rates and coalescent times, we tiled

the genome with 5kb overlapping windows within which we masked all sites contained within phastCons blocks or within 25bp of exonic sequence, DNaseI peaks, peaks of H3Kme3, or containing genotype information for fewer than 400 haplotypes (200 diploid individuals) to generate a neutral reference. We then sampled randomly at each base 400 haplotypes and used these sites to calculate an estimate of the population parameter theta (Waterson's estimator) with polymorphic sites in the block contributing to INSIGHT's global estimate of expected neutral allele frequencies. Within each block, we also obtained block-specific estimates of the divergence rate along the *D. melanogaster* lineage by masking all sites polymorphic in *D. melanogaster* and estimating a new, *D. mel*-specific scaling factor for our original neutral tree using phyloFit. We then associated each of these estimates with a unique, non-overlapping, 2.5kb window. These block-specific parameters were then stored in a central database to be accessed for individual INSIGHT runs.

Lineage-specific tests for selection were carried out on each regulatory element independently using the program phyloP [S10], which carries out formal likelihood-ratio tests for evolutionary scenarios (including branch-specific acceleration relative to a subtree) using as a null evolutionary models estimated from neutral sequences (in this case, 4d-sites). For these tests, we constructed neutral models as described above, but allowing four rate categories drawn from a discrete gamma distribution to better reflect the diversity of potential selective forces acting in any given region.

Deletion validation and CRM cloning

The deletion of shadow enhancers by structural variants in the five loci was validated by PCR. Genomic DNA was phenol-chloroform extracted from ~30 adult flies in isogenic DGRP lines

that deleted the enhancer. We performed PCR using 200 ng of genomic DNA (primers provided in Table S5) from the isogenic DGRP line and a reference line (Bloomington Drosophila Stock Center, stock number: 2057).

Enhancers were directionally cloned into the previously described pDuo2n – attB vector [S2] in the MCS cassette upstream the *lacZ* reporter gene, using BglII, KpnI, or AscI restriction sites. The sequences of all enhancers were validated through standard Sanger sequencing at both the plasmid cloning step and from the final fly stock.

Transgenic reporter assay

All constructs were injected with standard methods in line J27 (Basler lab [S18]) so that integration occurred in chromosomal position 51C. Transgenic lines were balanced and homozygosed on chromosome 2 and tested by multiplex fluorescent in situ hybridization (ISH) using anti-sense RNA probes labeled with Digoxigenin (DIG), Biotin (BIO), or Fluorescein (FITC), which were developed using tyramide signal amplification. Enhancer activity was visualized by fluorescent in situ hybridization against *lacZ* (green channel) with appropriate marker genes (magenta); *twist* (*twi*) for early mesoderm, *biniou* (*bin*) for the VM, *Mef2* for a general mesoderm/muscle marker. All images were taken on a Zeiss LSM 510 META confocal microscope.

Supplemental references

S1. Kvon, E. Z., Kazmar, T., Stampfel, G., Yáñez-Cuna, J. O., Pagani, M., Schernhuber, K., Dickson, B. J., and Stark, A. (2014). Genome-scale functional characterization of Drosophila developmental enhancers in vivo. Nature *512*, 91–95.

S2. Zinzen, R. P., Girardot, C., Gagneur, J., Braun, M., and Furlong, E. E. M. (2009). Combinatorial binding predicts spatio-temporal cis-regulatory activity. Nature *462*, 65–70.

S3. Zichner, T., Garfield, D. A., Rausch, T., Stütz, A. M., Cannavo, E., Braun, M., Furlong, E. E. M., and Korbel, J. O. (2013). Impact of genomic structural variation in Drosophila melanogaster based on population-scale sequencing. Genome Res *23*, 568–579.

S4. Ye, K., Schulz, M. H., Long, Q., Apweiler, R., and Ning, Z. (2009). Pindel: a pattern growth approach to detect break points of large deletions and medium sized insertions from paired-end short reads. Bioinformatics *25*, 2865–2871.

S5. Rausch, T., Zichner, T., Schlattl, A., Stütz, A. M., Benes, V., and Korbel, J. O. (2012). DELLY: structural variant discovery by integrated paired-end and split-read analysis. Bioinformatics *28*, i333–i339.

S6. Handsaker, R. E., Korn, J. M., Nemesh, J., and McCarroll, S. A. (2011). Discovery and genotyping of genome structural polymorphism by sequencing on a population scale. Nat Genet *43*, 269–276.

S7. Abyzov, A., Urban, A. E., Snyder, M., and Gerstein, M. (2011). CNVnator: an approach to discover, genotype, and characterize typical and atypical CNVs from family and population genome sequencing. Genome Res *21*, 974–984.

S8. Huang, W., Massouras, A., Inoue, Y., Peiffer, J., Ràmia, M., Tarone, A. M., Turlapati, L., Zichner, T., Zhu, D., Lyman, R. F., et al. (2014). Natural variation in genome architecture among 205 Drosophila melanogaster Genetic Reference Panel lines. Genome Res *24*, 1193–1208.

S9. Siepel, A. (2005). Evolutionarily conserved elements in vertebrate, insect, worm, and yeast genomes. Genome Res *15*, 1034–1050.

S10. Pollard, K. S., Hubisz, M. J., Rosenbloom, K. R., and Siepel, A. (2010). Detection of nonneutral substitution rates on mammalian phylogenies. Genome Res *20*, 110–121.

S11. Siepel, A., and Haussler, D. (2004). Phylogenetic estimation of context-dependent substitution rates by maximum likelihood. Mol Biol Evol *21*, 468–488.

S12. Garfield, D., Haygood, R., Nielsen, W. J., and Wray, G. A. (2012). Population genetics of cis-regulatory sequences that operate during embryonic development in the sea urchin Strongylocentrotus purpuratus. Evol Dev *14*, 152–167.

S13. Gronau, I., Arbiza, L., Mohammed, J., and Siepel, A. (2013). Inference of natural

selection from interspersed genomic elements based on polymorphism and divergence. Mol Biol Evol *30*, 1159–1171.

S14. Gulko, B., Hubisz, M. J., Gronau, I., and Siepel, A. (2015). A method for calculating probabilities of fitness consequences for point mutations across the human genome. Nat Genet *47*, 276–283.

S15. Hu, T. T., Eisen, M. B., Thornton, K. R., and Andolfatto, P. (2013). A second-generation assembly of the Drosophila simulans genome provides new insights into patterns of lineage-specific divergence. Genome Res *23*, 89–98.

S16. Coolon, J. D., McManus, C. J., Stevenson, K. R., Graveley, B. R., and Wittkopp, P. J. (2014). Tempo and mode of regulatory evolution in Drosophila. Genome Res *24*, 797–808.

S17. Blanchette, M., Kent, W. J., Riemer, C., Elnitski, L., Smit, A. F. A., Roskin, K. M., Baertsch, R., Rosenbloom, K., Clawson, H., Green, E. D., et al. (2004). Aligning multiple genomic sequences with the threaded blockset aligner. Genome Res *14*, 708–715.

S18. Bischof, J., Maeda, R. K., Hediger, M., Karch, F., and Basler, K. (2007). An optimized transgenesis system for Drosophila using germ-line-specific phiC31 integrases. Proc Natl Acad Sci USA *104*, 3312–3317.
