# Supplementary material for: Shadow Enhancers Are Pervasive Features of Developmental Regulatory Networks
Source: Curr Biol. 2016 Jan 11;26(1):38–51. doi: 10.1016/j.cub.2015.11.034 (PMC4712172; doi:10.1016/j.cub.2015.11.034)
Supplement: Document S2. Article plus Supplemental Information [file mmc8.pdf]

Article

Current Biology

Shadow Enhancers Are Pervasive Features of Developmental Regulatory Networks

Highlights

- Regulation by shadow enhancers is pervasive and complex during embryonic development
- The vast majority of genes have more than two enhancers with similar activity
- Shadow enhancers buffer genetic variation within a population, but yet appear conserved
- Evolutionary analyses suggest that they play complex and fundamental roles in development

Authors

Enrico Cannavò, Pierre Khoueiry, David A. Garfield, ..., Lucia Ciglar, Jan O. Korbel, Eileen E.M. Furlong

Correspondence

furlong@embl.de

In Brief

Cannavò et al. examine redundant (shadow) enhancers genome wide, finding that the majority of loci have more than two elements with similar activity. Evolutionary analyses show evidence of pervasive stabilizing selection and an ability to buffer mutations, suggesting that shadow enhancers have complex and fundamental roles in developmental networks

CrossMark

Cannavò et al., 2016, Current Biology *26*, 38–51
January 11, 2016 ©2016 The Authors
http://dx.doi.org/10.1016/j.cub.2015.11.034

CellPress

Current Biology
Article

Shadow Enhancers Are Pervasive Features of Developmental Regulatory Networks

Enrico Cannavò,[1] Pierre Khoueiry,[1] David A. Garfield,[1] Paul Geeleher,[1] Thomas Zichner,[1] E. Hilary Gustafson,[1] Lucia Ciglar,[1] Jan O. Korbel,[1] and Eileen E.M. Furlong[1],*
[1]European Molecular Biology Laboratory (EMBL), Genome Biology Unit, 69117 Heidelberg, Germany
*Correspondence: furlong@embl.de
http://dx.doi.org/10.1016/j.cub.2015.11.034
This is an open access article under the CC BY license (http://creativecommons.org/licenses/by/4.0/).

SUMMARY

Embryogenesis is remarkably robust to segregating mutations and environmental variation; under a range of conditions, embryos of a given species develop into stereotypically patterned organisms. Such robustness is thought to be conferred, in part, through elements within regulatory networks that perform similar, redundant tasks. Redundant enhancers (or "shadow" enhancers), for example, can confer precision and robustness to gene expression, at least at individual, well-studied loci. However, the extent to which enhancer redundancy exists and can thereby have a major impact on developmental robustness remains unknown. Here, we systematically assessed this, identifying over 1,000 predicted shadow enhancers during *Drosophila* mesoderm development. The activity of 23 elements, associated with five genes, was examined in transgenic embryos, while natural structural variation among individuals was used to assess their ability to buffer against genetic variation. Our results reveal three clear properties of enhancer redundancy within developmental systems. First, it is much more pervasive than previously anticipated, with 64% of loci examined having shadow enhancers. Their spatial redundancy is often partial in nature, while the non-overlapping function may explain why these enhancers are maintained within a population. Second, over 70% of loci do not follow the simple situation of having only two shadow enhancers—often there are three (*rols*), four (*CadN* and *ade5*), or five (*Traf1*), at least one of which can be deleted with no obvious phenotypic effects. Third, although shadow enhancers can buffer variation, patterns of segregating variation suggest that they play a more complex role in development than generally considered.

INTRODUCTION

Developmental robustness is achieved through buffering gene expression patterns against stochastic, genetic, and environmental perturbations [1–9]. Although the underlying molecular mechanisms are still being dissected, transcriptional robustness can be modulated at several levels [10, 11], including DNA accessibility [12], RNA polymerase II pausing [13, 14], and promoter organization [15–17]. It can also arise from higher levels of network organization [18–22], including functional redundancy, defined as two parts of a system that can perform the same or similar tasks and are therefore not individually essential [23].

A potential contributor to functional redundancy is regulatory elements with overlapping functions. A number of studies in vertebrates [4, 8], invertebrates [2, 24], and plants [25] have identified enhancers that appear to act redundantly—defined as two enhancers that drive similar patterns of expression and in which deletion of one did not cause any obvious aberrant phenotypes [4, 8]. There are a number of well-characterized examples of such shadow enhancers acting during embryonic development [2]. In the *pax3* locus, for example, two enhancers direct expression in neural crest cells [1–9]. Although the proximal 5′ element, when placed upstream of *pax3* cDNA, is sufficient to rescue neural crest cell development in mice lacking endogenous *pax3*, this enhancer is not required for development or viability. Similarly in the *TCRgamma* locus, deletion of either the HsA or 3′E (Cgamma1) enhancers has little effect on *TCRgamma* transcription, whereas deletion of both elements causes a severe reduction in transcription and defects in gammadelta thymocyte development [9]. Interestingly, although both enhancers act redundantly in gammadelta thymocytes, in a different cell context, the HsA enhancer acts non-redundantly with the 3′E element to regulate gene expression [9].

Although examples of redundant enhancers have been known for over 20 years, recent studies in *Drosophila* have reignited the debate over the prevalence and functional role of these elements in the regulation of gene expression. When examining the binding patterns of three transcription factors (TFs), Hong et al. observed that in addition to a gene's well-characterized enhancer, many early patterning genes in *Drosophila* have a second element with very similar TF occupancy [2]. These shadow enhancers frequently regulate highly similar, overlapping patterns of expression in transgenic reporter assays, suggesting that they act redundantly [2, 5, 6]. For example, each of the five gap gene loci in the *Drosophila* segmentation pathway contain an additional shadow enhancer [7]. Shadow enhancers can provide robustness to genetic variation within a population, allowing development to proceed unperturbed, as shown at a number of well-characterized loci [4, 8]. However, whether this is their primary function remains unclear as they appear to have multiple functions in the

regulation of gene expression. For example, in some cases, enhancers that appear to act redundantly due to their overlapping activity are actually both essential to define the precise spatial, in the case of *snail* [26], or temporal, in the case of *brinker* [27], pattern of that gene's expression. Alternatively, they may act redundantly, controlling the levels of a gene's expression at one stage of lifespan (e.g., in adults), but act more synergistically during another (e.g., embryogenesis), as recently observed at the mouse *Pomc* locus [28]. Similarly, enhancers that appear to act redundantly under normal environmental conditions can be essential under more stressful conditions, as demonstrated in the *shavenbaby* (*svb*) [3] and *snail* [5] loci. Genes with redundant enhancers also tend to initiate their expression more synchronously during very rapid cell divisions, illustrating another context in which these elements help ensure robust expression during development [7]. These examples question the extent to which enhancers with redundant activity in one context are completely redundant across the entire spectrum of the enhancer's activity (which we refer to as absolute redundancy).

The examples above demonstrate that individual enhancers can act to canalize their target gene's expression, buffering them against environmental and genetic perturbations. However, for shadow enhancers to act as major contributors to developmental robustness, they should be much more prevalent than the handful of examples known to date. Just how extensive redundant enhancers are, and to what extent overlapping enhancers are truly redundant, remains unclear. To directly assess this, we performed the first genome-wide assessment of the prevalence and global properties of shadow enhancers using the developing *Drosophila* mesoderm as a model system. Using two stringent approaches, we identified 1,055 shadow enhancers associated with 319 unique genes. For 23 enhancers at five loci, we examined their in vivo activity throughout all stages of embryonic development. This revealed a regulatory landscape that is considerably more complex than the simple "one shadow to one main enhancer" relationship. Rather, the majority of loci contain three, four, or even as many as five shadow enhancers. When one shadow enhancer is deleted in each of these five loci, there was little obvious effect on embryonic development, suggesting that they can buffer the effects of genetic variation and are thus redundant. However, contrary to expectations for enhancers with absolute redundancy, shadow enhancers are more conserved than non-redundant enhancers, show a higher proportion of functional sites, and show neither evidence of relaxed selection in natural populations nor enrichment for lineage-specific adaptive events, observations that are most consistent with pervasive stabilizing selection. These conservation patterns may be a result of selection for robustness per se [29]. Alternatively, they may equally be a side product of the modular nature of developmental programs—when multiple enhancers are required to regulate complex patterns of expression, a degree of robustness may be an inevitable, very useful, byproduct.

RESULTS

Enhancers with Complete Spatial Redundancy Are Rare, Whereas Partial Redundancy Is Common

The term redundancy, where two parts have the same function, is generally perceived as absolute redundancy. However, the examples presented above show clear cases in which enhancers act 100% redundantly in one context (tissue A, time point 1, or normal environmental conditions) and yet are essential in another [tissue B (e.g., HsA [9] and *snail* [26]), time point 2 (e.g., *brinker* [27]), or adverse environmental conditions (e.g., *svb* [3] and *snail* [5])], a property we refer to as partial redundancy. Enhancers with absolute redundancy are often generated through duplication events [30] and then either functionally diverge or degrade, being rapidly lost within a population. Partially redundant elements, i.e., enhancers with overlapping spatial activity, in contrast, should be maintained by selection and therefore preserved over longer evolutionary timescales (e.g., [31]) and thus should be more common.

It is now possible to assess this reasoning, given the recent availability of a very large collection of 7,705 enhancers covering ~15% of the non-coding *D. melanogaster* genome, whose detailed in vivo activity was annotated with 227 tissue terms throughout all stages of *Drosophila* embryogenesis in stable transgenic embryos [32]. We therefore first determined whether enhancers with overlapping spatial activity (partial redundancy) are more prevalent within a genome compared to enhancers with identical activity (absolute redundancy). Only enhancers with a single DNaseI-hypersensitive (DHS) site were included in the analysis, to exclude ambiguity caused by cases where multiple enhancers may be contained within the same 2 kb region tested in transgenic embryos (Supplemental Experimental Procedures). Overall, enhancers located within 50 kb of each other are much more likely to exhibit similar, overlapping spatial activity ($p = 2.3 \times 10^{-34}$; Figure 1A; Supplemental Experimental Procedures). However, they are not more likely to exhibit identical activity than expected by chance ($p = 0.79$; Figure 1B). As expected, these results indicate that even when considering a very broad and diverse set of spatiotemporal patterns, absolute redundancy of enhancer activity for spatial expression is rare, though some level of redundancy (overlapping spatial activity) is present and likely to be functionally important. Interestingly, this is not the case at the gene level. Genes within a 50 kb window of each other are both more likely to have overlapping spatial expression ($p = 2.6 \times 10^{-61}$) and identical expression ($p = 0.001$) than is expected by chance (Figures 1C and 1D; Supplemental Experimental Procedures).

Genome-wide Identification of Enhancers with Highly Correlated Activity

The analyses above suggest that enhancers with partially redundant activity are much more frequent than enhancers with absolute redundant activity. However, the frequency of these elements throughout the entire genome remains unclear; the authors identified 16 genes (out of 116 examined) with shadow enhancers [32]. To examine the prevalence of shadow enhancers more globally, we used two stringent approaches, focusing on the mesoderm and its derivatives. The first approach is based on Perry et al. [7], who defined prospective shadow enhancers for eight gap genes as pairs of genomic regions cobound by the same TFs within 100 kb of the genes' promoter. Here, we extended this approach and more formally identified highly correlated TF occupancy across 15 conditions using chromatin immunoprecipitation (ChIP) data for five mesodermal TFs across multiple developmental stages [33]. Importantly, 97% of these ChIP-defined *cis*-regulatory modules (CRMs) function as developmental enhancers when tested in vivo using transgenic

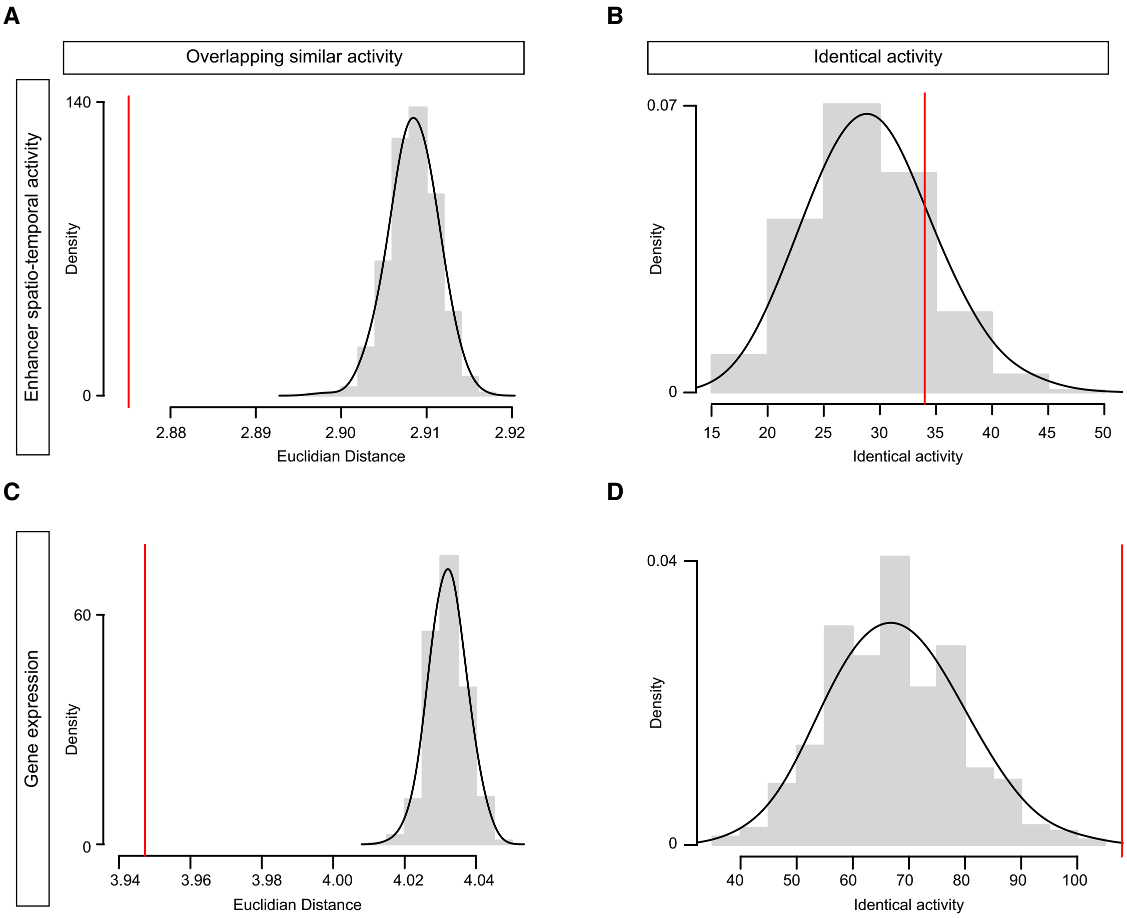

Figure 1. Frequency of Enhancers Pairs with Similar versus Identical Activity

The level of similarity (partial overlap) in tissue expression of enhancers (A and B) and genes (C and D) within 50 kb windows of each other, compared to what would be expected by chance. Vertical red line represents the observed data, and the histogram and associated density plots show the values achieved from randomly shuffling enhancers/genes in the genome.

(A) The Euclidean distance was used to summarize the (multidimensional) distance between pairs of enhancers in tissue expression space. Plotted here is the median Euclidean distance for the observed (red line) and expected (gray histogram) distribution of enhancers with similar tissue expression.

(B) Number of co-located enhancers with identical spatiotemporal activity.

(C) Median Euclidean distance (measure of similarity in multidimensional tissue expression) between co-located genes (within 50 kb of each other).

(D) Number of co-located genes with identical patters of expression.

reporter assays [33]. Spearman rank-correlations between TF ChIP intensities was scanned across all 8,008 ChIP-defined enhancers within a 50 kb distance of each other (illustrated in Figure 2A) and in the vicinity of a gene with mesoderm and/or muscle expression, using in situ hybridization data (Supplemental Experimental Procedures). This identified a stringent set of shadow enhancers with highly correlated TF occupancy to at least one other enhancer associated to the same target gene (Table S1). An example of one such pair is shown in Figure 2A.

Although enhancers bound by the same combination of TFs often give rise to similar patterns of expression, a number of studies indicate more complex relationships. Enhancers with diverse patterns of TF occupancy [33–35] and regulatory logic [36] can, for example, also give rise to highly similar spatial activity. As the functional output of an enhancer is the important property for development, this is the parameter most likely under selection. This observation led us to our second approach, were we defined shadow enhancers based on their overlapping spatial activity. As there are no genome-wide data for enhancer spatiotemporal activity, we made use of our previously validated method, which predicted the activity of 8,008 mesodermal enhancers from TF occupancy data using a machine-learning approach trained on enhancers with characterized activity [33]. Each of the 8,008 ChIP-defined enhancers thereby has a probability score of being active in one of four exclusive tissue classes (Supplemental Experimental Procedures); importantly 83% of these tissue predictions hold true, i.e., the enhancers drove expression in the predicted tissue when tested in vivo in transgenic embryos [33] (Figure 2B). Shadow enhancers were defined as pairs of elements having a high-confidence prediction within the same tissue (SVM specificity score ≥0.95), being within 50 kb of each other, and associated with a common gene with overlapping expression from in situ hybridization (Figure 2B; Supplemental Experimental Procedures). This resulted in a

CellPress

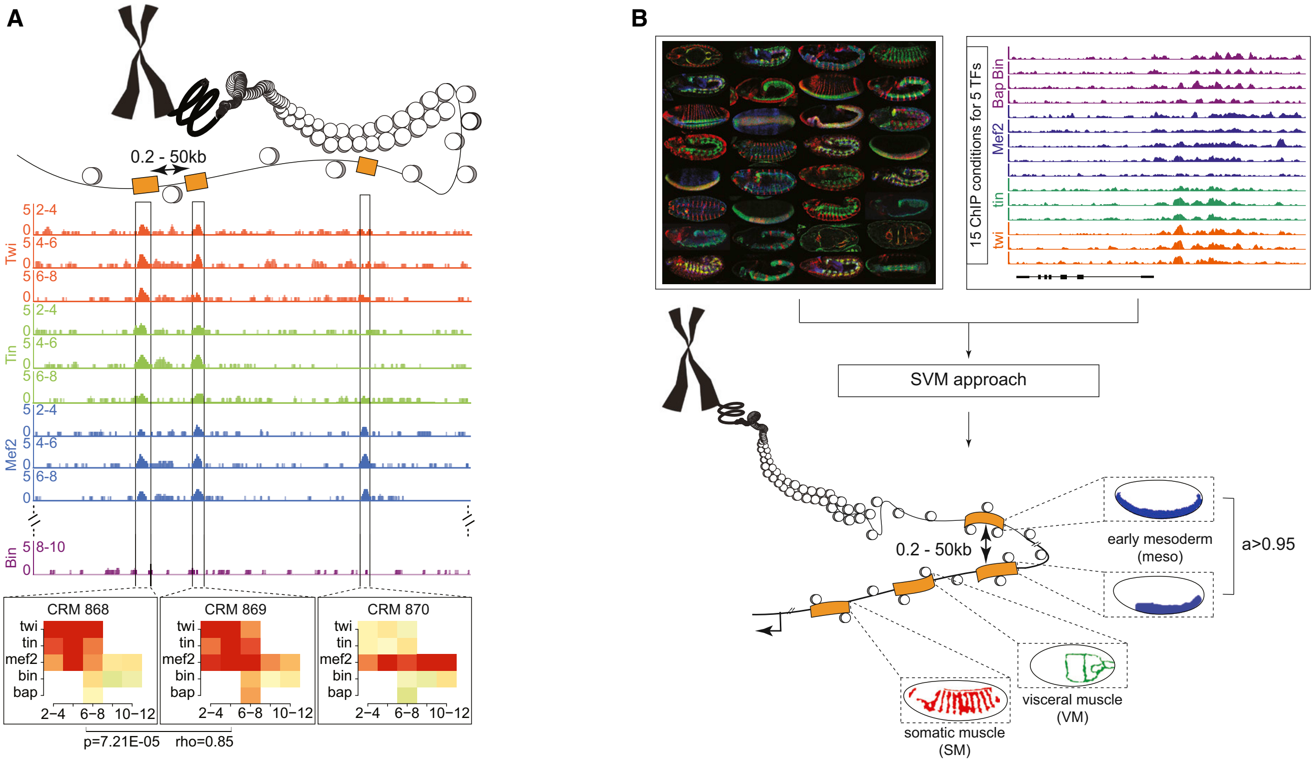

Figure 2. Genome-wide Prediction of Shadow Enhancers

Two complementary criteria used to identify shadow enhancers throughout the genome:

(A) Enhancers with highly correlated TF occupancy, using ChIP data from 15 conditions for mesoderm/muscle TFs [33], within 50 kb of each other and an associated gene with mesoderm and/or muscle expression. An example of a locus with two enhancers (CRM868 and CRM869) that have highly correlated binding (rho = 0.85; predicted shadow enhancers) compared to one that is not (CRM870) is shown. The bottom panels show the similarity (or dissimilarity) of TF binding as heatmaps using the ChIP peak height (red, high, to white, unbound), with TFs indicated on the y axis and developmental time (hr) on the x axis.

(B) Enhancers with similar activity, predicted for all 8,008 mesodermal enhancers using a support vector machine (SVM) [33]. Enhancers within a 50 kb window of each other, with the same predicted expression (SVM score ≥ 0.95) and associated with a gene expressed in the same tissue were defined as shadow enhancers. The predicted tissue expression of enhancers is represented by cartoons.

In (A) and (B), ChIP-chip data for the TFs Twist (red), Tin (green), Mef2 (blue), and Bin (purple) at different developmental time windows (2–4 hr, 4–6 hr, 6–8 hr, and 8–10 hr) are shown.

stringent set of 866 shadow enhancers associated to 298 genes with mesoderm and/or muscle expression (Table S1).

The combination of these two approaches identified 1,055 shadow enhancers predicted to have similar activity to at least one other enhancer during mesoderm and/or muscle development (Table S1). Approximately 40% of genes are regulated by a single pair of shadow enhancers, in keeping with the vast majority of current examples of redundant enhancers in both *Drosophila* [2, 6, 7] and mice [1–9], with the notable exception of *vnd* [37]. However, the majority of genes appear to have much more complex regulation, with ~60% of loci with shadow enhancers containing three (77 genes) or four (40 genes), and even a few examples of five (14 genes), six (ten genes), seven (seven genes), or eight (two genes), shadow enhancers with (predicted) similar activity (Figure S1A), suggesting that the current view of potential redundancy is over simplistic.

Shadow Enhancers Can Buffer the Effects of Natural Sequence Variation

By definition, redundant or partially redundant enhancers can compensate for mutations that render one of the enhancers dysfunctional, as shown in the *svb* [3] and *dac* [5] loci in *D. melanogaster* or the *Hoxd* loci in mouse [4, 8]. If the shadow enhancers are acting redundantly, the transcriptional program driving embryogenesis should be able to proceed if one of the two enhancers is deleted. To examine this, we used natural sequence variation within a wild population of *Drosophila* to determine whether enhancers within a predicted redundant pair are affected by deleterious mutations. As it is often difficult to predict the effect of an individual SNP on TF occupancy [38, 39], we focused here on deletions (structural variations, SVs) greater than 50 bp that intersected the center of the enhancer and deleted at least 25% of its size. For this, we took advantage of a set of 205 fully sequenced inbred homozygous lines from Drosophila Genetic Reference Panel (DGRP) [40] and extended SV calls that we generated on 40 lines [41, 42] to all 205 lines and combined these with the Berkeley Drosophila Genome Project (BDGP) consortium's freeze2 calls [41, 42] (Supplemental Experimental Procedures; Table S4).

We first examined how often SVs affect different functional parts of the genome, such as exons, introns, and enhancers (Figure S1B). To assess the significance of these results, we

performed simulations in which SVs were randomly moved 1,000 times by up to 50 kb up- or downstream, and then reassessed the overlap with the functional elements for each iteration (Figure S1B). Overall, exons are strongly depleted in deletions when comparing the overlap in the number of observed and simulated events, while introns show a similar (but much weaker) trend. The frequency of developmental enhancers' deletion by SVs within natural populations is in between that of exons and introns (Figure S1B), emphasizing their importance in the genome.

Next, we examined whether there was a difference in the prevalence of deletions affecting shadow enhancers compared to non-redundant enhancers associated with mesoderm and/or muscle genes. 151 shadow enhancers are affected by an SV, compared to only 27 non-redundant enhancers, a difference that is borderline significant (odds ratio = 1.47, p = 0.04 from a one-sided Fisher's exact test), numbers that would certainly increase as more genotypes are sequenced. This result is consistent with the ability of shadow enhancers to buffer against the consequences of genetic perturbations during embryonic development (Figure S1).

The flies harboring these homozygous SVs are alive and viable, at least under laboratory conditions, so even when one of these developmental enhancers is deleted, embryogenesis proceeds largely normally, indicating that the loss of function of this enhancer is compensated by the presence of a second (or third, or fourth) shadow enhancer.

Predicted Shadow Enhancers Function as Shadow Enhancers In Vivo

To confirm that our predicted shadow enhancers do indeed regulate similar overlapping patterns of expression, we examined the spatiotemporal activity of 23 elements within five loci in vivo using transgenic reporter assays (Supplemental Experimental Procedures). We purposely selected complex regions, where more than a simple pair of shadow enhancers was predicted by at least one method and where naturally occurring SVs removed one or more of the predicted shadow enhancers.

At all five loci, we validated the SV calls by PCR on individual DGRP lines (Figure S2). We then examined the activity of 15 predicted shadow enhancers, as well as eight other enhancers within these loci that were just below the stringent thresholds applied for activity prediction (SVM > 0.95) or correlated TF binding (rho > 0.8). Each of the 23 enhancer elements, which were on average 512 bp in length, were cloned into a common minimal *lacZ* reporter vector and stably integrated into the same location in the *Drosophila* genome using the phiC31 system [43] to allow for a direct comparison of enhancer activities in the same genomic context. The ability of each enhancer to drive spatiotemporal *lacZ* expression was assessed during all stages of embryogenesis by double fluorescence in situ hybridization (FISH) against the *lacZ* reporter and a gene with mesodermal and/or muscle expression.

At all five loci, we observed overlapping spatial activity from multiple enhancers, validating the predicted shadow enhancers' activity in all cases. The *rolling pebbles* (*rols*) and *CG42788* loci both contain multiple enhancers with predicted redundant activity. The *rols* gene codes for an essential protein that forms part of a multiprotein complex essential for myoblast fusion [44, 45]. Loss-of-function mutant embryos fail to hatch due to a severe defect in myoblast fusion and therefore don't survive beyond embryogenesis [44, 45]. Of the three predicted shadow enhancers examined (Figures 3A and 3B), CRM4347 is deleted by an SV in 11 of the 205 isogenic *Drosophila* lines (Table S4). All three enhancers drive reporter gene expression in overlapping spatial domains at stage 11 and 12 (Figures 3C and S3), despite a clear difference in TF occupancy (data not shown). As predicted by the SVM approach, the three enhancers are active in the visceral and somatic muscle, each partially recapitulating the expression of the endogenous *rols* gene. Similarly, the *CG42788* locus contains three predicted shadow enhancers based on their highly correlated TF occupancy or predicted visceral mesoderm activity (Figures 3D and 3E). Examination of *lacZ* reporter gene expression in transgenic embryos revealed that two of the three enhancers drive overlapping expression in the trunk visceral muscle at stages 15–17 of embryogenesis (Figures 3F and S4).

The *ade5* locus contains four shadow enhancers based on their predicted activity. *ade5* regulates de novo purine synthesis and is essential for viability [46]. CRM7490 regulates expression in the somatic and visceral mesoderm from stage 11 to stage 14 of embryogenesis and is completely deleted by an SV in an isogenic line that is viable and fertile (Figure 4). Three other shadow enhancers (CRM7483, CRM7487/8, and CRM7489) regulate overlapping spatiotemporal activity to the deleted enhancer, driving *lacZ* expression either only in the visceral mesoderm or in both the somatic and visceral mesoderm at some or all developmental stages (Figures 4C and S5). This complex locus demonstrates partial redundancy at both a spatial and temporal level, where many enhancers with overlapping expression are likely to be involved in the generation of robust and specific gene expression patterns.

Three shadow enhancers within the first intron of the large isoform of *Traf1* are all predicted to be active in the early mesoderm (Figure 5). One of these enhancers is almost completely removed by an SV in two out of 205 individuals. The *Traf1* gene encodes a member of the tumor necrosis factor receptor superfamily. Loss-of-function *Traf1* mutants fail to develop beyond larval stages due to defects in imaginal disc and brain development [47]. We generated transgenic embryos for the three predicted shadow enhancers, as well as two other enhancers within the locus that had predicted mesoderm activity just below our SVM cutoff (<0.95; Figure 5B). Examination of *lacZ* expression revealed that all five enhancers have overlapping activity in the presumptive mesoderm at stage 6 of development, ranging from almost the entire mesoderm (CRM5429, CRM5432, and CRM5435/6) to subsets of mesodermal cells (CRM5437 and CRM5440) (Figure 5C). Therefore, although the total spatial expression pattern of each enhancer varies, they are all active in a population of mesodermal cells at the same stage of development. These results highlight the complexity of *Traf1*'s transcriptional regulation and the extent to which enhancer activity may be buffered by additional elements regulating expression in the same cells at a given stage of development.

A similarly complex example is the *Cadherin-N* (*CadN*) locus. *CadN* is essential for cell-cell interactions during many process of development, including mesoderm gastrulation and the embryonic nervous system [48, 49]. In this locus, we predicted two shadow enhancers based on their predicted activity (Figure 6), CRM6248 and CRM6250, one of which (CRM6248) is almost completely deleted by an SV in two isogenic lines

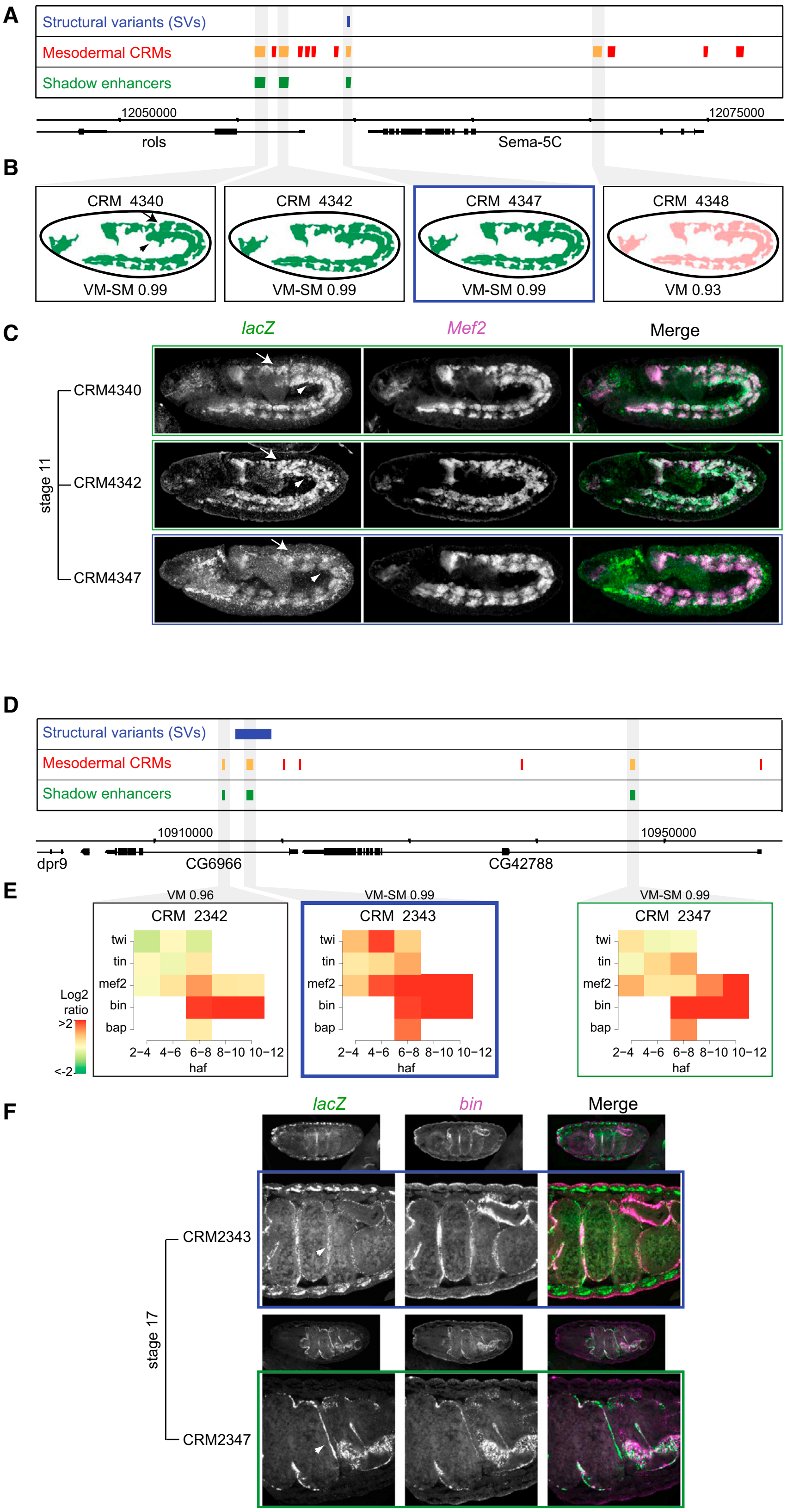

Figure 3. Shadow Enhancers in the *rols* and *CG42788* Loci

Predicted shadow enhancers based on similarity in activity (*rols*; A–C) or TF occupancy (*CG42788*; D–F).

(A) *rols* locus showing structural variants (blue), mesodermal *cis*-regulatory modules defined by TF-ChIP (CRMs; red), and shadow enhancers (green).

(B) Predicted spatial expression of enhancers. Tissue class and SVM score are shown at bottom. VM-SM, visceral muscle-somatic muscle; VM, visceral muscle.

(C) Double FISH of transgenic embryos showing *lacZ* reporter (green) under the transcriptional control of three shadow enhancers (CRM4340, CRM4342, and CRM4347) with the pan-mesoderm/muscle marker *Mef2* (magenta). CRM4347 is deleted by an SV (blue, A) and has overlapping expression with CRM4340 and CRM4342 (B and C). SM is indicated by arrows and VM by arrowheads in (D) and (C). A fourth enhancer, CRM4348, which was not predicted to be a shadow enhancer, drives expression in ectodermal strips (data not shown).

(D) *CG42788* locus showing structural variants (blue), mesodermal *cis*-regulatory modules (CRMs; red), and shadow enhancers (green).

(E) Three shadow enhancers predicted based on both similar activity and highly correlated TF occupancy. The heatmap shows the ChIP peak height signal for each factor/time point. SVM prediction and score are shown above.

(F) Double FISH of transgenic embryos showing *lacZ* reporter (green) under the transcriptional control of two shadow enhancers (CRM2343 and CRM2347) with the visceral muscle (VM) marker *biniou* (*bin*) (magenta). CRM2343 is completely deleted by an SV (A) and has overlapping expression with CRM2347. VM is indicated by the white arrowhead. CRM2342 did not share regions of spatial overlap with the other enhancers.

Enhancers tested in transgenic embryos are indicated in orange. All embryos oriented with anterior to the left and dorsal at the top. See also Figures S2–S4.

CellPress

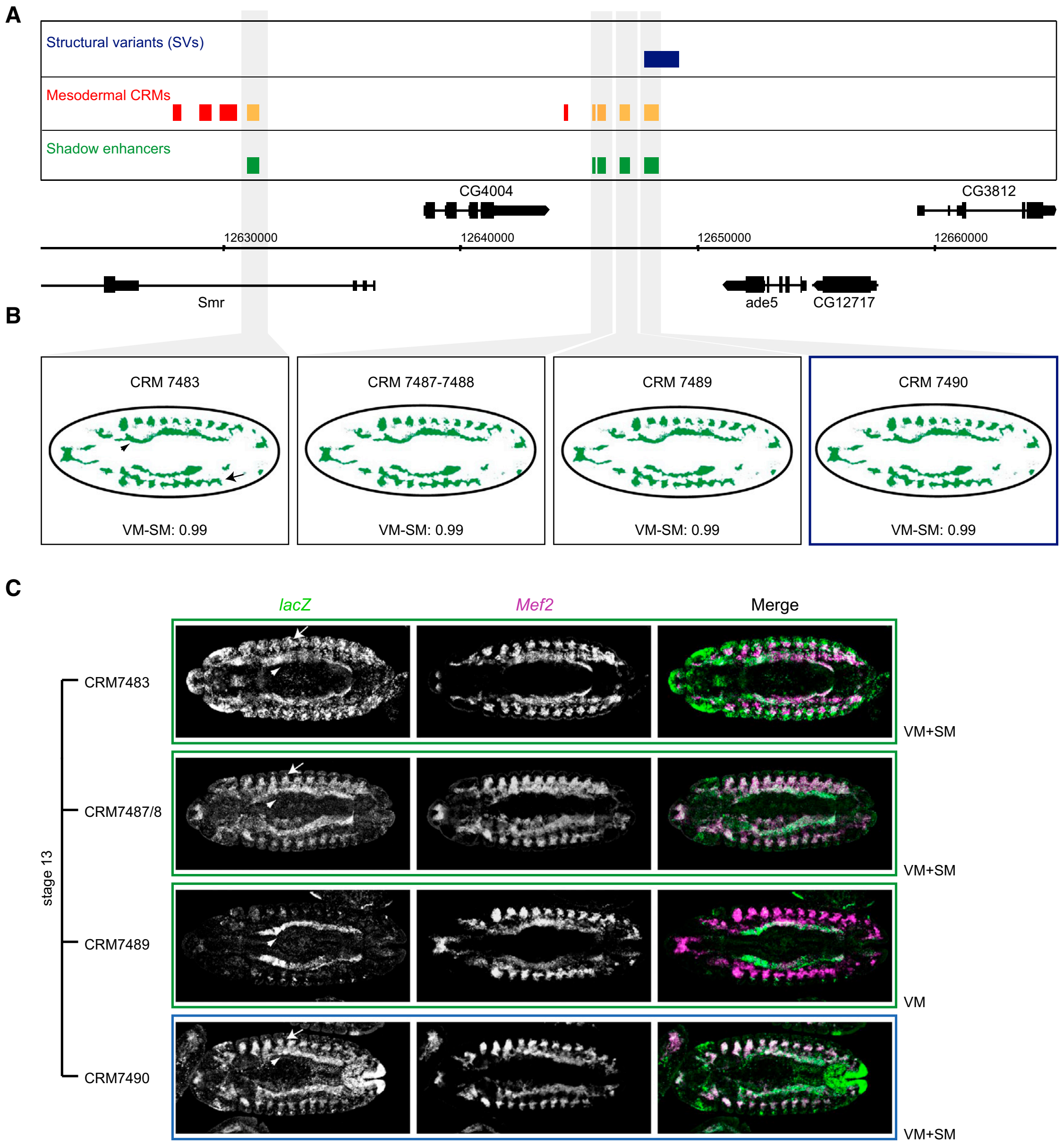

Figure 4. Shadow Enhancers in *ade5* Locus

(A) *ade5* locus showing structural variants (blue), mesodermal *cis*-regulatory modules (CRMs; red), shadow enhancers (green). Enhancers tested in transgenic embryos are indicated in orange.

(B) Predicted spatial activity of enhancers and SVM scores. VM-SM, visceral muscle-somatic muscle.

(C) Double FISH of transgenic embryos showing *lacZ* reporter (green) under the transcriptional control of four shadow enhancers (CRM7483, CRM7487/88, CRM7489, and CRM7490) with the pan-mesoderm/muscle marker *Mef2* (magenta). SM is indicated by arrows and VM by arrowheads in (B) and (C). CRM7490 is almost completely deleted by an SV (blue, A) and has overlapping expression with CRM7483, CRM7487-88, and CRM7489 (green) in VM. More stages are shown in Figure S5.

All embryos oriented with anterior to the left and dorsal at the top. See also Figures S2 and S5.

(Figure 6A). We examined the activity of both enhancers and five additional elements, two of which had similar TF binding signatures but were just below the stringent threshold applied (CRM6252/3 and CRM6254; Figure 6B). Four of the seven enhancers showed highly specific activity in the presumptive mesoderm at stage 5 and have highly overlapping activity in mesodermal cells (Figure 6C). All four enhancers are located within introns of the *CadN* gene and drive expression that is partially overlapping that of the endogenous gene.

Taken together, these data demonstrate that our prediction of shadow enhancers is very accurate, and indicate three clear properties of shadow enhancers. First, they are pervasive throughout the genome. Although this study provides a first systematic attempt to estimate how frequently this occurs, given our

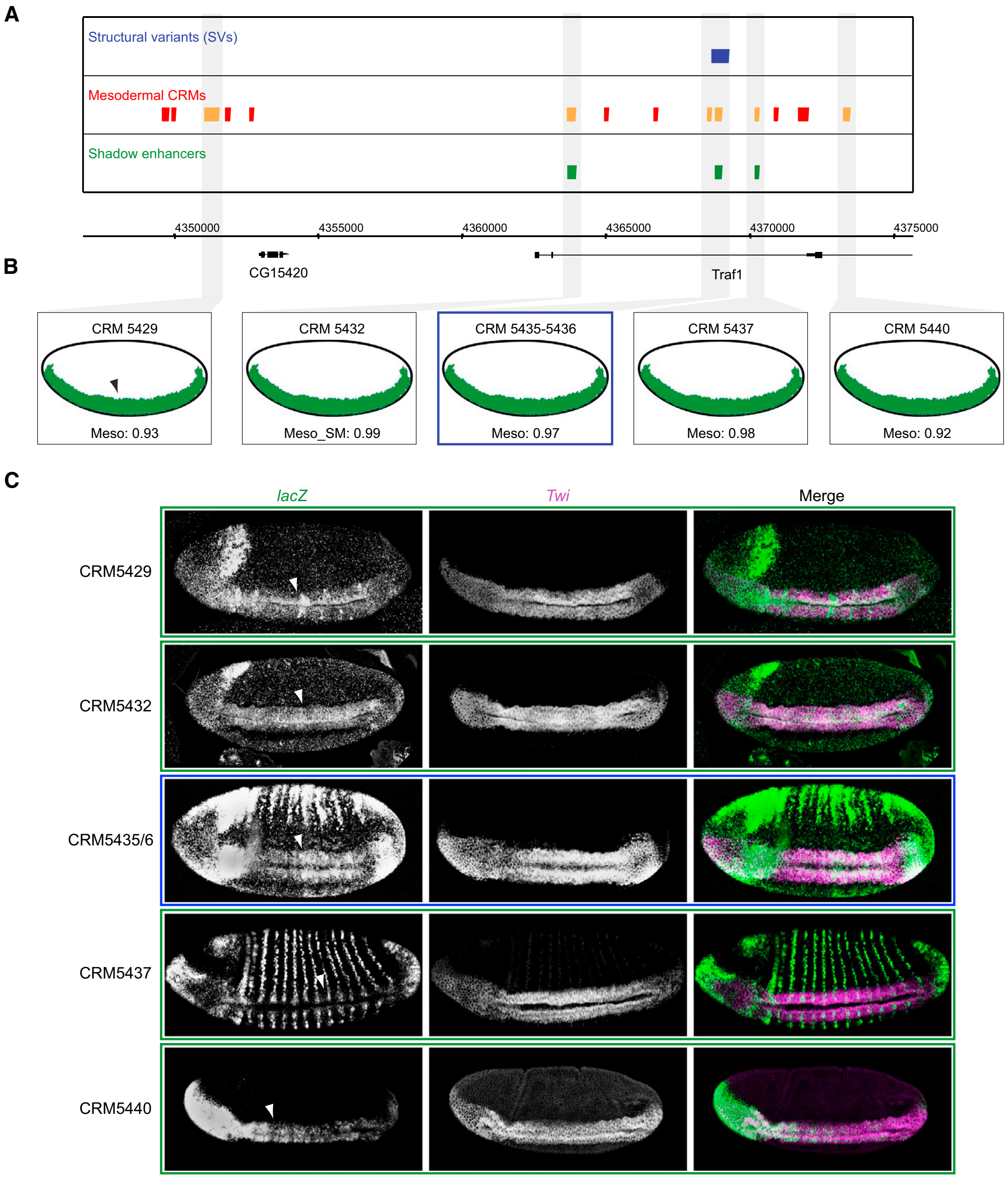

Figure 5. Complexity of *Traf1* Regulation

(A) *Traf1* locus showing structural variants (blue), mesodermal *cis*-regulatory modules (CRMs; red), and shadow enhancers (green). Enhancers tested in transgenic embryos are indicated in orange.

(B) Predicted spatial activity of enhancers and SVM scores are shown. Meso, mesoderm; Meso-SM, mesoderm and somatic muscle. Three shadow enhancers were predicted (CRM5432, CRM5435/6, and CRM5437) and two additional tested below the applied SVM specificity score (CRM5429 and CRM5440).

(C) Double FISH of transgenic embryos showing *lacZ* reporter (green) under the transcriptional control of five shadow enhancers (CRM5429, CRM5432, CRM5435/6, CRM5437, and CRM5440) with the early mesoderm marker *Twist* (*Twi*; magenta). Mesoderm is indicated by arrowheads in (B) and (C). CRM5435/6 is almost completely deleted by an SV (blue, A) and has overlapping expression with CRM5429, CRM5432, and CRM5440 in the mesoderm.

All embryos oriented with anterior to the left and dorsal at the top. See also Figures S2 and S5.

conservative thresholds, these predictions are clearly underestimating the number of enhancers with similar, overlapping activity within a given gene's locus. Second, the level of potential redundancy is much more complex than typically envisaged. In over half of the cases, it is not simply two enhancers that may act redundantly; often there are three (*rols*), four (*CadN* and *ade5*) or even five (*Traf1*) enhancers with overlapping activity. Third, this extensive level of potential *cis*-regulatory redundancy

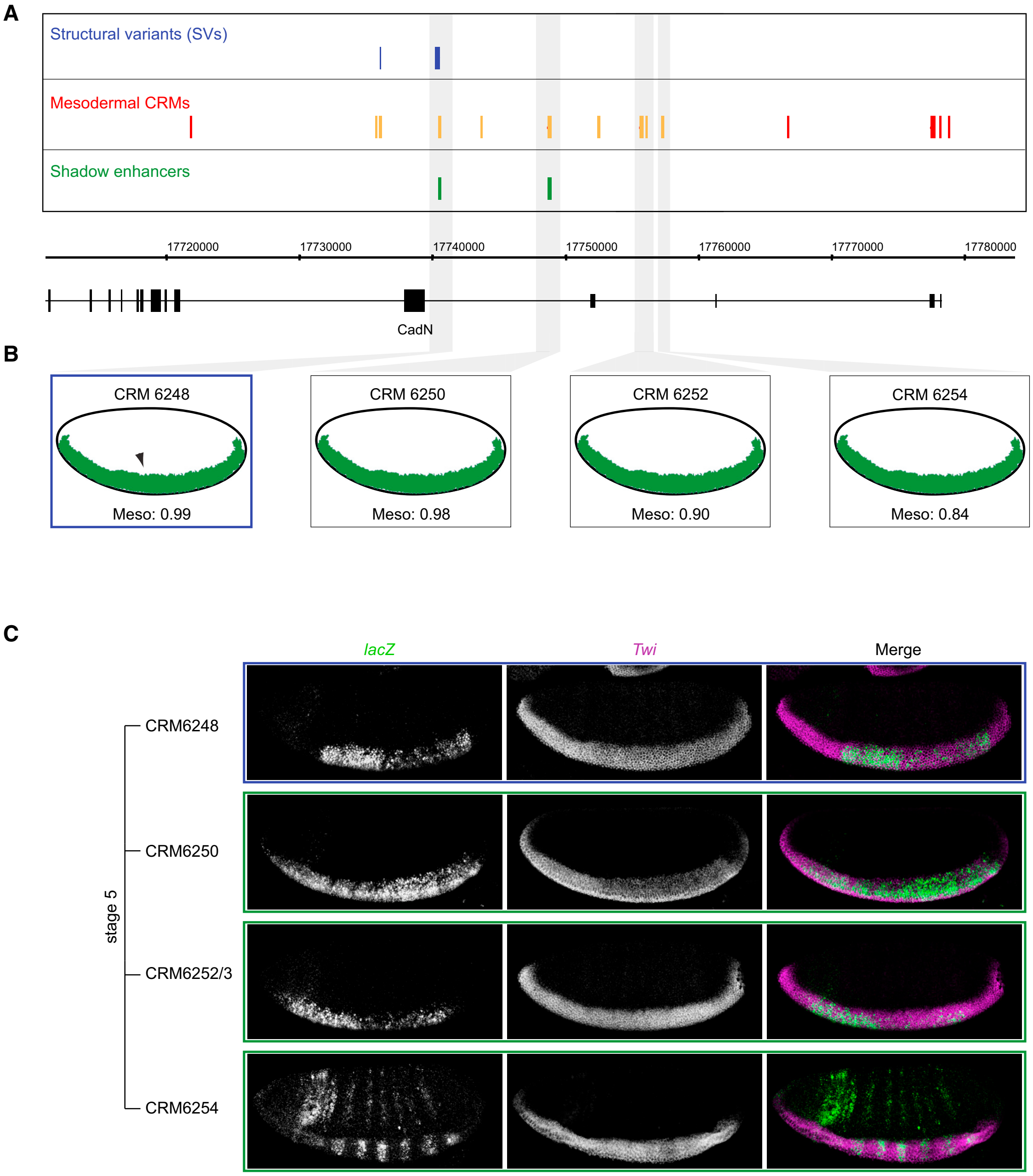

Figure 6. *CadN* Locus Has Many Enhancers with Partially Overlapping Activity

(A) *CadN* locus showing structural variants (blue), mesodermal *cis*-regulatory modules (CRMs; red), and shadow enhancers (green). Enhancers tested in transgenic embryos are indicated in orange.

(B) Shadow enhancers predicted based on both similarity in activity and correlated TF occupancy. The heatmap shows ChIP peak height signal for each factor/time point. SVM prediction and score are shown above.

(C) Double FISH of transgenic embryos showing *lacZ* reporter (green) under the transcriptional control of four enhancers (CRM6248, CRM6250, CRM6252/3, and CRM6254) with the early mesoderm marker *Twist* (*Twi*; magenta). CRM6248 is partially deleted by an SV (blue, A) and has overlapping expression with CRM6250 and two additional CRMs just below the applied cutoff (CRM6252 and CRM6254) in the presumptive mesoderm. Double FISH for *lacZ* (green) and the marker expressed early in the mesoderm Twist (magenta).

All embryos oriented with anterior to the left and dorsal at the top. See also Figure S2.

is not only present in the loci of TFs, which are a class of proteins known to have complex transcriptional regulation [50], but is also prevalent in loci for a wide range of essential genes. Here, we purposely chose gene loci of proteins with diverse function; CadN is an adhesion protein, Traf1 a signaling receptor, Rols is an intracellular adaptor protein, Ade5 is a metabolic protein, and CG6966 protein is a predicted component of the E3 ubiquitin-protein ligase complex.

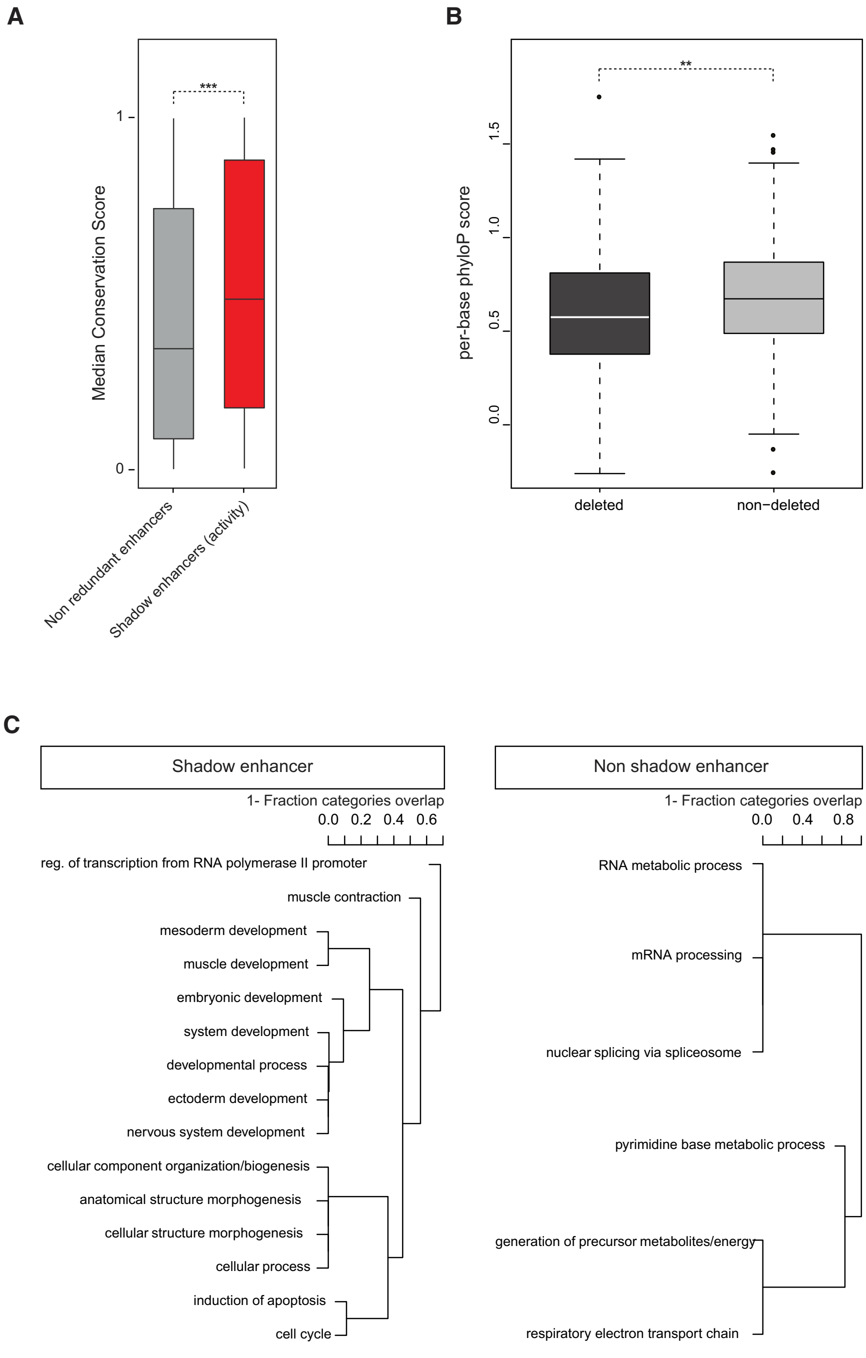

Figure 7. Conservation and Selection of Shadow Enhancers

(A) Difference in conservation level (median PhastCons) of shadow versus non-redundant enhancers (Wilcoxon rank-sum test, ***$p < 0.001$).

(B) Difference in conservation level between shadow enhancers deleted or not by segregating SVs within a natural population (Wilcoxon rank-sum test, **$p < 0.01$).

(C) Biological process enrichment for genes with shadow enhancers; significant terms are shown (based on Fisher's exact test, $p < 0.05$).

See also Figure S1.

Conservation of Shadow Enhancers

This high-confidence set represents the largest collection of shadow enhancers in any species to date and thereby enables an initial comprehensive analysis of their general properties. We therefore examined several predictions concerning shadow enhancers: (1) If they are truly redundant, they should show relaxed selective constraints relative to non-redundant enhancers. (2) If shadow enhancers act as substrates for the evolution of regulatory novelty [2] and are pervasive features of gene regulatory networks, they should be frequently associated with lineage-specific signatures of adaptive evolution, compared to non-redundant enhancers.

We first investigated whether there is a difference in the overall level of conservation between shadow enhancers and non-redundant enhancers, the latter being defined as enhancers associated with the same mesodermal and/or muscle genes but active in different tissues (i.e., non-shadow enhancers; Supplemental Experimental Procedures). We focused on the shadow enhancers with predicted similar activity (SVM), given the much larger size of this collection (866 elements). In contrast to what is expected for redundant elements, PhastCons (median 15-way genome conservation scores [51]) and phyloP scores indicate that shadow enhancers are more conserved than non-redundant enhancers ($p = 0.00079$ and $p = 0.00048$ for PhastCons and phyloP, respectively; Wilcoxon rank-sum test; Figure 7A). The difference in evolutionary rates is modest (2.85 substitutions per base over the 12 species phylogeny versus 2.557), but it translates to an ~10% difference in the number of substitutions, suggesting that shadow enhancers, although acting redundantly in one context, are most likely acting non-redundantly in another context or function. Interestingly, despite this overall higher level of conservation, the subset of shadow enhancers that are deleted by SVs tend to less conserved in comparison to "shadow pairs" not affected by segregating deletions ($p = 0.001719$, Wilcoxon rank-sum test; Figure 7B), suggesting that there may be different types of shadow enhancers evolving at different rates.

To more directly assess recent selective pressures influencing shadow enhancer evolution, we examined patterns of segregating genetic variation within 205 isogenic, wild-derived lines as part of the DGRP [40]. Consistent with the actions of directional selection, Tajima's D statistic, a measure of departure from the site-frequency spectrum expected under neutral

evolution [52], was significantly more negative for all mesoderm and/or muscle enhancers compared to flanking, proxy-neutral regions (on average -0.52 versus -0.38, $p < 0.00001$, Wilcoxon rank-sum test). Tajima's D values, however, do not differ significantly when these elements were separated into shadow enhancers and non-redundant enhancers or among our sets of proxy neutral regions. If selective pressures were relaxed on a subset of shadow enhancers, we would expect Tajima's D scores to be more heterogeneous. However, we saw no evidence to support this trend using Tajima's D or multiple alternative summary statistics (Supplemental Experimental Procedures). In short, we see little evidence from summary statistics indicating that shadow and non-redundant enhancers are evolving under different selective regimes.

We next estimated the fitness consequences of mutations within shadow enhancers and non-redundant enhancers using INSIGHT [53], a probabilistic model that partitions mutations in putative regulatory sites into coarse-grained fitness categories (neutral, weak negative, strong negative, or positive selection). In addition, INSIGHT also infers the fraction of sites with selective effects within categories of sites, a term that can be interpreted as the probability that a mutation in a given region will impact fitness (fitCons score [54]). As this measure is based on current patterns of segregating variation, it is expected to be robust to turnovers in functional sites that may occur between species. In addition to our mesodermal enhancers, we also applied INSIGHT to a large set of genomic regions that function as developmental enhancers in transgenic assays and overlap DNase-hypersensitive sites [32], as well as a set of negative regions that showed no evidence of functioning as enhancers in vivo [32]. Finally, we contrasted this with a set of 100,000 randomly chosen proxy-neutral sites that lie outside of phastCons blocks, known exons, peaks of H3K4me1, or DNase-hypersensitive sites.

For all sites, we saw a significant elevation in fitCons score relative to proxy-neutral sites, with an estimated 46%–65% of sites having a potential impact on fitness, in line with previous estimates of constraint in *Drosophila* non-coding DNA (e.g., 49.3% estimated by P. Andolfatto [55]). As expected, this fraction was lowest for genomic regions that do not function as embryonic enhancers, although still significantly higher than inferred for most classes of human regulatory DNA [54], reflecting the more compact *Drosophila* genome. Among all enhancers examined, the fitCons score was lowest for non-redundant enhancers (0.54 versus 0.59 for shadow enhancers), suggesting a higher average fitness consequence for mutations occurring in shadow enhancers. Interestingly, this difference does not reflect differences in TF occupancy for these regions, with approximately equal average occupancy for shadow and non-redundant enhancers (mean TF binding 7.26 versus 8.10). Together, these results suggest that despite similar properties of TF occupancy, mutations in shadow enhancers are slightly more likely to impact fitness than are mutations in non-redundant enhancers.

Shadow enhancers have been suggested to serve as substrate for the evolution of regulatory novelty [2]. Although challenging to test in full, if shadow enhancers frequently serve as substrate for adaptive evolution, their DNA sequences should show an enrichment of (lineage-specific) signatures for positive selection. As our INSIGHT analyses did not identify a meaningful number of positively selected sites in any enhancer set, we sought additional information in between-species substitution rates. Specifically, we tested for lineage-specific accelerated evolution at these enhancers by contrasting patterns of nucleotide evolution within regions with neutral evolution (inferred from 4-fold degenerate codon positions [4d sites]) using a phylogenetically aware likelihood framework [56]. Consistent with pervasive negative selection, few regions (0.2%–1.5%) showed evidence for phylogeny-wide acceleration relative to 4d sites, with no significant differences in proportion among shadow versus non-redundant enhancers. A higher fraction of non-redundant enhancers, however, showed evidence ($p < 0.05$, likelihood ratio test) for lineage-specific acceleration (relative to subtree evolutionary rates) along the *D. melanogaster* lineage (8.2% and 11.1% for shadow and non-redundant, respectively), though the contrast between shadow and non-redundant enhancers is not statistically significant. Although the significance threshold for accelerated evolution applied here ($p = 0.05$) does not provide strong evidence for pervasive adaptation, the results do suggest that signatures for adaptive change are marginally more common among non-redundant enhancers than shadow enhancers.

To explore the reason for this difference in conservation, we assessed Gene Ontology (GO) enrichment among genes associated with shadow enhancers compared to non-redundant enhancers (defined as genes with enhancers with non-overlapping expression in any tissue). The six most highly enriched gene sets are all related to development (Figure 7C; Table S2). Given the limited background (~600 genes with one or more non-redundant enhancers), these results are not significant after multiple testing. However, we note that as the significant categories are highly overlapping (Figure 7C), multiple testing corrections may be overly harsh in this context. We thus present all nominally significant categories (Table S6), which are highly consistent with previous studies [57]. Conversely, genes associated with non-redundant enhancers (i.e., enhancers within a gene's locus that regulate different patterns of expression) tend to be enriched for housekeeping function (Table S3); thus, despite the fact that both sets of genes are active in the developing mesoderm and derivatives, only genes associated with shadow enhancers are enriched for developmental terms. This suggests that the higher level of conservation observed for shadow enhancers (Figure 7A) may be partly explained by their association with key developmental genes, known to have more complex and conserved regulatory landscapes.

Together, these results suggest that while shadow enhancers may, in some cases, compensate for mutations affecting their partner, they are not redundant in the strict definition—contrary to expectation for elements with absolute redundancy, shadow enhancers are maintained by selection to the same, or an even greater degree, than non-redundant enhancers and show no evidence for lineage-specific adaptation, suggesting that they may have essential functions in their own right.

DISCUSSION

The presence and function of redundant elements in the regulation of the gene expression has been discussed with interest over the past two decades. In the context of embryonic

development, there are a number of examples where two enhancers act in an apparently redundant manner to regulate the expression of well characterized gene loci [1, 4, 6–9, 58]. Recent work suggests that these shadow enhancers play an important role in providing spatial precision, temporal synchrony, and generating generalized robustness to gene expression, thus uncovering more complex regulatory functions [3, 6, 7, 26, 27]. However, the frequency of redundant enhancers and the types of evolutionary forces that shape them remain poorly understood. Here we present a systematic genome-wide assessment of the extent and complexity of shadow enhancers within gene loci during embryonic development. Our results reveal that shadow enhancers (i.e., elements with similar overlapping spatial activity) are a fundamental component of developmental genes' regulatory landscape and go far beyond a simple ''two redundant enhancer'' model. Interestingly, we find that the selective constraint on these shadow enhancers appears to be as great as or greater than that for non-redundant enhancers, highlighting the fact that although shadow enhancers may have an important role in buffering development, they are far from dispensable.

Shadow Enhancers Are Pervasive throughout the Genome

This systematic assessment of the extent of redundant enhancer activity indicates that shadow enhancers are much more common, and in more complex relationships, than currently envisaged. Based on our stringent criteria, we identified 1,055 shadow enhancers. However, our extensive in vivo analysis indicates that this is almost certainly an underestimate of the extent of redundancy: when we tested the activity of regions just below the cutoffs used to define shadow enhancers, for example in the loci of *CadN*, *rols*, and *Traf1* genes, we observed that many additional elements also have similar spatial activity. This is supported by a previous study examining the occupancy of the TF Dorsal, which estimated that one-third of its target genes may contain a redundant enhancer [37]. That study and ours focused on enhancers bound by a small repertoire of TFs: extrapolating to all ~700 or so predicted *Drosophila* TFs suggests that shadow enhancers are prevalent throughout the genome and therefore could have a substantial impact on the robustness of gene expression during embryonic development. As we discuss below, however, this largely hidden layer is not without primary function, but rather may play a fundamental role in ensuring the precision, timing, and robustness of specific developmental programs, as has recently been shown at individual gene loci [3, 7, 26]. Just as promoter variants that lead to transcriptional noise are suppressed within natural populations, as seen in yeast [29], shadow enhancers may play a crucial role in the suppression of transcriptional noise during embryonic development.

How Are Redundant Enhancers Maintained during Evolution?

The partially overlapping activity of redundant enhancers appears to be an emerging theme, but one with an evolutionary paradox. In agreement with the strict definition of redundancy, deletion of a redundant enhancer does not cause major phenotypic alteration, at least in a given environmental condition, as one or more redundant elements could compensate for the loss. What then prevents the deletion of shadow enhancers with a population?

The answer may lie in the context specific nature of their redundancy, which we are referring to here as partial redundancy. As these elements drive overlapping patterns of expression, there are at least some tissues, stages, or environmental conditions in which the elements have distinct functional roles. The overlap in activity (similar expression pattern) can be restricted to a small time window or a small number of cells, while other shadow enhancer ''pairs'' may have extensive overlap in time or space (Figures 4 and 5). Thus, although an enhancer may be redundant with another element in one tissue or developmental stage, its activity may be non-redundant in another cell type and therefore be essential for embryonic development. Similarly, enhancers that appear redundant in ''normal'' environmental conditions could act non-redundantly when the environmental conditions become more extreme, as observed in the *svb* locus [3]. It is this partially redundant property that most likely holds the key to how these elements are maintained over long evolutionary periods.

A previous study hypothesized that there may be different evolutionary pressure on two redundant enhancers: the primary enhancer being more constrained than the redundant shadow enhancer, allowing the later to accumulate mutations without inducing a phenotype and thus evolve faster [2]. Our analyses of sequence conservation and the frequency of segregating mutations affecting these enhancers doesn't support this, at least in the context of these mesoderm/muscle enhancers; the evolutionary pressures affecting shadow enhancers are similar and overall show a stronger tendency toward conservation than non-redundant enhancers driving similar expression with no evidence for an increased frequency of relaxed selection or adaptive evolution, although we appreciate that these approaches are most likely underpowered to detect recent adaptive changes. Taken together, our results suggest that shadow enhancers are being maintained for a purpose. One property of many shadow enhancers, in addition to their similar overlapping activity, is that the majority also have additional non-redundant activity, which may be under selective pressure, as discussed above. Alternatively, ''redundant'' enhancers driving similar spatiotemporal activity could act together to guarantee that a gene reaches a certain level of expression [28], or could have essential roles in ensuring correct patterning precision [5, 26], or to reduce stochastic effects on gene expression [7], and thereby play an essential role in reducing transcription noise during development. In these cases, shadow enhancers ensure robustness of the trait when environmental variations occur but do not confer genetic robustness to all possible mutations since, for example, deletions of a partially redundant enhancer can drastically influence the viability of an organism [26].

We therefore argue that shadow enhancers are pervasive throughout the genome and provide robustness to gene expression in the context of fluctuating genetic and environmental perturbations. The redundant function of these enhancers, e.g., similar overlapping expression, may provide opportunities for evolutionary innovation; however, the non-redundant part of the enhancer's activity, e.g., in space, time, or environmental conditions, indicates that they also have independent functional roles, which may help to fix these elements within a population.

In summary, the data presented here indicate that almost any developmental gene can have multiple shadow enhancers, each with similar overlapping windows of activity. The combined action of partially redundant enhancers may thereby represent a significant strategy through which an organism reaches robustness during embryonic development. The extensive nature of the overlap of these elements activity will generate distributed robustness within large developmental gene regulatory networks, a role that has yet to be explored. Their prevalence may give insights into how gene regulatory networks are organized—with the modular nature of enhancers (i.e., the building blocks of gene regulatory architecture) required to produce robust and precise patterns perhaps providing redundancy (mutational robustness) as a useful byproduct.

SUPPLEMENTAL INFORMATION

Supplemental Information includes Supplemental Experimental Procedures, five figures, and six tables and can be found with this article online at http://dx.doi.org/10.1016/j.cub.2015.11.034.

AUTHOR CONTRIBUTIONS

E.C. and E.E.M.F. designed the study and analyzed the results. E.C., E.H.G., and L.C. generated all transgenic lines and performed in situ hybridization and imaging. P.K. correlated TF occupancy. D.G. and P.G. performed conservation, GO enrichment, and expression similarity analysis. T.Z. and J.O.K. did SV analysis. E.C., D.G., and E.E.M.F prepared and edited the manuscript.

ACKNOWLEDGMENTS

This work was technically supported by the EMBL Advanced Light Microscopy Facility. We thank all members of the E.E.M.F. lab for discussions and comments. This work was supported by a Marie Curie ITN grant EvoNet and DFG FU 750 grant to E.E.F. and by an EMBO post-doctoral fellowship to P.K.

Received: September 20, 2015
Revised: November 16, 2015
Accepted: November 17, 2015
Published: December 10, 2015

REFERENCES

1. Degenhardt, K.R., Milewski, R.C., Padmanabhan, A., Miller, M., Singh, M.K., Lang, D., Engleka, K.A., Wu, M., Li, J., Zhou, D., et al. (2010). Distinct enhancers at the Pax3 locus can function redundantly to regulate neural tube and neural crest expressions. Dev. Biol. *339*, 519–527.
2. Hong, J.-W., Hendrix, D.A., and Levine, M.S. (2008). Shadow enhancers as a source of evolutionary novelty. Science *321*, 1314.
3. Frankel, N., Davis, G.K., Vargas, D., Wang, S., Payre, F., and Stern, D.L. (2010). Phenotypic robustness conferred by apparently redundant transcriptional enhancers. Nature *466*, 490–493.
4. Montavon, T., Soshnikova, N., Mascrez, B., Joye, E., Thevenet, L., Splinter, E., de Laat, W., Spitz, F., and Duboule, D. (2011). A regulatory archipelago controls Hox genes transcription in digits. Cell *147*, 1132–1145.
5. Perry, M.W., Boettiger, A.N., Bothma, J.P., and Levine, M. (2010). Shadow enhancers foster robustness of Drosophila gastrulation. Curr. Biol. *20*, 1562–1567.
6. Perry, M.W., Bothma, J.P., Luu, R.D., and Levine, M. (2012). Precision of hunchback expression in the Drosophila embryo. Curr. Biol. *22*, 2247–2252.
7. Perry, M.W., Boettiger, A.N., and Levine, M. (2011). Multiple enhancers ensure precision of gap gene-expression patterns in the Drosophila embryo. Proc. Natl. Acad. Sci. USA *108*, 13570–13575.
8. Cretekos, C.J., Wang, Y., Green, E.D., Martin, J.F., Rasweiler, J.J., 4th, and Behringer, R.R. (2008). Regulatory divergence modifies limb length between mammals. Genes Dev. *22*, 141–151.
9. Xiong, N., Kang, C., and Raulet, D.H. (2002). Redundant and unique roles of two enhancer elements in the TCRgamma locus in gene regulation and gammadelta T cell development. Immunity *16*, 453–463.
10. Eldar, A., Shilo, B.-Z., and Barkai, N. (2004). Elucidating mechanisms underlying robustness of morphogen gradients. Curr. Opin. Genet. Dev. *14*, 435–439.
11. Eldar, A., Dorfman, R., Weiss, D., Ashe, H., Shilo, B.-Z., and Barkai, N. (2002). Robustness of the BMP morphogen gradient in Drosophila embryonic patterning. Nature *419*, 304–308.
12. Barrière, A., Gordon, K.L., and Ruvinsky, I. (2011). Distinct functional constraints partition sequence conservation in a cis-regulatory element. PLoS Genet. *7*, e1002095.
13. Boettiger, A.N., and Levine, M. (2009). Synchronous and stochastic patterns of gene activation in the Drosophila embryo. Science *325*, 471–473.
14. Levine, M. (2011). Paused RNA polymerase II as a developmental checkpoint. Cell *145*, 502–511.
15. Raser, J.M., and O'Shea, E.K. (2004). Control of stochasticity in eukaryotic gene expression. Science *304*, 1811–1814.
16. Gertz, J., Siggia, E.D., and Cohen, B.A. (2009). Analysis of combinatorial cis-regulation in synthetic and genomic promoters. Nature *457*, 215–218.
17. Shultzaberger, R.K., Malashock, D.S., Kirsch, J.F., and Eisen, M.B. (2010). The fitness landscapes of cis-acting binding sites in different promoter and environmental contexts. PLoS Genet. *6*, e1001042.
18. Guet, C.C., Elowitz, M.B., Hsing, W., and Leibler, S. (2002). Combinatorial synthesis of genetic networks. Science *296*, 1466–1470.
19. Arias, A.M., and Hayward, P. (2006). Filtering transcriptional noise during development: concepts and mechanisms. Nat. Rev. Genet. *7*, 34–44.
20. Jaeger, J., Surkova, S., Blagov, M., Janssens, H., Kosman, D., Kozlov, K.N., Manu, Myasnikova, E., Vanario-Alonso, C.E., Samsonova, M., et al. (2004). Dynamic control of positional information in the early Drosophila embryo. Nature *430*, 368–371.
21. Manu, S., Surkova, S., Spirov, A.V., Gursky, V.V., Janssens, H., Kim, A.R., Radulescu, O., Vanario-Alonso, C.E., Sharp, D.H., Samsonova, M., and Reinitz, J. (2009). Canalization of gene expression in the Drosophila blastoderm by gap gene cross regulation. PLoS Biol. *7*, e1000049.
22. Wunderlich, Z., Bragdon, M.D., Eckenrode, K.B., Lydiard-Martin, T., Pearl-Waserman, S., and DePace, A.H. (2012). Dissecting sources of quantitative gene expression pattern divergence between Drosophila species. Mol. Syst. Biol. *8*, 604.
23. Wagner, A. (2005). Distributed robustness versus redundancy as causes of mutational robustness. BioEssays *27*, 176–188.
24. Frankel, N., Erezyilmaz, D.F., McGregor, A.P., Wang, S., Payre, F., and Stern, D.L. (2011). Morphological evolution caused by many subtle-effect substitutions in regulatory DNA. Nature *474*, 598–603.
25. Bomblies, K., Dagenais, N., and Weigel, D. (1999). Redundant enhancers mediate transcriptional repression of AGAMOUS by APETALA2. Dev. Biol. *216*, 260–264.
26. Dunipace, L., Ozdemir, A., and Stathopoulos, A. (2011). Complex interactions between cis-regulatory modules in native conformation are critical for Drosophila snail expression. Development *138*, 4075–4084.
27. Dunipace, L., Saunders, A., Ashe, H.L., and Stathopoulos, A. (2013). Autoregulatory feedback controls sequential action of cis-regulatory modules at the brinker locus. Dev. Cell *26*, 536–543.
28. Lam, D.D., de Souza, F.S.J., Nasif, S., Yamashita, M., López-Leal, R., Otero-Corchon, V., Meece, K., Sampath, H., Mercer, A.J., Wardlaw, S.L., et al. (2015). Partially redundant enhancers cooperatively maintain Mammalian pomc expression above a critical functional threshold. PLoS Genet. *11*, e1004935.

29. Metzger, B.P.H., Yuan, D.C., Gruber, J.D., Duveau, F., and Wittkopp, P.J. (2015). Selection on noise constrains variation in a eukaryotic promoter. Nature *521*, 344–347.
30. Jiménez-Delgado, S., Pascual-Anaya, J., and Garcia-Fernàndez, J. (2009). Implications of duplicated cis-regulatory elements in the evolution of metazoans: the DDI model or how simplicity begets novelty. Brief. Funct. Genomics Proteomics *8*, 266–275.
31. Cande, J., Goltsev, Y., and Levine, M.S. (2009). Conservation of enhancer location in divergent insects. Proc. Natl. Acad. Sci. USA *106*, 14414–14419.
32. Kvon, E.Z., Kazmar, T., Stampfel, G., Yáñez-Cuna, J.O., Pagani, M., Schernhuber, K., Dickson, B.J., and Stark, A. (2014). Genome-scale functional characterization of Drosophila developmental enhancers in vivo. Nature *512*, 91–95.
33. Zinzen, R.P., Girardot, C., Gagneur, J., Braun, M., and Furlong, E.E.M. (2009). Combinatorial binding predicts spatio-temporal cis-regulatory activity. Nature *462*, 65–70.
34. Brown, C.D., Johnson, D.S., and Sidow, A. (2007). Functional architecture and evolution of transcriptional elements that drive gene coexpression. Science *317*, 1557–1560.
35. Liberman, L.M., and Stathopoulos, A. (2009). Design flexibility in cis-regulatory control of gene expression: synthetic and comparative evidence. Dev. Biol. *327*, 578–589.
36. Staller, M.V., Vincent, B.J., Bragdon, M.D.J., Lydiard-Martin, T., Wunderlich, Z., Estrada, J., and DePace, A.H. (2015). Shadow enhancers enable Hunchback bifunctionality in the Drosophila embryo. Proc. Natl. Acad. Sci. USA *112*, 785–790.
37. Zeitlinger, J., Zinzen, R.P., Stark, A., Kellis, M., Zhang, H., Young, R.A., and Levine, M. (2007). Whole-genome ChIP-chip analysis of Dorsal, Twist, and Snail suggests integration of diverse patterning processes in the Drosophila embryo. Genes Dev. *21*, 385–390.
38. Maurano, M.T., Wang, H., Kutyavin, T., and Stamatoyannopoulos, J.A. (2012). Widespread site-dependent buffering of human regulatory polymorphism. PLoS Genet. *8*, e1002599.
39. Reddy, T.E., Gertz, J., Pauli, F., Kucera, K.S., Varley, K.E., Newberry, K.M., Marinov, G.K., Mortazavi, A., Williams, B.A., Song, L., et al. (2012). Effects of sequence variation on differential allelic transcription factor occupancy and gene expression. Genome Res. *22*, 860–869.
40. Mackay, T.F.C., Richards, S., Stone, E.A., Barbadilla, A., Ayroles, J.F., Zhu, D., Casillas, S., Han, Y., Magwire, M.M., Cridland, J.M., et al. (2012). The Drosophila melanogaster Genetic Reference Panel. Nature *482*, 173–178.
41. Zichner, T., Garfield, D.A., Rausch, T., Stütz, A.M., Cannavó, E., Braun, M., Furlong, E.E.M., and Korbel, J.O. (2013). Impact of genomic structural variation in Drosophila melanogaster based on population-scale sequencing. Genome Res. *23*, 568–579.
42. Huang, W., Massouras, A., Inoue, Y., Peiffer, J., Ràmia, M., Tarone, A.M., Turlapati, L., Zichner, T., Zhu, D., Lyman, R.F., et al. (2014). Natural variation in genome architecture among 205 Drosophila melanogaster Genetic Reference Panel lines. Genome Res. *24*, 1193–1208.
43. Groth, A.C., Fish, M., Nusse, R., and Calos, M.P. (2004). Construction of transgenic Drosophila by using the site-specific integrase from phage phiC31. Genetics *166*, 1775–1782.
44. Rau, A., Buttgereit, D., Holz, A., Fetter, R., Doberstein, S.K., Paululat, A., Staudt, N., Skeath, J., Michelson, A.M., and Renkawitz-Pohl, R. (2001). rolling pebbles (rols) is required in Drosophila muscle precursors for recruitment of myoblasts for fusion. Development *128*, 5061–5073.
45. Menon, S.D., and Chia, W. (2001). Drosophila rolling pebbles: a multidomain protein required for myoblast fusion that recruits D-Titin in response to the myoblast attractant Dumbfounded. Dev. Cell *1*, 691–703.
46. O'Donnell, A.F., Tiong, S., Nash, D., and Clark, D.V. (2000). The Drosophila melanogaster ade5 gene encodes a bifunctional enzyme for two steps in the de novo purine synthesis pathway. Genetics *154*, 1239–1253.
47. Cha, G.-H., Cho, K.S., Lee, J.H., Kim, M., Kim, E., Park, J., Lee, S.B., and Chung, J. (2003). Discrete functions of TRAF1 and TRAF2 in Drosophila melanogaster mediated by c-Jun N-terminal kinase and NF-kappaB-dependent signaling pathways. Mol. Cell. Biol. *23*, 7982–7991.
48. Iwai, Y., Usui, T., Hirano, S., Steward, R., Takeichi, M., and Uemura, T. (1997). Axon patterning requires DN-cadherin, a novel neuronal adhesion receptor, in the Drosophila embryonic CNS. Neuron *19*, 77–89.
49. Oda, H., Uemura, T., Harada, Y., Iwai, Y., and Takeichi, M. (1994). A Drosophila homolog of cadherin associated with armadillo and essential for embryonic cell-cell adhesion. Dev. Biol. *165*, 716–726.
50. Lenhard, B., Sandelin, A., and Carninci, P. (2012). Metazoan promoters: emerging characteristics and insights into transcriptional regulation. Nat. Rev. Genet. *13*, 233–245.
51. Siepel, A., Bejerano, G., Pedersen, J.S., Hinrichs, A.S., Hou, M., Rosenbloom, K., Clawson, H., Spieth, J., Hillier, L.W., Richards, S., et al. (2005). Evolutionarily conserved elements in vertebrate, insect, worm, and yeast genomes. Genome Res. *15*, 1034–1050.
52. Tajima, F. (1989). Statistical method for testing the neutral mutation hypothesis by DNA polymorphism. Genetics *123*, 585–595.
53. Gronau, I., Arbiza, L., Mohammed, J., and Siepel, A. (2013). Inference of natural selection from interspersed genomic elements based on polymorphism and divergence. Mol. Biol. Evol. *30*, 1159–1171.
54. Gulko, B., Hubisz, M.J., Gronau, I., and Siepel, A. (2015). A method for calculating probabilities of fitness consequences for point mutations across the human genome. Nat. Genet. *47*, 276–283.
55. Andolfatto, P. (2005). Adaptive evolution of non-coding DNA in Drosophila. Nature *437*, 1149–1152.
56. Holloway, A.K., Begun, D.J., Siepel, A., and Pollard, K.S. (2008). Accelerated sequence divergence of conserved genomic elements in Drosophila melanogaster. Genome Res. *18*, 1592–1601.
57. Capra, J.A., Erwin, G.D., McKinsey, G., Rubenstein, J.L.R., and Pollard, K.S. (2013). Many human accelerated regions are developmental enhancers. Philos. Trans. R. Soc. Lond. B Biol. Sci. *368*, 20130025.
58. Perry, M.W., Cande, J.D., Boettiger, A.N., and Levine, M. (2009). Evolution of insect dorsoventral patterning mechanisms. Cold Spring Harb. Symp. Quant. Biol. *74*, 275–279.

Current Biology
Supplemental Information

Shadow Enhancers Are Pervasive Features of Developmental Regulatory Networks

Enrico Cannavò, Pierre Khoueiry, David A. Garfield, Paul Geeleher, Thomas Zichner, E. Hilary Gustafson, Lucia Ciglar, Jan O. Korbel, and Eileen E.M. Furlong

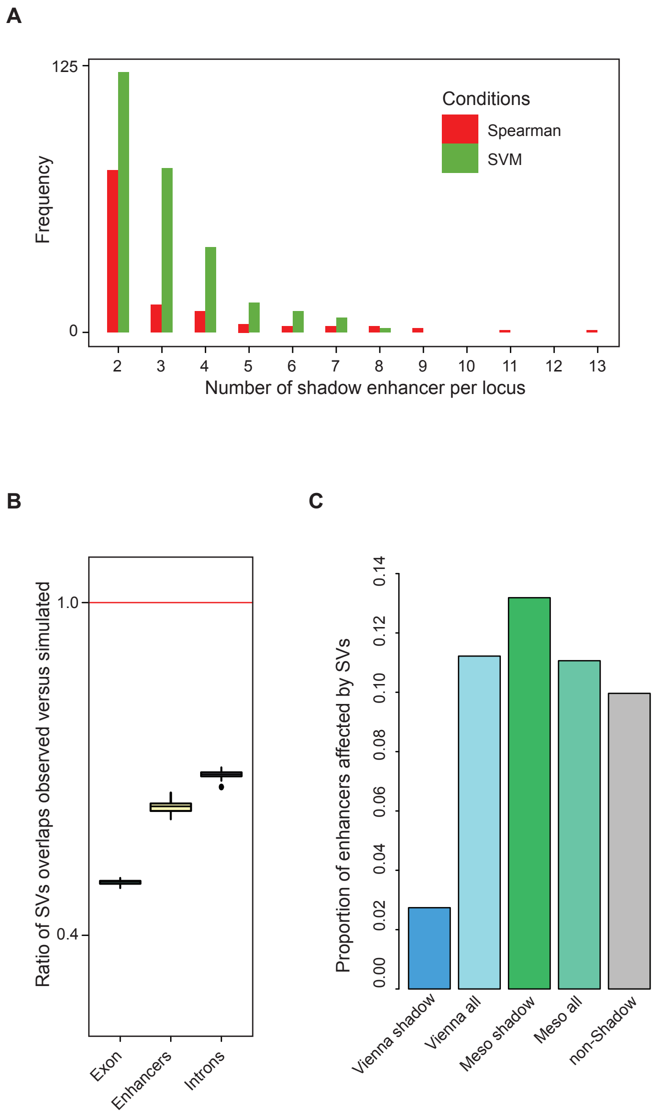

Figure S1. General properties of shadow enhancers (related to Figure 7)

A) Number of shadow enhancers (x-axis) regulating different number of genes (y-axis), identified by the SVM (blue; similarity in activity) or Spearman's correlation (red, similarity in TF occupancy). **B)** Ratio of naturally occurring SVs that overlap exons, introns and enhancers (set to one, red horizontal line), compared to the number observed when SVs are shifted randomly in +/- 50kb windows around their actual location. **C)** Proportion of deleted enhancers among various sets: shadow enhancers in the Vienna tiles versus all active Vienna enhancers (blue), mesodermal shadow enhancers (Meso, green) versus all mesodermal enhancers (green) and all non-redundant enhancers (grey).

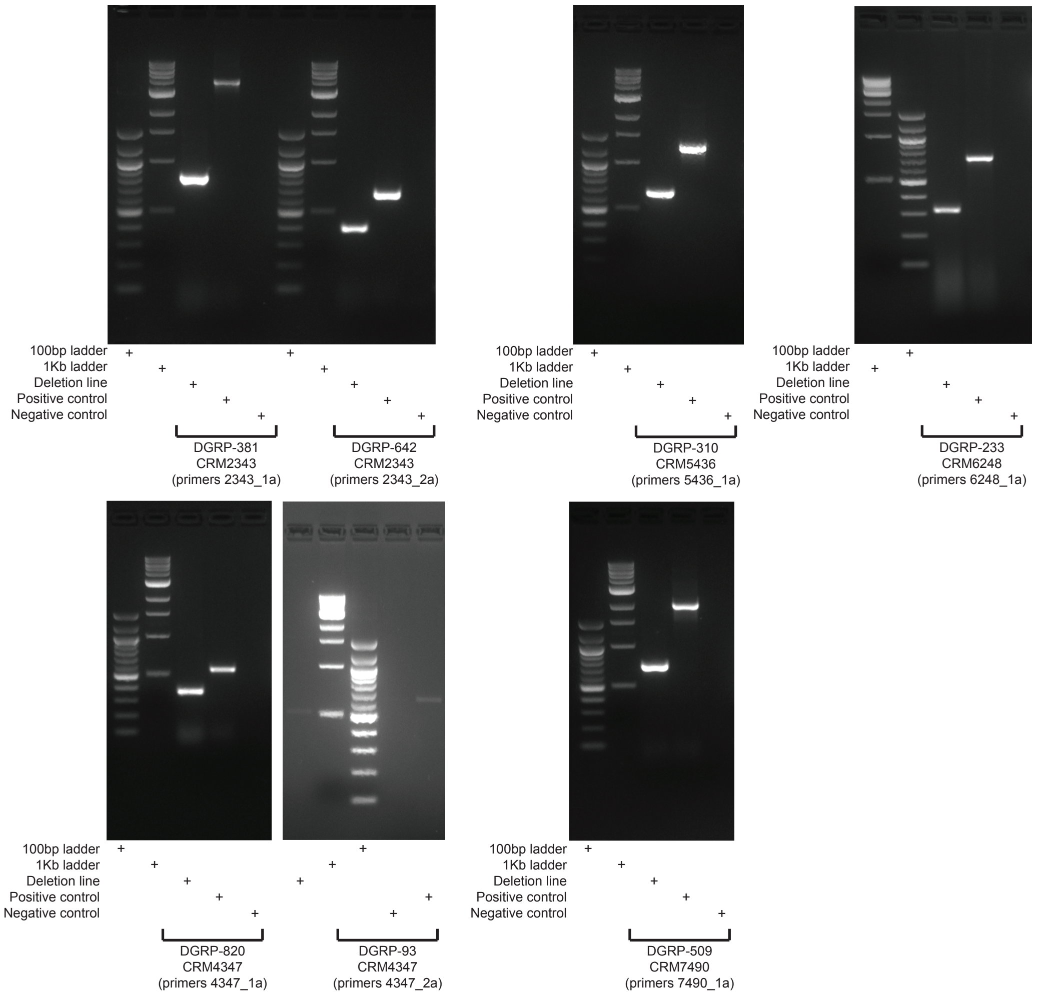

Figure S2. Validation of enhancer SV deletion by PCR (related to Figures 3-6)

We used the indicate primers (Table S5) to validate the predicted deletion of enhancers in one or two DGRP lines. DNA extracted from the reference line 2057 (Bloomington Stock Number) was used as positive control.

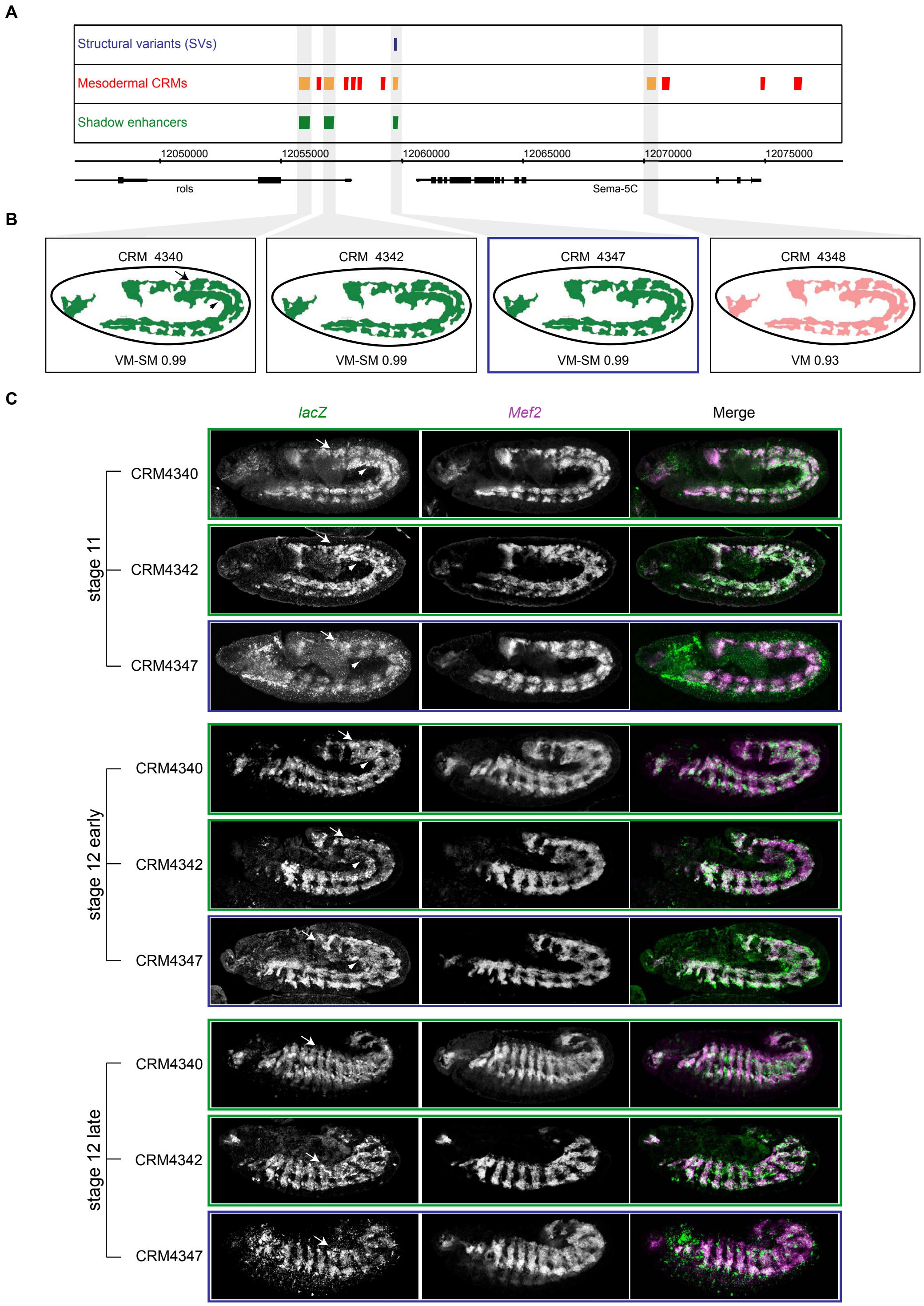
A
Structural variants (SVs)
Mesodermal CRMs
Shadow enhancers
12050000
12055000
12060000
12065000
12070000
12075000
rols
Sema-5C
B
CRM 4340
VM-SM 0.99
CRM 4342
VM-SM 0.99
CRM 4347
VM-SM 0.99
CRM 4348
VM 0.93
C
lacZ
Mef2
Merge
stage 11
CRM4340
CRM4342
CRM4347
stage 12 early
CRM4340
CRM4342
CRM4347
stage 12 late
CRM4340
CRM4342
CRM4347

Figure S3. **Shadow enhancers in the *rols* locus (related to Figure 3)**

A) *rols* locus showing structural variants (blue), mesodermal *cis*-regulatory modules defined by TF-ChIP (CRMs, red), shadow enhancers (green). Enhancers tested in transgenic embryos indicated in orange. **B)** Predicted spatial expression of enhancers. Tissue class and SVM score shown at bottom: Visceral muscle-somatic muscle (VM-SM) and Visceral muscle (VM). **C)** Double FISH of transgenic embryos showing *lacZ* reporter (green) under the transcriptional control of three shadow enhancers (CRM4340, 4342, 4347) with pan-mesoderm/muscle marker, *Mef2* (magenta). SM indicated by arrow and VM by arrowhead in B,C. CRM4347 is deleted by an SV (blue (A)) and has overlapping expression with CRM4340 and CRM4342 (B,C). Shown are embryonic stages 11 to late stage 12.

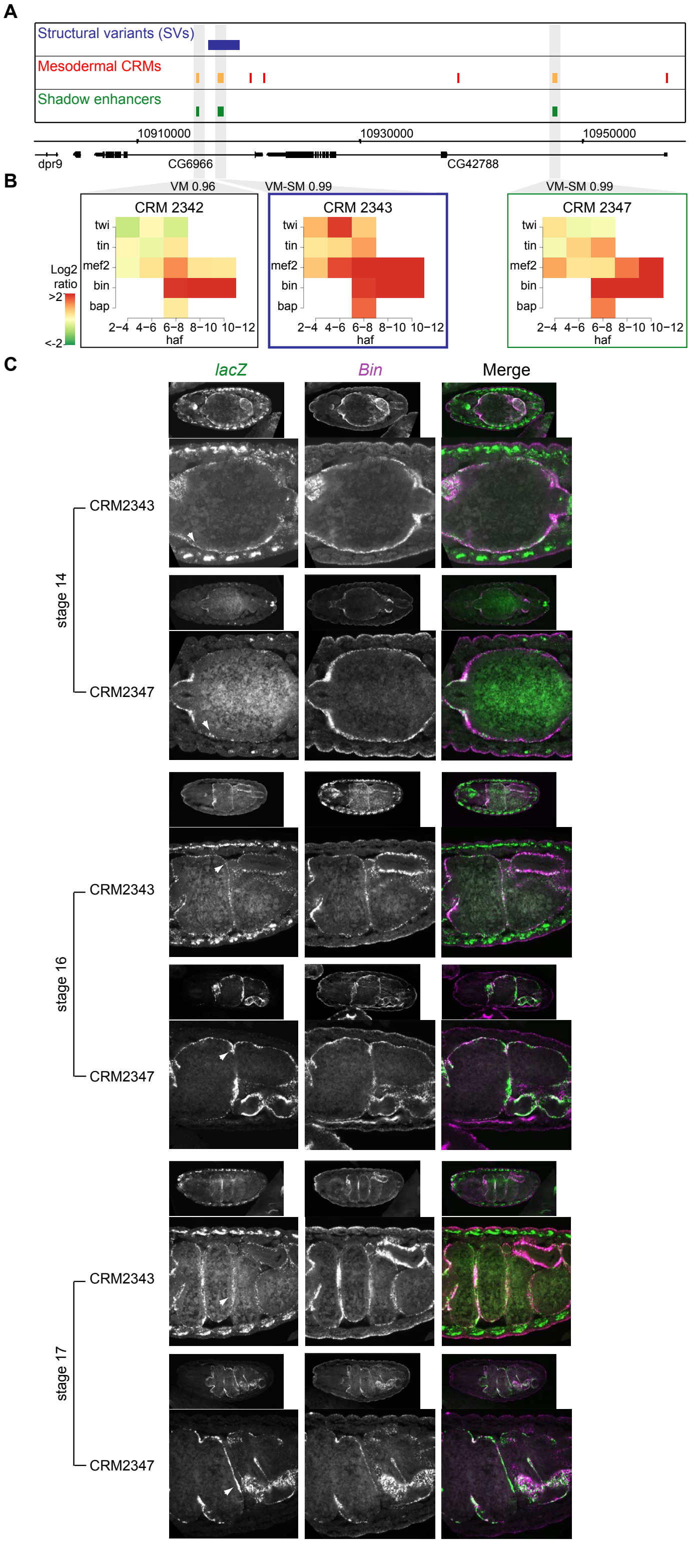

A
Structural variants (SVs)
Mesodermal CRMs
Shadow enhancers
10910000
10930000
10950000
dpr9
CG6966
CG42788
B
VM 0.96
VM-SM 0.99
VM-SM 0.99
CRM 2342
CRM 2343
CRM 2347
twi
tin
mef2
bin
bap
2-4 4-6 6-8 8-10 10-12
haf
Log2 ratio
>2
<-2
C
lacZ
Bin
Merge
CRM2343
CRM2347
stage 14
CRM2343
CRM2347
stage 16
CRM2343
CRM2347
stage 17

Figure S4. **Shadow enhancers in the *CG42788* locus (related to Figure 3)**

A) *CG42788* locus showing structural variants (blue), mesodermal *cis*-regulatory modules (CRMs, red), shadow enhancers (green). Enhancers tested in transgenic embryos indicated in orange. **B)** Three shadow enhancers predicted based on highly correlated TF occupancy, heat map shows ChIP peak height signal for each factor/timepoint. SVM prediction and score is shown above. **C)** Double FISH of transgenic embryos showing *lacZ* reporter (green) under the transcriptional control of two shadow enhancers (CRM2343, CRM2347) with the visceral muscle (VM) marker, *biniou (bin)* (magenta). VM indicated by white arrowhead. CRM2343 is completely deleted by an SV (A) and has overlapping expression with CRM2347. CRM2342 did not share regions of overlap with the other shadow enhancers. Enhancers tested in transgenic embryos indicated in orange. Embryo orientation: anterior-left, dorsal-up,

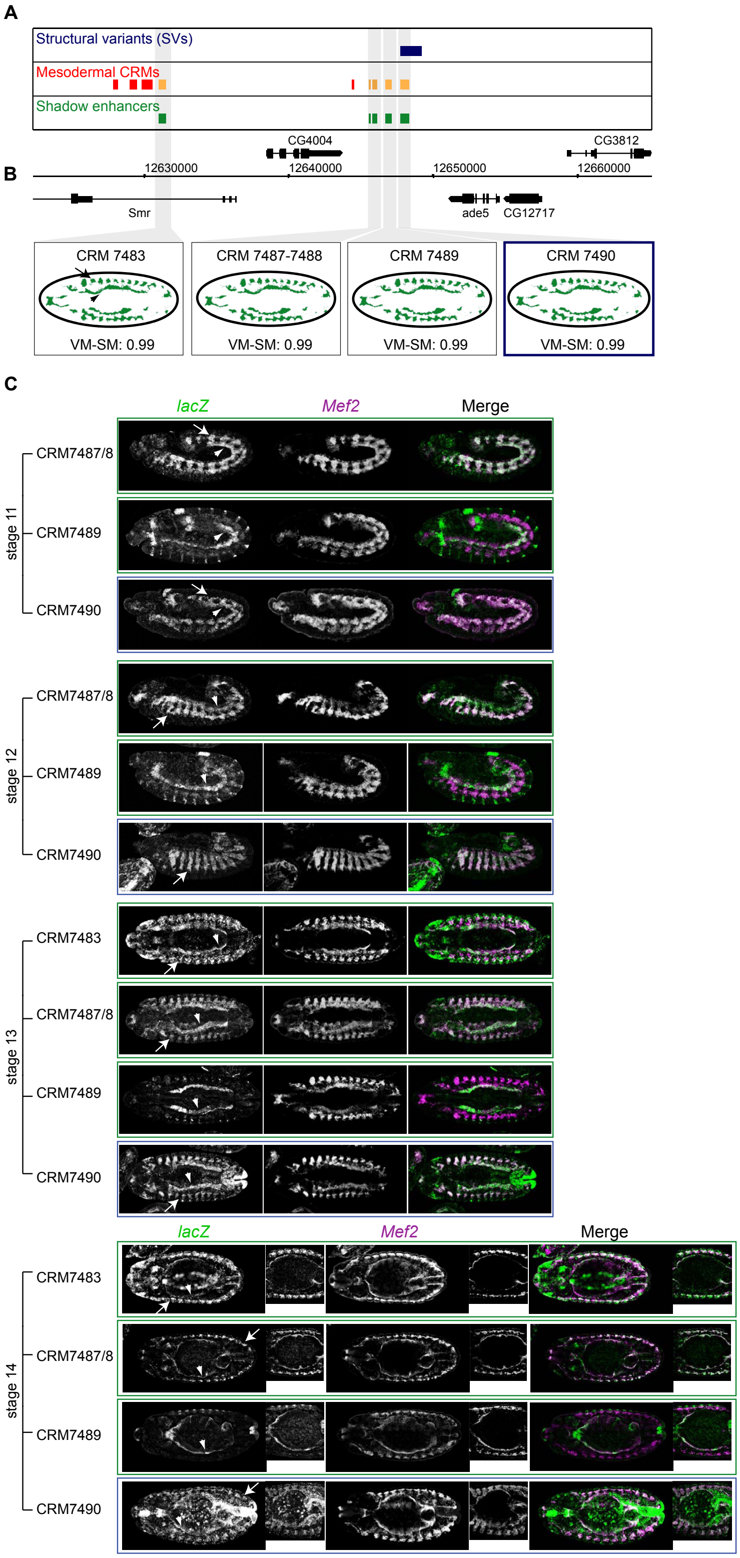
A
Structural variants (SVs)
Mesodermal CRMs
Shadow enhancers
CG4004
CG3812
B
12630000
12640000
12650000
12660000
Smr
ade5
CG12717
CRM 7483
CRM 7487-7488
CRM 7489
CRM 7490
VM-SM: 0.99
VM-SM: 0.99
VM-SM: 0.99
VM-SM: 0.99
C
lacZ
Mef2
Merge
stage 11
CRM7487/8
CRM7489
CRM7490
stage 12
CRM7487/8
CRM7489
CRM7490
stage 13
CRM7483
CRM7487/8
CRM7489
CRM7490
lacZ
Mef2
Merge
stage 14
CRM7483
CRM7487/8
CRM7489
CRM7490

Figure S5. Shadow enhancers in *ade5* locus (related to Figure 4)

A) *ade5* locus showing structural variants (blue), mesodermal *cis*-regulatory modules (CRMs, red), shadow enhancers (green). Enhancers tested in transgenic embryos indicated in orange. **B)** Predicted spatial activity of enhancers and SVM scores are shown: Visceral muscle-somatic muscle (VM-SM). **C)** Double FISH of transgenic embryos showing *lacZ* reporter (green) under the transcriptional control of four shadow enhancers (CRM7483, 7487/88, 7489, 7490) with pan-mesoderm/muscle marker, *Mef2* (magenta). CRM7490 is almost completely deleted by an SV (blue (A)) and has overlapping expression with CRM7483, CRM7487-88 and CRM7489 (green) in VM. Specifically, CRM7490, CRM7487/8 (from stage 11 to stage 14) and CRM 7483 (stage 13 and 14) show activity in somatic and visceral mesoderm while CRM 7489 is active only in the visceral mesoderm (from stage 11 to stage 14). SM indicated by arrow and VM by arrowhead in B,C. All embryos oriented anterior-left, dorsal-up.

Supplemental Tables (available online)

Table S1. Lists all shadow enhancers with their associated gene

Table S2. Lists of GO terms enriched in genes regulated by shadow enhancers

Table S3. Lists of GO terms enriched in genes regulated by non-shadow enhancers

Table S4. List of structural variants in DGRP lines

Table S5. List of used primers for SV validation

Table S6. List of GO biological processes enriched for genes regulated by shadow enhancers

Supplemental Experimental Procedures

Frequency of enhancers with redundant versus partially redundant activity

The spatial activity of 3,604 developmental enhancers examined *in vivo* was obtained from Kvon *et al*. [S1]. Each enhancer was assigned a score of '0' (inactive), or '1' (active; an intensity of ≥ 2) based on the authors annotation across each of the 227 terms at each of 6 developmental time-points (yielding an "activity vector" of 1,362 scores for each enhancer). Two enhancers were classified as exhibiting "identical" activity if their activity vectors were exactly equivalent, i.e. if both enhancers had exactly the same activity across all tissue terms and time-points. This identified 10 pairs of enhancers with both identical activity and proximity within 50kb of each other. To assess whether this was more than expected by chance, we constructed a null distribution using a permutation-based assay. For each of 1,000 permutations, the location of all enhancers was shuffled, while only swapping enhancers with similar complexity in their activity (i.e. having the same number of spatio-temporal terms), to maintain the data structure. A *P*-value was estimated based on the proportion of permutations for which the number of pairs of enhancers (within 50kb) with identical activity was less than the number observed.

Enhancers with similar activity (i.e. one or more tissue expression terms is the same, at the same stages of development, as annotated by Kvon et al [S1]) were quantified using the Euclidian distance between their corresponding "activity vectors", based on their *in vivo* activity as annotated by the authors. The global level of similarity between enhancers within 50kb of each other was quantified by the mean Euclidian distance. As above, we used a permutation-based assay to assess whether this observed number was greater or less than expected by chance. In this case, we also performed 1,000 permutations, but because the observed distance was far

smaller than those observed by random chance, the *P*-value reported was calculated from a theoretical normal distribution, the parameters of which were estimated using the 1,000 values obtained from the permutations. For gene expression, the same methods described above were applied, except the spatio-temporal activity vector for the enhancers was replaced by expression data for genes obtained from the Berkeley *Drosophila* Genome Project in-situ hybridization database.

Genome-wide identification of shadow enhancers

Shadow enhancers were predicted by two methods. The first was based on correlated TF occupancy, using 8,008 ChIP-defined *cis*-regulatory modules (CRMs) [S2]. The mean ChIP intensity profiles for each TF was calculated in a 200 bp window, with a 35 bp step [S2]. This gave a vector of 15 data points for each CRM, which was used to calculate the Spearman rank correlation coefficient for all pairs of CRMs in each chromosome. Pairs with Spearman's rho >= 0.8 within a window between 200 bp to 50kb were considered as highly similar. As these TFs are exclusively expressed in mesoderm and/or muscle tissues, we restricted our analysis to highly similar enhancer pairs associated with genes that are expressed in the same tissue (using *in-situ* hybridization data from BDGP and literature annotation from FlyBase, excluding all ubiquitous genes), as described previously [S1]. This identified 350 shadow enhancers (Table S1).

The second method, based on enhancers with similar activity, took advantage of previous tissue predictions using a machine learning approach (a Support Vector Machine (SVM) [S2]. To predict redundant enhancers, we selected pairs within a 200bp to 50 kb window with a high SVM specificity (sp>=0.95) in at least one overlapping tissue class. Enhancers were classified as having similar overlapping activity in one or combinations of 3 tissue types: (1) Mesoderm

was based on enhancers with SVM predictions in ‘Meso_only’ and ‘Meso_SM’, (2) visceral mesoderm (VM), based on SVM predictions for ‘VM_only’ and ‘VM_SM’, (3) somatic mesoderm (SM), based on SVM predictions for ‘Meso_SM’ and ‘VM_SM’, (4) mesoderm and somatic Mesoderm (Meso_SM), based on SVM predictions for ‘Meso_SM’, ‘Meso_only’ and ‘VM_SM’, (5) visceral mesoderm and somatic mesoderm (VM_SM), based on SVM predictions for ‘VM_SM’, ‘Meso_SM’ and ‘VM’. The ‘SM_only’ group was excluded as the SVM predictions were poor upon validation [S2]. The final set of shadow enhancers were those where each enhancer in a pair has predicted activity in the same tissue and is associated with a common gene that is also expressed in that tissue (using *in-situ* hybridization data from BDGP and literature annotation from FlyBase, excluding all ubiquitous genes), as described previously [S1].

This identified a stringent set of 866 shadow enhancers, associated with 298 genes. Shadow enhancers from the both approaches (Spearman and SVM based) were merged to obtain a unique set of 1125 (866 + 350 – 91 common pairs) putative shadow enhancers (Table S1). Although not used for further analysis, we also applied the same criteria to the 3604 Vienna tiles that gave activity, which identified 75 regions that classify as shadow enhances based on their overlapping expression and association to a common target gene. Of these, 2 have SVs within the DGRP collection that removes the enhancer.

Structural Variant detection

To facilitate this study we extended our previous structural variant (SV) analysis from 40 lines [S3] to 205 lines with a few changes. The variant discovery was performed as follows: We inferred deletions in all 205 lines using the four different computational tools Pindel [S4], DELLY [S5], Genome STRiP [S6], and CNVnator [S7]. Genome STRiP [S6] v1.0.4 was used

to perform simultaneous population-scale deletion discovery on the 205 DGRP samples. The minimum required mapping quality for Genome STRiP was set to 20. For Pindel (v0.2.4d) we set the maximum detectable SV size to 129,472 (parameter '-x 6') and the minimum number of matched bases to 20 ('-d 20'). For CNVnator (v0.2.2) we used a bin size of 200 bp. We then integrated the results by merging the individual variant predictions for our four methods and the published freeze2 DGRP set [S3, S8], as described previously [S3], generating a single variant list (Table S4). Based on our previous whole-genome tiling array data for six lines [S3], we estimated the false discovery rate of the final set to be ~ 15%; the fraction of true positive and potential false positive variants was determined by estimating a Gaussian mixture model. Variants where more than 90% of the bases overlap annotated repeats, based on RepeatMasker and TandemRepeatFinder predictions (both downloaded from UCSC on October 15, 2013), were removed.

Conservation analysis

Phastcons scores were obtained using the twelve *Drosophila* species, mosquito, honeybee and red flour beetle (PhastCons 15-way) [S9]. The sources were downloaded from UCSC Genome browser via ftp://hgdownload.cse.ucsc.edu/goldenPath/dm3/phastCons15way/). Non-redundant enhancers (356 elements) were defined as enhancers mapped to the same mesoderm/muscle genes, but driving expression in different tissues, so non-overlapping patterns of expression (and therefore not in a shadow pair). The average conservation scores were compared between shadow enhancers and non-redundant enhancers using a Wilcoxon rank-sum test.

PhyloP scores and expected numbers of substitutions were calculated for each base in the 15-species alignment using the LRT method (for scores) and SPH method (for substitutions) as

implemented in phyloP [S10]. Following Pollard *et al*., we used positive scores to indicate conservation, and negative scores to indicate acceleration relative to a neutral model. Neutral models were generated separately for each chromosome arm using four-fold degenerate codon positions to fit a strand-symmetric general reversible process model (REV) using the program phyloFit [S11]. As the base composition of 4d sites differ rather dramatically from the general base-composition of the non-coding genome, we adjusted our neutral models to reflect the G+C content of the non-coding genome on each chromosome arm, as recommended by Pollard *et. al* [S10]. BigWig file versions of the phyloP results are available upon request.

To estimate more current selective forces acting on these enhancers, we made use of Tajima's D statistics. For this analysis, we divided the 8008 ChIP-CRMs into three groups: the two sets of shadow enhancers (for a total of 1125) and a set of non-redundant enhancers (356) as described above (conservation analysis). For each element we also defined proxy neutral regions consisting of 500bp on either side of the element followed by the removal of any bases found within DNase hypersensitive sites [S3], transcribed genes/ncRNAs (FlyBase release 5.57), or peaks of H3K4me1. For each element, and its flanking neutral proxies, Tajima's D was calculated using custom Python scripts [S12], and the resulting distributions were compared. With these same scripts, we also calculated Fu and Li's D, Fu and Li's F, Fay and Wu's H, as well as HKA statistics (using flanking sequence as locus 2) using *D. simulans* and *D. erecta* as out-groups with qualitatively similar (i.e. non-significant) results.

Although useful for broad views, summary statistics are limited in their ability to adjust for differences in local mutation rates and in their incorporation of information from different parts of the site-frequency spectrum (*e.g.* the contribution of weak negative selection to rare segregating mutations). We thus turned to a probabilistic model, INSIGHT [S13], which

partitions putative regulatory sites into coarse-grained fitness categories (neutral, weak negative, strong negative, or positive selection) using patterns of polymorphism and divergence between these sites and flanking, neutral sequence. The model also infers the fraction of sites with selective effects, a term that can be interpreted as the probability (fitCons score, [S14]) that a mutation will impact fitness.

As sequencing errors in closely related out groups can strongly influence both INSIGHT and our lineage-specific tests for selection, we constructed a custom 12-way *Drosophila* alignment in which *D. simulans* and *D. sechellia* were represented by two recent, second generate assemblies of the original reference lines ([S15], [S16], respectively). Both genomes were aligned to the UCSC dm3 assembly using TBA and combined with the remaining nine species (plus *D. melanogaster*) using Multiz [S17]. The resulting alignments, liftover chain files, and alignment parameters are available upon request. To reduce artifacts due to alignment errors in our lineage-specific tests, we masked all repetitive sequences. For INSIGHT analyses, we additionally masked and excluded sites associated with indels and regions of low coverage (less than one read per sample on average) in the DGRP.

INSIGHT analyses was conducted following the methods described in the original paper [S13]. Briefly, we calculated our baseline evolutionary models by fitting a General Time Reversible model to 4d-sites across our alignment using the program phyloFit in the PHAST package [S11]. This model was fit to each chromosome arm independently, though the results varied little in practice for the euchromatic regions of the genome. To obtain the conditional probability of the ancestral nucleotide sequences, we first masked the *D. melanogaster* reference genome and obtained the "posterior" ancestral state probabilities using phyloFit/prequel in the PHAST package. To account for local variation in mutation rates and coalescent times, we tiled

the genome with 5kb overlapping windows within which we masked all sites contained within phastCons blocks or within 25bp of exonic sequence, DNaseI peaks, peaks of H3Kme3, or containing genotype information for fewer than 400 haplotypes (200 diploid individuals) to generate a neutral reference. We then sampled randomly at each base 400 haplotypes and used these sites to calculate an estimate of the population parameter theta (Waterson's estimator) with polymorphic sites in the block contributing to INSIGHT's global estimate of expected neutral allele frequencies. Within each block, we also obtained block-specific estimates of the divergence rate along the *D. melanogaster* lineage by masking all sites polymorphic in *D. melanogaster* and estimating a new, *D. mel*-specific scaling factor for our original neutral tree using phyloFit. We then associated each of these estimates with a unique, non-overlapping, 2.5kb window. These block-specific parameters were then stored in a central database to be accessed for individual INSIGHT runs.

Lineage-specific tests for selection were carried out on each regulatory element independently using the program phyloP [S10], which carries out formal likelihood-ratio tests for evolutionary scenarios (including branch-specific acceleration relative to a subtree) using as a null evolutionary models estimated from neutral sequences (in this case, 4d-sites). For these tests, we constructed neutral models as described above, but allowing four rate categories drawn from a discrete gamma distribution to better reflect the diversity of potential selective forces acting in any given region.

Deletion validation and CRM cloning

The deletion of shadow enhancers by structural variants in the five loci was validated by PCR. Genomic DNA was phenol-chloroform extracted from ~30 adult flies in isogenic DGRP lines

that deleted the enhancer. We performed PCR using 200 ng of genomic DNA (primers provided in Table S5) from the isogenic DGRP line and a reference line (Bloomington Drosophila Stock Center, stock number: 2057).

Enhancers were directionally cloned into the previously described pDuo2n – attB vector [S2] in the MCS cassette upstream the *lacZ* reporter gene, using BglII, KpnI, or AscI restriction sites. The sequences of all enhancers were validated through standard Sanger sequencing at both the plasmid cloning step and from the final fly stock.

Transgenic reporter assay

All constructs were injected with standard methods in line J27 (Basler lab [S18]) so that integration occurred in chromosomal position 51C. Transgenic lines were balanced and homozygosed on chromosome 2 and tested by multiplex fluorescent in situ hybridization (ISH) using anti-sense RNA probes labeled with Digoxigenin (DIG), Biotin (BIO), or Fluorescein (FITC), which were developed using tyramide signal amplification. Enhancer activity was visualized by fluorescent in situ hybridization against *lacZ* (green channel) with appropriate marker genes (magenta); *twist* (*twi*) for early mesoderm, *biniou* (*bin*) for the VM, *Mef2* for a general mesoderm/muscle marker. All images were taken on a Zeiss LSM 510 META confocal microscope.

Supplemental references

S1. Kvon, E. Z., Kazmar, T., Stampfel, G., Yáñez-Cuna, J. O., Pagani, M., Schernhuber, K., Dickson, B. J., and Stark, A. (2014). Genome-scale functional characterization of Drosophila developmental enhancers in vivo. Nature *512*, 91–95.

S2. Zinzen, R. P., Girardot, C., Gagneur, J., Braun, M., and Furlong, E. E. M. (2009). Combinatorial binding predicts spatio-temporal cis-regulatory activity. Nature *462*, 65–70.

S3. Zichner, T., Garfield, D. A., Rausch, T., Stütz, A. M., Cannavo, E., Braun, M., Furlong, E. E. M., and Korbel, J. O. (2013). Impact of genomic structural variation in Drosophila melanogaster based on population-scale sequencing. Genome Res *23*, 568–579.

S4. Ye, K., Schulz, M. H., Long, Q., Apweiler, R., and Ning, Z. (2009). Pindel: a pattern growth approach to detect break points of large deletions and medium sized insertions from paired-end short reads. Bioinformatics *25*, 2865–2871.

S5. Rausch, T., Zichner, T., Schlattl, A., Stütz, A. M., Benes, V., and Korbel, J. O. (2012). DELLY: structural variant discovery by integrated paired-end and split-read analysis. Bioinformatics *28*, i333–i339.

S6. Handsaker, R. E., Korn, J. M., Nemesh, J., and McCarroll, S. A. (2011). Discovery and genotyping of genome structural polymorphism by sequencing on a population scale. Nat Genet *43*, 269–276.

S7. Abyzov, A., Urban, A. E., Snyder, M., and Gerstein, M. (2011). CNVnator: an approach to discover, genotype, and characterize typical and atypical CNVs from family and population genome sequencing. Genome Res *21*, 974–984.

S8. Huang, W., Massouras, A., Inoue, Y., Peiffer, J.,Ràmia, M., Tarone, A. M., Turlapati, L., Zichner, T., Zhu, D., Lyman, R. F., et al. (2014). Natural variation in genome architecture among 205 Drosophila melanogaster Genetic Reference Panel lines. Genome Res *24*, 1193–1208.

S9. Siepel, A. (2005). Evolutionarily conserved elements in vertebrate, insect, worm, and yeast genomes. Genome Res *15*, 1034–1050.

S10. Pollard, K. S., Hubisz, M. J., Rosenbloom, K. R., and Siepel, A. (2010). Detection of nonneutral substitution rates on mammalian phylogenies. Genome Res *20*, 110–121.

S11. Siepel, A., and Haussler, D. (2004). Phylogenetic estimation of context-dependent substitution rates by maximum likelihood. Mol Biol Evol *21*, 468–488.

S12. Garfield, D., Haygood, R., Nielsen, W. J., and Wray, G. A. (2012). Population genetics of cis-regulatory sequences that operate during embryonic development in the sea urchin Strongylocentrotus purpuratus. Evol Dev *14*, 152–167.

S13. Gronau, I., Arbiza, L., Mohammed, J., and Siepel, A. (2013). Inference of natural

selection from interspersed genomic elements based on polymorphism and divergence. Mol Biol Evol *30*, 1159–1171.

S14. Gulko, B., Hubisz, M. J., Gronau, I., and Siepel, A. (2015). A method for calculating probabilities of fitness consequences for point mutations across the human genome. Nat Genet *47*, 276–283.

S15. Hu, T. T., Eisen, M. B., Thornton, K. R., and Andolfatto, P. (2013). A second-generation assembly of the Drosophila simulans genome provides new insights into patterns of lineage-specific divergence. Genome Res *23*, 89–98.

S16. Coolon, J. D., McManus, C. J., Stevenson, K. R., Graveley, B. R., and Wittkopp, P. J. (2014). Tempo and mode of regulatory evolution in Drosophila. Genome Res *24*, 797–808.

S17. Blanchette, M., Kent, W. J., Riemer, C., Elnitski, L., Smit, A. F. A., Roskin, K. M., Baertsch, R., Rosenbloom, K., Clawson, H., Green, E. D., et al. (2004). Aligning multiple genomic sequences with the threaded blockset aligner. Genome Res *14*, 708–715.

S18. Bischof, J., Maeda, R. K., Hediger, M., Karch, F., and Basler, K. (2007). An optimized transgenesis system for Drosophila using germ-line-specific phiC31 integrases. Proc Natl Acad Sci USA *104*, 3312–3317.
